# Supplementary material for: Ancient gene linkages support ctenophores as sister to other animals
Source: Nature. 2023 May 17;618(7963):110–7. doi: 10.1038/s41586-023-05936-6 (PMC10232365; doi:10.1038/s41586-023-05936-6)
Supplement: Supplementary file 1 — Supplementary Methods, Supplementary Tables, Supplementary Figures, Supplementary Results and Discussion, and Supplementary Notes. [file 41586_2023_5936_MOESM1_ESM.pdf]

nature portfolio

https://doi.org/10.1038/s41586-023-05936-6

Supplementary information

Ancient gene linkages support ctenophores as sister to other animals

In the format provided by the authors and unedited

Supplementary Information Guide

Ancient gene linkages support ctenophores as sister to other animals

Darrin T. Schultz[1,2,3], Steven H.D. Haddock[2,4], Jessen V. Bredeson[5], Richard E. Green[3], Oleg Simakov[1], Daniel S. Rokhsar[5,6,7]

Affiliations:

[1]Department of Molecular Evolution and Development, University of Vienna, Vienna, 1010, Austria
[2]Monterey Bay Aquarium Research Institute, Moss Landing, California 95039, United States.
[3]Department of Biomolecular Engineering and Bioinformatics, University of California, Santa Cruz, California 95064, United States.
[4]Department of Ecology and Evolutionary Biology, University of California, Santa Cruz, California 95064, United States.
[5]Department of Molecular and Cell Biology, University of California, Berkeley, CA, 94720 USA
[6]Molecular Genetics Unit, Okinawa Institute of Science and Technology Graduate University, 1919-1, Tancha, Onna, Okinawa, 904-0495 Japan
[7]Chan Zuckerberg Biohub, 499 Illinois St, San Francisco, CA, 94158 USA

*Correspondence to:

ORCID (alphabetical):

Bredeson, Jessen V.	https://orcid.org/0000-0001-5489-8512
Green, Richard E.	https://orcid.org/0000-0003-0516-5827
Haddock, Steven H.D.	https://orcid.org/0000-0001-9420-4482
Rokhsar, Daniel S.	https://orcid.org/0000-0002-8704-2224
Schultz, Darrin T.	https://orcid.org/0000-0003-1190-1122
Simakov, Oleg	https://orcid.org/0000-0002-3585-4511

This PDF file includes:

- Supplementary Information, containing:
  - Supplementary Methods
  - Supplementary Tables
  - Supplementary Figures
  - Supplementary Results and Discussion
  - Supplementary Notes

Supplied as separate files:

- Supplementary Data 1 - Sequencing library details
- Supplementary Data 2 - Ortholog tables
- Supplementary Data 3 - GO analysis
- Supplementary Data 4 - Text files of ALG mixing results
- Supplementary Data 5 - Tables of ALG mixing results
- Supplementary Data 6 - Bayesian analysis files

Description of Supplementary Data files contents

These are spreadsheets and files that are too large to be included in the main text and in the Supplementary Information (this PDF).

Index of Supplementary Data 1:

- Sequencing results, NCBI SRA accession numbers, NCBI BioSamples, NCBI BioProjects

Index of Supplementary Data 2:

- Tab 1 - COWa_HCA_EMU_RES formatted .groupby table
- Tab 2 - COWb_HCA_EMU_RES formatted .groupby table
- Tab 3 - COWc_HCA_EMU_RES formatted .groupby table
- Tab 4 - COWabc_HCA_EMU_RES composite results table
- Tab 5 - SRO_HCA_EMU_RES composite results table
- Tab 6 - CFR_HCA_EMU_RES composite results table
- Tab 7 - (CFR,COW,SRO)-HCA-EMU-RES HMM search composite table
- Tab 8 - (CFR,COW,SRO)-HCA-EMU-RES HMM search rbh table
- Tab 9 - EMU-RES HMM groupby table
- Tab 10 - EMU-RES HMM rbh table
- Tab 11 - OrthoFinder genes

Index of Supplementary Data 3:

- Table of ortholog identification with ALG plus H. sapiens proteins
- Results of GO enrichment analysis

Index of Supplementary Data 4:

- Text output of COW-HCA-EMU-RES mixing results
- Text output of HCA-EMU-RES mixing results

Index of Supplementary Data 5:

- Table of HCA-EMU-RES mixing results
- Table of COW-HCA-EMU-RES mixing results

Index of Supplementary Data 6:

- FWM_matrix_20220927.nex - contains the 3-state matrix of gene group fusions-with-mixing
- threeStateTrees/ - contains the resulting files from performing the two-state Bayesian analysis
- twoStateTrees/ - contains the files from performing the three-state Bayesian analysis

Description of files contained in the Dryad Repository

This repository contains genome assemblies, input files for the analyses using `odp`, and the odp package source code. Files used in specific analyses described in Supplementary Information sections, including small scripts/programs, are included in section-specific directories. The data are publicly available here: https://doi.org/10.5061/dryad.dncjsxm47.

Lists of Supplementary Figures and Supplementary Tables

List of Supplementary Figures

SF 2.1 | **Hexactinellid sponge genome homology**
SF 4.1 | **The assumptions of the phylogenetic methodology used in this manuscript**
SF 4.2 | **Example Oxford dot plot between two species, with significance test**
SF 4.3 | **ALGs involved in a fusion-with-mixing event**
SF 4.4 | **Phylogenetic interpretations of ALGs with unique chromosome combinations**
SF 4.5 | **An ambiguous scenario of an ancestral fusion or derived fusion**
SF 4.6 | **Phylogenetic interpretations of two ALGs mixed only in one species**
SF 4.7 | **Phylogenetic interpretations of two ALGs with one unfused species**
SF 4.8 | **Phylogenetic interpretations of an apparent derived fission**
SF 4.9 | **Phylogenetic interpretations of two ALGs fused in the outgroup**
SF 5.1 | **The three hypothesis tests of odp_genome_rearrangement_simulation**
SF 6.1 | **Automated ALG analysis recovers bilaterian synapomorphies**
SF 6.2 | **Automated ALG analysis recovers cnidarian synapomorphies**
SF 7.1 | ***Capsaspora*-ALG synteny is limited to single *Capsaspora* chromosome arms**
SF 7.2 | **The ichthyosporean Creolimax does not exhibit conserved macrosynteny with metazoans**
SF 7.3 | **Oxford dot plots comparing unicellular species**
SF 11.1 | **COW-RES unfiltered Fisher's Exact Test Results**
SF 14.1 | **Bayesian analysis of chromosome fusion states recovers ctenophore-sister**

List of Supplementary Tables

ST 1.1 | **Sequencing libraries for unicellular animal outgroup species**
ST 1.2 | **Ctenophore SRAs used for annotating the *Bolinopsis* genome**
ST 1.3 | ***Bolinopsis* genome annotation statistics**
ST 1.4 | **Genome assembly statistics**
ST 2.1 | **Cladorhizid sponge and *Ephydatia muelleri* chromosome numbering**
ST 3.1 | **Estimated centromere positions in unicellular species**
ST 4.1 | **Hypothetical orthologs and chromosome coordinates from four species**
ST 4.2 | **More hypothetical orthologs and chromosome coordinates from four species**
ST 4.3 | **ALG table of a putative fusion-with-mixing event**
ST 4.4 | **A single ALG with a unique combination of chromosomes**
ST 4.5 | **One fission event in the outgroup / One fusion in the ancestor of other species**
ST 4.6 | **ALGs of a putative derived fusion-with-mixing**
ST 4.7 | **A putative fission event in the ancestor of one species**
ST 4.8 | **Putative ancestral fission event in the ancestor of two species**
ST 4.9 | **Fusion in the branch leading to the outgroup, or an ancestral fission**
ST 7.1 | **Genomes used in macrosynteny analyses**
ST 7.2 | ***Hormiphora* chromosomes and ALG arithmetic**
ST 11.1 | **CFR-HCA ortholog-ortholog conservation score significant groupings**
ST 11.2 | **CFR-HCA ortholog network conservation score significant groupings**
ST 11.3 | **COW-RES conservation scores significant groupings**
ST 11.4 | **COW-HCA ortholog-ortholog t ≥ 0.5 conservation score table**
ST 11.5 | **COW-HCA ortholog network t ≥ 0.35 conservation score table**
ST 11.6 | **SRO-HCA ortholog-ortholog t ≥ 0.5 conservation score table**
ST 11.7 | **SRO-HCA ortholog network t ≥ 0.35 conservation score table**

Table of Contents - Supplementary Information

1. **Genome sequencing, assembly, and annotation - *Bolinopsis microptera***
    - **1.1** Methods - *Bolinopsis microptera*
        - **1.1.1** Sample collection
        - **1.1.2** DNA and RNA sequencing
        - **1.1.3** Hi-C library preparation
        - **1.1.4** Genome assembly
        - **1.1.5** Genome annotation
    - **1.2** Results and Discussion - *Bolinopsis microptera*
        - **1.2.1** Genome assembly summary
    - **1.3** Supplementary Tables - *Bolinopsis microptera*
        - ST 1.1 **| Sequencing libraries for unicellular animal outgroup species**
        - ST 1.2 **| Ctenophore SRAs used for annotating the *Bolinopsis* genome**
        - ST 1.3 **| Ctenophore genome annotation statistics**
        - ST 1.4 **| Genome assembly statistics**

2. **Genome sequencing, assembly, and annotation - Sponge genomes**
    - **2.1** Methods - Sponges
        - **2.1.1** Sample collection
        - **2.1.2** DNA and RNA sequencing
        - **2.1.3** Hi-C library preparation
        - **2.1.4** Genome assembly
        - **2.1.5** Genome annotation
        - **2.1.6** Species verification
    - **2.2** Results and Discussion - Sponges
        - **2.2.1** Species identification
        - **2.2.2** Cladorhizid sponge genome - Summary
        - **2.2.3** Hexactinellid sponge genome - Summary
    - **2.3** Supplementary Figures - Sponge genomes
        - SF 2.1 **| Hexactinellid sponge genome homology**
    - **2.4** Supplementary Tables - Sponge genomes
        - ST 2.1 **| Cladorhizid sponge and *Ephydatia muelleri* chromosome numbering**

3. **Genome sequencing, assembly, and annotation - Unicellular Outgroup Species**
    - **3.1** Methods - Unicellular Outgroup Species
        - **3.1.1** Hi-C library preparation
        - **3.1.2** Genome scaffolding and annotation
        - **3.1.3** Three assembly versions of *Capsaspora owczarzaki*
        - **3.1.4** Putative centromere locations
    - **3.2** Results and Discussion - Unicellular Outgroup Species
        - **3.2.1** Hi-C sequencing summary
        - **3.2.2** Summary of genome assemblies
        - **3.2.3** Putative centromere locations
        - ST 3.1 **| Estimated centromere positions in unicellular species**

4. Chromosomal tectonic events and their phylogenetic implications
4.1 Introduction
4.2 Assumptions
SF 4.1 **| The assumptions of the phylogenetic methodology used in this manuscript**
4.3 Oxford dot plots allow identification of synteny between two genomes
SF 4.2 **| Example Oxford dot plot between two species, with significance test**
4.4 Ancestral Linkage Group identification in three or more species
ST 4.1 **| Hypothetical orthologs and chromosome coordinates from four species**
ST 4.2 **| More hypothetical orthologs and chromosome coordinates from four species**
4.5 Phylogenetic implications of chromosome tectonic events identified with 4-species ALGs
4.5.1 Fusion with mixing in two non-outgroup species
ST 4.3 **| ALG table of a putative fusion-with-mixing event**
SF 4.3 **| ALGs involved in a fusion-with-mixing event**
4.5.2 ALGs with unique combinations of chromosomes
ST 4.4 **| A single ALG with a unique combination of chromosomes**
SF 4.4 **| Phylogenetic interpretations of ALGs with unique chromosome combinations**
4.5.3 One fission event in the outgroup / One fusion in the ancestor of other species
ST 4.5 **| One fission event in the outgroup / One fusion in the ancestor of other species**
SF 4.5 **| An ambiguous scenario of an ancestral fusion or derived fusion**
4.5.4 Two ALGs fused in a single ingroup species
ST 4.6 **| ALGs of a putative derived fusion-with-mixing**
SF 4.6 **| Phylogenetic interpretations of two ALGs mixed only in one species**
4.5.5 Two ALGs appear by fission in one ingroup species
ST 4.7 **| A putative fission event in the ancestor of one species**
SF 4.7 **| Phylogenetic interpretations of two ALGs with one unfused species**
4.5.6 One fission event in the ingroup lineage
ST 4.8 **| Putative ancestral fission event in the ancestor of two species**
SF 4.8 **| Phylogenetic interpretations of an apparent derived fission**
4.5.7 Software implementation of synteny analyses
ST 4.9 **| Fusion in the branch leading to the outgroup, or an ancestral fission**
SF 4.9 **| Phylogenetic interpretations of two ALGs fused in the outgroup**

5. ODP: software to perform macrosynteny analyses
5.1 Introduction
5.2 Methods
5.2.1 Software implementation of synteny analyses
5.2.2 Genome selection and data preparation
5.2.3 Two-way and n-way reciprocal best blastp searches
5.2.4 Plotting synteny between two species
5.2.5 Identifying reciprocal best hits in additional species using HMMs
5.2.6 Identifying instances of fusion-with-mixing in *odp*
5.2.7 Genome shuffling simulations for hypothesis testing
SF 5.1 **| The three hypothesis tests of odp_genome_rearrangement_simulation**

6. **Validating the methodology and ODP software with other clades**
6.1 Introduction
6.2 Methods
6.3 Results and Discussion
6.3.1 Recovery of bilaterian fusion-with-mixing synapomorphies
SF 6.1 **| Automated ALG analysis recovers bilaterian synapomorphies**
6.3.2 Recovery of cnidarian fusion-with-mixing synapomorphies
SF 6.2 **| Automated ALG analysis recovers cnidarian synapomorphies**

7. **Macrosynteny analyses of animals and their close unicellular relatives**
7.1 Introduction
7.2 Results and Discussion
7.2.1 Conservation of ctenophore karyotype
7.2.2 Conservation of the demosponge karyotype
7.2.3 Conservation of karyotype between Porifera, Cnidaria, and Bilateria
7.2.4 Rearranged karyotype in ctenophores relative to other animals
7.2.5 The derived karyotype of hexactinellid sponges
7.2.6 A1a_x and A1a_y exist on separate chromosomes in lyssacinosid glass sponges
7.2.7 Karyotype of the choanoflagellate *Salpingoeca* compared to animals
7.2.8 Karyotype of the filasterean amoeba *Capsaspora* compared to animals
7.2.9 Karyotype of the ichthyosporean *Creolimax* compared to animals
7.2.10 Comparison of the karyotypes of the unicellular outgroup species
7.3 Supplementary Figures
SF 7.1 **| *Capsaspora*-ALG synteny is limited to single *Capsaspora* chromosome arms**
SF 7.2 **| The ichthyosporean Creolimax does not exhibit conserved macrosynteny with metazoans**
SF 7.3 **| Oxford dot plots comparing unicellular species**
7.4 Supplementary Tables
ST 7.1 **| Genomes used in macrosynteny analyses**
ST 7.2 **| *Hormiphora* chromosomes and ALG arithmetic**

8. **Identification of gene groups linked since the ancestor of the Filozoa**
8.1 Introduction
8.2 Results and Discussion
8.2.1 COW-HCA-RES-EMU gene linkage groups
8.2.2 SRO-HCA-RES-EMU gene linkage groups
8.2.3 CFR-HCA-RES-EMU gene linkage groups
8.2.4 Merging the (COW, SRO, CFR)-HCA-RES-EMU analyses
8.2.5 Alternate *Capsaspora* genome assemblies do not change linkage group results

9. **Extension of gene linkage groups to other metazoan species**
9.1 Introduction
9.2 Methods
9.2.1 HMM search of 291 orthologs in additional species
9.3 Results and Discussion

9.3.1 Conservation of ancestral linkage groups in additional species
9.3.2 Conservation of ancestral linkage groups in *Trichoplax*

10. OrthoFinder analysis recovers support for the ctenophore-sister hypothesis
10.1 Introduction
10.2 Methods
10.2.1 OrthoFinder
10.2.2 Species quartets from OrthoFinder
10.3 Results and Discussion
10.3.1 Species quartet analyses support the ctenophore-sister hypothesis
10.3.2 Overlap in gene content between rbh and OrthoFinder analyses

11. Detecting conserved macrosynteny between highly rearranged genomes
11.1 Introduction
11.2 Methods
11.2.1 Interpreting two-species comparisons in a muti-species context
11.2.2 Ortholog data structure
11.2.3 Conservation Score
11.2.4 Significance Testing
11.3 Results and Discussion
11.3.1 False positives cutoff in the ortholog-ortholog conservation score
ST 11.1 **| CFR-HCA ortholog-ortholog conservation score significant groupings**
11.3.2 False positives in the ortholog network conservation score
ST 11.2 | **CFR-HCA ortholog network conservation score significant groupings**
11.3.3 Corroboration with Fisher's exact test results with R. esculentum
SF 11.1 **| COW-RES unfiltered Fisher's Exact Test Results**
ST 11.3 | **COW-RES conservation scores significant groupings**
11.3.4 COW-HCA conservation scores
ST 11.4 | **COW-HCA ortholog-ortholog t ≥ 0.5 conservation score table**
ST 11.5 | **COW-HCA ortholog network t ≥ 0.35 conservation score table**
11.3.5 SRO-HCA conservation scores
ST 11.6 | **SRO-HCA ortholog-ortholog t ≥ 0.5 conservation score table**
ST 11.7 | **SRO-HCA ortholog network t ≥ 0.35 conservation score table**
11.4 Discussion

12. GO enrichment analysis of ALGs conserved in Filozoans
12.1 Introduction
12.2 Methods
12.2 Results and Discussion

13. Entropy of gene mixing analysis
13.1 Methods
13.1.1 Visualizing gene mixing of two groups of genes on single chromosomes
13.1.2 Quantifying the degree of mixing of two groups of genes on single chromosomes
13.2 Results and Discussion
13.2.1 Degree of mixing between phylogenetically informative groups of linkage groups

13.2.2 Search of EMU-RES-HCA to expand metazoan gene linkages

14. Analyzing chromosomal tectonic events in a Bayesian phylogenetic framework
14.1 Introduction
14.2 Methods
14.2.1 Constructing a two-state character matrix of chromosome fusion states
14.2.2 Bayesian phylogenetic analysis
14.3 Results and Discussion
SF 14.1 **| Bayesian analysis of chromosome fusion states recovers ctenophore-sister**

15. Null hypothesis testing of the ctenophore-sister topology
15.1 Introduction
15.2 Methods
15.2.1 Design of genome shuffling simulation
15.3 Results and Discussion
15.3.1 Genome shuffling simulation results

16. Supplementary Information References

1 Genome sequencing, assembly, and annotation - *Bolinopsis microptera*:

1.1 Methods - *Bolinopsis microptera*

1.1.1 Sample Collection - *Bolinopsis microptera*

Samples of *Bolinopsis microptera* were collected on May 24th, 2015 in the Monterey Bay, California (36.63°N, 121.90°W) with jars from the surface waters, with permission under the State of California Department of Fish and Wildlife collecting permit SC-2026 to the Monterey Bay Aquarium. A community culture was founded with 20 *B. microptera* individuals in pseudokreisel tanks and diffusion tubes in 12°C seawater at the Monterey Bay Aquarium in Monterey, California. The culture was reared according to the published protocol[110] for three generations, and an F3 adult, called Bmic1, was selected on November 18th, 2019 for DNA sequencing for genome assembly and annotation (Pictured in **Fig. 1**). Four other F3 adults were placed into a spawning tank and spawned according to the published protocol[110]. Fertilized eggs were collected 18 hours post-spawning for RNA sequencing.

1.1.2 DNA and RNA sequencing - *Bolinopsis microptera*

High molecular weight (HMW) DNA was isolated from *B. microptera* individual Bmic1 by lysing tissue in CTAB buffer[111], then purifying with a chloroform, phenol:chloroform, chloroform, ethanol precipitation protocol[112]. The HMW DNA was sent to Brigham Young University DNA Sequencing Center, where one PacBio CLR library was constructed and sequenced on two Sequel II 15-hour SMRT cells. This yielded 2 Gigabases (Gb) of data in 7,053,509 reads. The mean read length was 7.4 kilobases (kb), and the read N50 was 11 kb.

Remaining HMW DNA was used to construct two NEBNext Ultra II FS whole genome shotgun (WGS) libraries for Illumina sequencing. The libraries were sequenced to a depth of 51.9 million read pairs (61x coverage) at MedGenome, Inc on an Illumina HiSeq X 2x150 run.

Total RNA was isolated from approximately 10 mg of 18-hour-post-fertilization *B. microptera* embryos using a Trizol RNA isolation protocol[113]. The RNA was converted to a PacBio Iso-Seq library and sequenced on one Sequel II SMRT cell at the Brigham Young University DNA Sequencing center. This yielded 2,691,284 CCS Iso-Seq reads, with a mean read length of 3.0 kb and a read N50 of 3.1 kb. Primers and barcodes were removed with Pacific Biosciences lima v2.2.0 (github.com/PacificBiosciences/barcoding), and 2,623,057 full-length non-chimeric (FLNC) reads were generated with Pacific Biosciences isoseq3 v3.4.0 (github.com/PacificBiosciences/IsoSeq). See **Supplementary Table 1.1** for a sequencing data summary, and **Supplementary Data 1** for sequencing library details.

1.1.3 Hi-C library preparation - *Bolinopsis microptera*

We generated one DpnII-based Hi-C library, one MluCI-based Hi-C library, and one FatI-based Hi-C library for *B. microptera* individual Bmic1 using a previously-described Hi-C library preparation method based on binding chromatin to magnetic solid phase reversible separation (SPRI) beads[64]. This same protocol was used to construct the sponge Hi-C libraries (**Supplementary Information 2.1.3**) and unicellular organism Hi-C libraries (**Supplementary Information 3.1.1**). The restriction site linker was contained in 22% of the DpnII Hi-C library reads, in 14% of the MluCI Hi-C library reads, and in 2% of the FatI Hi-C library reads. These libraries were sequences to a depth of 300 million read pairs (DpnII), 168 million (MulCI), and 190 million read pairs (FatI). Sequencing was performed on a HiSeq Nova 6000

2x150 run at MedGenome, Inc in Foster City, California. See **Supplementary Table 1.1** for a sequencing data summary, and **Supplementary Data 1** for sequencing library details.

1.1.4 Genome assembly - *Bolinopsis microptera*

Our goal in assembling the *Bolinopsis microptera* genome was to represent each pair of orthologous chromosomes by a single sequence, rather than creating a haplotype-resolved assembly.

The Pacific Biosciences CLR subreads for *B. microptera* individual Bmic1 were assembled into contigs using the wtdbg2 assembler v2.4[73] with parameters `-g 270m -X 50 -p 17 -k 0 -e 3 -A -S 2 -s 0.05 -L 5000.` Dovetail Genomics HiRise vAug2019 was used to scaffold the genome using only the Hi-C libraries[65]. Hi-C heatmaps were prepared as previously described[40] , and scaffold misjoins identified from the Hi-C maps were split into two new scaffolds by breaking at the nearest gap. The Pacific Biosciences CLR subreads were then used to gapfill the assembly using TGS-Gapcloser v1.1.1[78]. Diamond v0.9.24[114] and Blobtools v1.0[82] were used to remove non-metazoan scaffolds. The scaffolds were then broken into contigs. The genome was re-scaffolded using HiRise vAug2019, gapclosed once more, then polished with the Illumina WGS reads using pilon v1.23[80]. Another Hi-C heatmap was generated, and the assembly was predominantly composed of 13 chromosome-scale scaffolds.

Duplicate haplotigs were removed with Purge Haplotigs v1.0.4[79] using parameters `purge_haplotigs cov -l 50 -m 175 -h 600 -j 70 -s 80` and `purge_haplotigs purge -a 30`. We then ran `purge_haplotigs clip` to remove overlapping contig ends. Final genome assembly statistics can be found in Supplementary Table 2.

1.1.5 Genome Annotation - *Bolinopsis microptera*

The *Bolinopsis microptera* genome assembly was annotated using a combination of evidence sources. The Iso-Seq reads collected in this study were aligned to the genome using minimap2[84]. Previously published *Bolinopsis* Illumina RNA-seq reads (SRX250327, SRX263022, and SRX3215100) were aligned to the genome using STAR v2.7.1a. We assembled ctenophore transcriptomes using Trinity v2.5.1[95] with the parameter `--SS_lib_type RF` using ctenophore Illumina RNA-seq data available on NCBI SRA (**Supplementary Table 1.2**). Proteins were predicted from these transcriptomes using TransDecoder v5.5 (github.com/TransDecoder). Protein hints were generated in the *B. microptera* genome using the proteins from other ctenophore transcriptomes using ProtHint v2.6.0[91]. The Iso-Seq read alignments, Illumina RNA-seq read alignments, and the protein hints were used in BRAKER v2.14[89] to generate annotation files. The final annotation contained 77957 protein sequences, and the BUSCO v5[96] Eukaryota complete score was 85%. This score is not as high as the protein set from the genome assembly of *H. californensis*[40], but is higher than protein sets from the genomes of *M. leidyi* and *P. bachei*. See **Supplementary Table 1.3** for more details about the annotation information.

1.2 Results and Discussion - *Bolinopsis microptera*

1.2.1 Genome assembly summary - *Bolinopsis microptera*

The ctenophore *Bolinopsis microptera* genome assembly was 265.4 Mbp in 246 scaffolds, and 97.2% of the bases were in 13 chromosome-scale scaffolds. The chromosome-scale scaffolds of the unicellular species' genomes contain 97.6% or more of the proteins present in the original genome assemblies. The *B. microptera* genome annotation assessment using Benchmarking Universal Single-Copy Orthologs (BUSCO)[96] indicates that 85.1% of the Eukaryota gene set is present and complete. The genome has a lower BUSCO score than the manually curated *H. californensis* annotation, but a higher BUSCO score than both the *P. bachei* and *M. leidyi* genomes.

1.3 Supplementary Tables - *Bolinopsis microptera*

Species	Data Type	Total Gb	Number of read pairs	% Linker	Read Depth Coverage
Salpingoeca rosetta ATCC PRA-366	Hi-C: DpnII	12.0 Gb	40.1 M	44.1 %	217 x
	Hi-C: MluCI	15.9 Gb	53.1 M	17.3 %	288 x
Capsaspora owczarzaki ATCC 30864	Hi-C: DpnII	22.6 Gb	75.4 M	40.2 %	821 x
	Hi-C: MluCI	6.3 Gb	20.8 M	16.7 %	226 x
Creolimax fragrantissima ATCC PRA-284	Hi-C: DpnII	8.1 Gb	27.1 M	40.3 %	181 x
	Hi-C: MluCI	15.3 Gb	50.9 M	10.3 %	340 x
Bolinopsis microptera	Hi-C: DpnII	90.5 Gb	301.7 M	21.9 %	355 x
	Hi-C: MluCI	50.5 Gb	168.2 M	14.0 %	198 x
	Hi-C: FatI	57.1 Gb	190.4 M	1.7 %	224 x
	Chicago: DpnII	3.9 Gb	13.1 M	9.11 %	26 x
	Chicago: MluCI	4.4 Gb	14.6 M	3.24 %	29 x
	Chicago: FatI	3.9 Gb	11.9 M	0.5 %	24 x
	Illumina WGS	15.59 Gb	51.9 M	N/A	104 x
	CLR WGS	52.0 Gb	7.0 M	N/A	204 x
	Iso-Seq	8.1 Gb	2.7 M	N/A	N/A
Cladorhizid sponge	Hi-C: DpnII	81 Gb	270 M	24.5 %	101 x
	Hi-C: MluCI	76 Gb	255 M	12.4 %	95 x
	HiFi WGS	77.8 Gb	5.58 M	N/A	70 x
	Iso-Seq	7.5 Gb	3.36 M	N/A	N/A

Supplementary Table 1.1 | **Sequencing libraries for unicellular animal outgroup species.**

Species	Read Accession	Number of Read Pairs (M)	Citation
Beroe abyssicola	SRR777787	22.7	[5]
Beroe forskalii	SRR6074515	39.7	[8]
Beroe ovata	SRR6074516	7.7	[8]
Beroe sp. UF-2017	SRR5892577	22.0	[8]
Bolinopsis ashleyi	SRR5892570	23.9	[8]
Bolinopsis infundibulum	SRR6074521	52.3	[8]
Bolinopsis infundibulum	SRR786491	21.6	[5]
Bolinopsis microptera	SRR3048531	25.1	[24,25]
Bolinopsis mikado	DRR189212	15.7	NA
Coeloplana astericola	SRR786490	20.8	[5]
Coeloplana cf. *meteoris*	SRR3407215	73.6	[8]
Ctenophora sp. LM-2017	SRR6074512	23.2	[8]
Cydippida sp. LM-2017	SRR6074511	43.9	[8]
Dryodora glandiformis	SRR777788	20.6	[5]
Euplokamis dunlapae	SRR777663	34.1	[5]
Eurhamphaea vexilligera	SRR6074510	22.3	[8]
Hormiphora palmata	SRR6074513	45.5	[8]
Hormiphora sp.	SRR1992642	32.3	[92]
Lampea pancerina	SRR3407163	28.7	[8]
Lampea sp.	SRR9162937	48.2	[93]
Mertensiidae sp.	SRR786492	23.7	[5]
Mnemiopsis leidyi	ERR2752243	26.9	[94]
Mnemiopsis leidyi	ERR2752250	27.8	[94]
Mnemiopsis leidyi	SRR6074509	58.2	[8]
Ocyropsis crystallina	SRR6074507	53.1	[8]
Ocyropsis crystallina	SRR6074508	42.4	[8]
Pleurobrachia pileus	SRR6074514	14.5	[8]
Pleurobrachia pileus	SRR789901	25.3	[5]
Pleurobrachia sp. UF-2017	SRR5892573	19.7	[8]
Pleurobrachia sp. C LM-2017	SRR6074517	19.8	[8]
Pukia falcata	SRR5892572	25.8	[8]
Vallicula multiformis	SRR3407164	34.9	[8]
Vallicula multiformis	SRR786489	24.5	[5]
Vampyroctena delmarvensis	SRR9162936	60.4	[93]

Supplementary Table 1.2 **| Ctenophore SRAs used for annotating the *Bolinopsis* genome.**

Sample	BUSCO string	Number of transcripts (protein-coding)	Transcript N50 (AA)
B. microptera AUGUSTUS	C:84.3%[S:75.3%,D:9%],F:8.6%,M:7.1%,n:255	73883	511
B. microptera GeneMark-ETP	C:84.3%[S:75.3%,D:9%],F:8.6%,M:7.1%,n:255	53544	558
B. microptera BRAKER	C:85.1%[S:70.2%,D:14.9%],F:7.8%,M:7.1%,n:255	77957	517
H. californensis genome	C:91.0%[S:82.4%,D:8.6%],F:4.3%,M:4.7%,n:255	19693	679
P. bachei genome	C:45.9%[S:45.9%,D:0%],F:18.8%,M:35.3%,n:255	18990	552
M. leidyi genome	C:83.6%[S:82.4%,D:1.2%],F:8.2%,M:8.2%,n:255	16548	601

Supplementary Table 1.3 | **Ctenophore genome annotation statistics.** BUSCO scores represent complete (C), fragmented (F), and missing (M) Eukaryota genes; genes are annotated as single copy (S) or duplicated (D). Transcripts include alternate isoforms. There were 14238 protein-coding gene loci found during manual annotation of the *H. californensis*[40] genome.

Species	Genome Size (Mb)	Number of Chr-scale Scaffolds	% of Bases in Chr-scale Scaffolds	Total Scaffolds	Scaffold N50 (Mb)	Number Contigs	Contig N50 (Mb)
Capsaspora owczarzaki[43]	27.97 Mb	13	75.64 %	84	1.6	625	0.1289
Capsaspora owczarzaki[70]	27.77 Mb	14	82.84 %	55	2.0	307	0.2622
Capsaspora owczarzaki (this publication)	27.40 Mb	16	100 %	16	1.8	328	0.2463
Creolimax fragrantissima[44]	44.8 Mb	-	-	83	1.6	536	0.1713
Creolimax fragrantissima (this publication)	43.86 Mb	27	99.15 %	62	1.6	514	0.1726
Salpingoeca rosetta[42]	55.44 Mb	-	-	154	1.5	3086	0.0348
Salpingoeca rosetta (this publication)	55.45 Mb	36	99.72 %	68	1.7	3090	0.0348
Bolinopsis microptera	265.47 Mb	13	97.23 %	346	20.8	557	3.1
Cladorhizid sp. hap A	857.6 Mb	18	94.3 %	326	44.6	1465	1.3
Cladorhizid sp. hap B	940.5 Mb	18	83.4 %	402	41	1314	1.5
Hexactinellid sp. hap A	112.4 Mb	18	100%	18	5.9	33	5.4
Hexactinellid sp. hap B	195.7 Mb	18	73.4%	32	6.5	55	5.5

Supplementary Table 1.4 | **Genome assembly statistics.** This table includes statistics for both the input (i.e., pre-Hi-C) assemblies, and final assemblies, of the three unicellular species, the ctenophore genome, and the two sponge genomes.

2 Genome sequencing, assembly, and annotation - Sponge Genomes:

2.1 Methods - Sponges

2.1.1 Sample Collection - Sponges

One individual of an undescribed hexactinellid sponge was collected on June 1st, 2021, in the Monterey Bay, California (34.57°N, 122.56°W) from the seafloor at 3,852 meters depth using the MBARI *ROV Doc Ricketts* aboard the *R/V Western Flyer*. On the following day one individual of an undescribed bioluminescent cladorhizid sponge was collected from a nearby site (35.49°N, 124°W) from the seafloor at 3,975 meters depth. The collection temperature of both samples was 1.5°C. The cladorhizid sample was consistent in morphology and locale with previously reported bioluminescent, carnivorous, cladorhizid sponges[36]. Upon retrieval from the ROV, the samples were washed gently with 1°C filtered seawater to remove debris. The cladorhizid sponge was confirmed to be bioluminescent with a prodding assay and filmed with a Sony α7s III. Then, both samples were flash-frozen in liquid nitrogen. The wet weight of the cladorhizid sample was approximately 2 grams, and the wet weight of the hexactinellid sponge was approximately 49 grams. The samples were collected with the State of California Department of Fish and Wildlife collecting permit SC-4029 granted to the Haddock Laboratory at the Monterey Bay Aquarium Research Institute. The cladorhizid and hexactinellid individuals sequenced in this study is shown in **Extended Data Figure 1**.

2.1.2 DNA and RNA sequencing - Sponges

The sponges were sent to Brigham Young University DNA Sequencing Center, where they prepared one PacBio HiFi library per species. For each of the species, HMW DNA for the HiFi WGS library was extracted by first powderizing 500mg of sample under liquid nitrogen, then digesting the tissue in 5mL of 30mM Tris, 10mM EDTA, 1% SDS, 20µL/mL proteinase K solution at pH 8.0 at 53°C for 4 hours. The samples were spun down at 500 rcf at room temperature for 5 minutes to pellet debris. The supernatants were added to new tubes with 10mL of 96% EtOH, and then incubated at -20°C for one hour. The cold mixtures were then centrifuged at 15k rcf at 4°C for 10 minutes. The supernatants were removed, then washed with 80% EtOH. At this stage G2 buffer was added from the Qiagen Genomic-tip 20/G kit. The G2 solution and pellet mixtures were allowed to sit overnight in a refrigerator at 4°C. These were then used in the Qiagen Genomic-tip 20/G protocol. The resulting HMW DNA samples were used in the PacBio Hifi library preps.

The PacBio SMRTbell Express Template Prep Kit 2.0 + Enzyme Clean Up Kit 2.0 + Sequencing Primer v5 Bundle (PN: 102-088-900) was used to prepare the HiFi WGS libraries. The library of the bioluminescent cladorhizid sponge was sequenced on three Sequel II SMRT Cells (8M) on the 15-hour movie mode. The library of the hexactinellid sponge was sequenced in two Sequel II SMRT Cells (8M) on the 15-hour movie mode. CCS reads were called using the software bundled with PacBio SMRTtools release 10.1.0.119588. These 3 SMRT cells of the library from the cladorhizid sponge yielded 77.8 Gigabases (Gb) of data in 7,053,509 reads. The mean read length was 13.9 kb. The 2 SMRT cells of the library from the hexactinellid sponge yielded 42.8 Gb of reads in 2,842,892 reads.

Total RNA was isolated from 25 mg of frozen cladorhizid sponge tissue using TRIzol reagent[113] at Brigham Young University. An Iso-Seq library was then prepared at Brigham Young University with the Iso-Seq Express Library Preparation Using SMRTbell Express Template Prep Kit 2.0 kit. This library was sequenced on a single Sequel II SMRT cell, and yielded 3.36 million CCS reads with a mean read length of 2.5 kb. Like the *Bolinopsis* sample, the primers and barcodes were removed with lima v2.2.0, and

FLNC reads were generated with isoseq3 v3.4.0. There were 2.13 M reads remaining after barcode removal and FLNC processing (**Supplementary Table 1.1, Supplementary Data 1**).

2.1.3 Hi-C library preparation - Sponges

For both the cladorhizid sponge and the hexactinellid sponge, we generated one DpnII-based Hi-C library and one MluCI-based Hi-C library using the same protocol used for the *Bolinopsis* Hi-C libraries (**Supplementary Information 1.1.3**) and for the unicellular outgroup-to-animal species' Hi-C libraries (**Supplementary Information 3.1.1**)[64]. In the cladorhizid libraries, the restriction site linker was present in 24.5% of the DpnII Hi-C library reads, and in 12.4% of the MluCI Hi-C library reads. In the hexactinellid libraries, the restriction site linker was present in 37.57% of the DpnII Hi-C library reads, and in 45.2% of the MluCI Hi-C library reads. The cladorhizid sponge Hi-C libraries were sequenced to a depth of 120x genome coverage, and the hexactinellid Hi-C libraries were sequenced to 235x genome coverage. Sequencing was performed on two separate runs of a HiSeq X 2x150 run at MedGenome, Inc in Foster City, California. See **Supplementary Table 1.1** for a sequencing summary and **Supplementary Data 1** for sequencing details.

2.1.4 Genome Assembly - Sponges

Before assembly, the genome sizes of the sponge species were estimated by counting 19-mers of the PacBio HiFi reads with jellyfish v2.2.10[71] then using the resulting spectrum in GenomeScope 2[72].

The cladorhizid and hexactinellid sponge CCS reads and Hi-C reads were assembled using hifiasm v0.16.1-r375[74] with the additional parameter –n-weight 5, to increase the number of Hi-C links reweighting steps from 3 times to 5 times. The Hi-C reads were included in the assembly stage to ensure phase consistency within chromosomes. The Hi-C libraries were used to scaffold each resulting hifiasm haplotype fasta file with Dovetail Genomics HiRise vAug2019[65]. Diamond v0.9.24[114] and blobtools v1.0[82] were used to identify scaffolds that were non-metazoan in origin. These scaffolds were removed from the assembly and placed in their own metagenome file. CCS reads were mapped to the resulting assemblies with minimap2 v2.23-r1111, and Hi-C reads were mapped to the assemblies using bwa mem v0.7.17[75]. D-Genies v1.4.0[83] was used with minimap2 v2.23[84] to produce dot plots to find orthologous chromosomes between haplotypes A and B.

PretextView v0.2.4 (https://github.com/wtsi-hpag/PretextView/releases), HiGlass v1.10.0[67], Juicebox Assembly Tools github commit 46c7ed1[68], the Juicebox visualization system v1.11.08[115], and artisanal (https://bitbucket.org/bredeson/artisanal) were used to manually curate the assembly. For the manual curation process, contigs with high Hi-C contacts to chromosome-scale scaffolds were placed in an order and orientation within the chromosome-scale scaffold to minimize off-diagonal contacts. Repetitive sequences that had Hi-C contacts with the chromosome-scale scaffolds were included in the chromosome-scale scaffolds rather than left as standalone sequences.

2.1.5 Genome Annotation - Sponges

Since our goal was chromosome level comparisons, our annotation of the cladorhizid sponge genome in this study targeted the discovery of orthologous protein-coding genes in the *Ephydatia* chromosomes. Toward this goal, we mapped the location of the *Ephydatia* proteins to the cladorhizid sponge scaffolds. To do this, we used the 33,096 proteins from the 25 *Ephydatia* chromosome-scale scaffolds as a query, and the 18 cladorhizid sponge chromosome-scale scaffolds as a database, in a tblastn v2.10.0+ search[85]. For each *Ephydatia* protein query, we selected the best hit as the putative location of the orthologous gene in the cladorhizid sponge genome. A GFF file was generated from these results for downstream

macrosynteny analyses. The final GFF contained 29,648 proteins from the *Ephydatia* genome mapped to the cladorhizid sponge genome.

We used a protein-mapping approach to annotate both haplotypes of the hexactinellid sponge genome. We used Trinity v2.5.1[95] to assemble the transcriptomes of the hexactinellid sponges *Rosella fibulata* (SRR1915835)[87], *Hyalonema populiferum* (SRR1916923)[87], and *Sympagella nux* (SRR1916581)[87]. We also downloaded the proteins of *Oopsacas minuta* from NCBI[86]. These four sets of proteins were mapped to the hexactinellid genome assembly using miniprot[88] commit df41f09 (https://github.com/lh3/miniprot). Proteins that have the same start and or end coordinate were made non-redundant, keeping the protein with the highest number of identical amino acids with the hexactinellid reference genome. The final GFF of haplotype A of the hexactinellid sponge contained 57,255 protein-coding genes, and the final GFF of haplotype B of the hexactinellid sponge contained 68,858 protein-coding genes.

2.1.6 Species verification - Sponges

For further evidence clarifying the taxonomy of the cladorhizid sponge, we mapped the HiFi reads of one PacBio SMRT cell to the mitochondrial genome fragment of the previously reported Clado1 sample (MN418897)[36]. To do this we used minimap2 v2.23[84] with options `-ax map-hifi` and retained only mapped reads with mapping quality 60. We then inspected the bam file with IGV v2.3[116] to identify single nucleotide polymorphisms (SNPs).

To identify the species of hexactinellid sponge sequenced for this study, we aligned the HiFi reads to a database of Hexactinellid COI sequences, extracted the reads with a map quality score of 60, and assembled them with hifiasm v0.16.1-r375[74]. The resulting contig was searched against the nt database with blastn. We compared the hexactinellid nuclear genome against the nuclear genome of *Oopsacas minuta* with the odp software to check that they were colinear, or at least shared macrosynteny.

2.2 Results and Discussion - Sponges

2.2.1 Species identification - Sponges

We first verified that the sponge individual that we sequenced was of the same species as the bioluminescent, carnivorous, cladorhizid sponges previously reported[36]. First, the morphology of the sponge that we collected (**Fig. 1, Extended Data Fig. 1c,d**) matches the morphology and collection location of the previously reported sponge individuals. For further evidence clarifying the taxonomy of the individual that we sequenced, we mapped the HiFi reads of one PacBio SMRT cell to the mitochondrial genome fragment of the previously reported Clado1 sample (MN418897)[36] (See **Extended Data Figure 1e**). From the bam file we identified 31 SNPs out of 3727 positions (0.8% of bases) between the individual sequenced in this paper, and the other individual from sequence MN418897. A divergence of 0.8% is within the range of nucleotide diversity of mitochondria found within populations of panmictic marine species[117].

The cladirhizid species closely resembles the recently described species, *Bathytentacular moniqueae*[118], discovered off the southern coast of Australia. We compared the 28S sequences from *B. moniqueae* and the cladorhizid sponge collected off the coast of California and found that 99.4% of the 374 nucleotides were identical. It is unclear from morphology and other molecular sequences whether these are the same species, and future studies on these sponges may resolve their taxonomy.

The mitochondrial sequence of the hexactinellid sponge was 21,191 basepairs long. The blastn hit with the highest percent identity was a 95.73% identity hit to the sponge *Dictyaulus kexueae* (Porifera; Hexactinellida; Hexasterophora; Lyssacinosida; Euplectellidae; *Dictyaulus*) mitochondrial large ribosomal

subunit RNA. The second highest-identity hit to a species that was not *Dictyaulus* was a 95.09% identity hit to the mitochondrial large ribosomal subunit RNA from the species *Rhabdopectella tintinnus* (Porifera; Hexactinellida; Hexasterophora; Lyssacinosida; Euplectellidae; Rhabdopectella). The lowest common shared taxonomic unit for these hits is the family Euplectellidae. This is the same order, Lyssacinosida, as *Oopsacas minuta*, for which a sub-chromosomal scale genome assembly was recently released[86]. The Oxford dot plot of the hexactinellid genome presented in this manuscript compared to the genome of *Oopsacas minuta* revealed that the two genomes are highly colinear (**Supplementary Figure 2.1**). This suggests that the assembly quality of the chromosome-scale genome presented here is high, that its protein-mapping annotation captures much of the protein-coding sequences of the genome, and likewise that the *Oopsacas minuta* genome assembly of Santini et al. (2022)[86] is nearly chromosome-scale.

2.2.2 Cladorhizid sponge genome - summary

Given the high coverage of PacBio HiFi reads in this dataset, our goal for the Cladorhizid sponge genome was to generate a haplotype-resolved assembly containing chromosome-scale scaffolds for both the maternal and paternal haplotypes.

Both haplotypes of the cladorhizid sponge genome contained 18 large scaffolds. Hi-C maps of these 18 large scaffolds were consistent with the strong intra-scaffold Hi-C pattern found in karyotype-backed chromosome-scale genome assemblies (**Extended Data Fig. 1g,h**). The next several largest scaffolds (< 6 Mbp) after the smallest apparent cladorhizid sponge chromosome (29 Mbp) did not share Hi-C connections with the putative sponge chromosomal scaffolds. Instead, many scaffolds shorter than 6 Mbp resembled the Hi-C pattern of prokaryotic chromosomes that co-assembled in the *Bolinopsis* ctenophore dataset. We verified that many of these small scaffolds were of prokaryotic origin, and separated them into a separate metagenome-containing fasta file[82].

Chromosome numbering for the chromosome-scale cladorhizid sponge scaffolds was determined by comparing the assemblies to the *Ephydatia muelleri* assembly using a dot plot in D-Genies v1.4.0[83]. Where possible, we gave the cladorhizid sponge the same chromosome number designation as the 1:1 homologous chromosome in the *Ephydatia* genome. While the cladorhizid sponge chromosomes and *Ephydatia* each have 10 chromosomes that share 1:1 orthology, the cladorhizid sponge genome only appears to contain 18 chromosomes. The 8 remaining chromosomes appear to be fusions of two or three *Ephydatia* chromosome fragments. Each of the BCnS ALGs appeared on single chromosomes in the cladorhizid sponge genome, with the exception of ALG_H (**Extended Data Fig. 2**). We later found that the split of BCnS ALG_H in the cladorhizid sponge is not shared with other sponges, and therefore is likely an autapomorphy (**Supplementary Information 7.2.2, Extended Data Fig. 3**). Chromosome-scale scaffolds were also reverse-complemented to match the orientation of the *Ephydatia muelleri* assembly. See **Supplementary Table 2.1** for a simple tabular format listing homologous chromosomes between *Ephydatia* and the cladorhizid sponge, and see **Figure 1d** for a ribbon diagram visualization of their chromosome homology using gene index coordinates.

The genome size of the cladorhizid sponge estimated from k-mers was 1.116 billion basepairs (**Extended Data Fig. 1f**). Haplotype A of the cladorhizid sponge genome assembly was 912.8 Mbp in 1170 scaffolds (N50 is 38.4 Mbp), and 77.4% of the bases were in 18 chromosome-scale scaffolds. Haplotype B of the assembly was 960.0 Mbp in 653 scaffolds (N50 is 37.1 Mbp), and 74.4 % of the assembly was in 18 chromosome-scale scaffolds (**Supplementary Table 1.4**).

2.2.3 Hexactinellid sponge genome - summary

Our assembly strategy for the hexactinellid genome was the same as that of the cladorhizid genome. We pursued a genome assembly in which the maternal and paternal chromosomes were independently assembled using the 291x genome coverage of PacBio HiFi reads and the 166x coverage of Hi-C data.

The glass sponge genome assembly haplotype A and B fasta files contained between 18-32 chromosome-scale scaffolds after removing small scaffolds that were duplicates of regions in larger scaffolds, and further manual curation of the assembly based on Hi-C maps. Only 18 scaffolds of each of these assemblies had a high rate of proteins mapped from other sponge species, and only those 18 scaffolds in each haplotype had significant orthology to the scaffolds of the *Oopsacas minuta* (**Supplementary Fig. 2.1**) and *Ephydatia muelleri* genomes. Both the haplotype A and haplotype B fasta files contain one chromosome-scale scaffold for each of these 18 sequences that appear to be homologous to *Oopsacas* scaffolds. Many of these chromosome-scale scaffolds were single contigs (no runs of N characters in the fasta file), and the frequency of mapped proteins from closely-related species was highest in the distal portions of the chromosomes, rather than randomly distributed across the entire lengths.

There were an additional 14 scaffolds between the haplotype A and B assemblies. These 14 scaffolds had interchromosomal Hi-C signal strength and character that was consistent with being derived from the same nuclei as the 18 conserved chromosome-scale scaffolds conserved with other sponge species. In other words, the Hi-C signal suggests that these 14 additional scaffolds are not from a symbiotic species, and not from assembled metagenomes, but are from the sequenced hexactinellid sponge. However, these chromosome-scale scaffolds did not have shared protein homology with other sponge species from Oxford dot-plots, and the intrachromosomal Hi-C signal was unlike that of the 18 conserved chromosome-scale scaffolds. Blastx or blastn searches suggest that these scaffolds were eukaryotic in nature, and not from a co-assembled prokaryotic organism. Many of the top blastx hits were from glass sponges. Given all of these findings, we concluded that the 14 scaffolds likely represent some gene-poor whole chromosomes, or segments of chromosomes, in the hexactinellid sponge genome. Given the lack of knowledge of the chromosome biology of hexactinellid sponges, the two haplotype fasta files we produced represent two hypotheses of the genome of this species. The haplotype A fasta file contains 18 haplotypes of the chromosomes that are conserved between other sponges. The haplotype B fasta file contains the homologous 18 sequences found in the haplotype A fasta file, plus the additional 14 chromosome-scale sequences. For this reason, the genome assemblies of both haplotypes should be used for comparative genomics.

The genome size of the hexactinellid sponge estimated with 19-mers was 141.2 million base pairs (Mbp), with 45.7% unique k-mers, and approximately 1.85% heterozygosity (**Extended Data Fig. 1k**). The haplotype A genome assembly fasta file contains 33 contigs in 18 scaffolds containing 112.4 Mbp. The contig N50 of the haplotype A assembly is 5.4 Mbp and the scaffold N50 is 5.9 Mbp (**Supplementary Table 1.4**). The haplotype B genome assembly fasta file contains 55 contigs in 32 scaffolds containing 195.8 Mbp. The contig N50 of the haplotype B assembly is 5.5 Mbp and the scaffold N50 is 6.5 Mbp. The small scaffolds and duplicated sequences that were removed contain 3,320 contigs in 3,262 scaffolds containing 193 Mbp. The contig N50 of these sequences are 84.7 kilobase pairs (kbp), and the scaffold N50 is 85.8 kbp.

2.3 Supplementary Figures - Sponge genomes

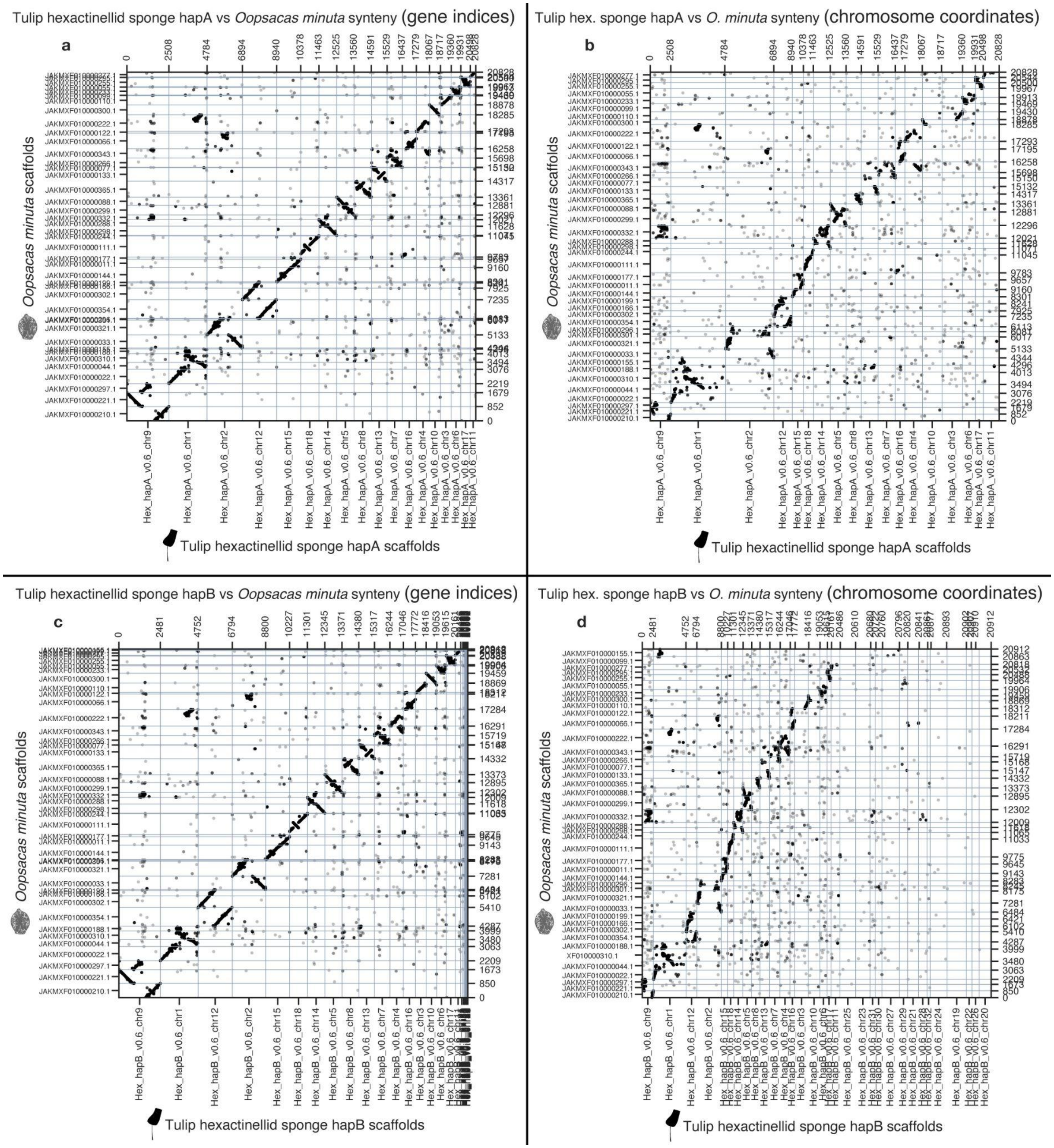

Supplementary Figure 2.1 | **Hexactinellid sponge genome homology. a.-b.** Gene index coordinate and chromosome coordinate Oxford dot plots of tulip sponge haplotype A plotted against the *Oopsacas minuta* genome assembly. **c.-d.** Oxford dot plots of the tulip sponge haplotype B plotted against the *Oopsacas minuta* genome assembly. Only the chromosome-like scaffolds one through eighteen in both the haplotype A and haplotype B fasta files share a high degree of synteny with the *Oopsacas minuta* scaffolds. The colinearity of the independently assembled tulip hexactinellid and *Oopsacas* scaffolds suggests that both sets of scaffolds are well-assembled, and that there have been few genome rearrangements since the divergence of these two species.

2.4 Supplementary Table - cladorhizid sponge

<table>
<tr><th>1:1 Orthologous Chromosome</th><th>Ephydatia mulleri Chromosome Size (Mbp)</th><th>Ephydatia mulleri Chromosome Number</th><th>Cladorhizid Chromosome Number</th><th>Cladorhizid HapA scaffold size (Mbp)</th><th>Cladorhizid HapB scaffold size (Mbp)</th></tr>
<tr><td>1</td><td>34.7</td><td>1</td><td>1</td><td>36.1</td><td>36.1</td></tr>
<tr><td>2</td><td>18.0</td><td>2</td><td>2</td><td>40.2</td><td>41.0</td></tr>
<tr><td>3</td><td>16.8</td><td>3</td><td>3</td><td>43.5</td><td>41.0</td></tr>
<tr><td>4</td><td>14.2</td><td>4</td><td>4</td><td>50.9</td><td>59.8</td></tr>
<tr><td rowspan="14"></td><td>14.0</td><td>5</td><td rowspan="3">5</td><td rowspan="3">45.9</td><td rowspan="3">40.1</td></tr>
<tr><td>8.9</td><td>13</td></tr>
<tr><td rowspan="2">6.3</td><td rowspan="2">23</td></tr>
<tr><td rowspan="2">17</td><td rowspan="2">44.6</td><td rowspan="2">47.1</td></tr>
<tr><td>7.6</td><td>22</td></tr>
<tr><td>14.0</td><td>6</td><td rowspan="2">6</td><td rowspan="2">51.1</td><td rowspan="2">41.0</td></tr>
<tr><td rowspan="2">7.8</td><td rowspan="2">21</td></tr>
<tr><td rowspan="3">7</td><td rowspan="3">46.3</td><td rowspan="3">45.3</td></tr>
<tr><td>12.6</td><td>7</td></tr>
<tr><td>5.0</td><td>24</td></tr>
<tr><td>11.0</td><td>8</td><td rowspan="2">8</td><td rowspan="2">50.2</td><td rowspan="2">46.4</td></tr>
<tr><td>8.5</td><td>17</td></tr>
<tr><td>10.8</td><td>9</td><td rowspan="2">9</td><td rowspan="2">47.6</td><td rowspan="2">43.7</td></tr>
<tr><td>8.6</td><td>15</td></tr>
<tr><td>5</td><td>9.9</td><td>10</td><td>10</td><td>38.8</td><td>40.4</td></tr>
<tr><td>6</td><td>9.9</td><td>11</td><td>11</td><td>55.6</td><td>47.4</td></tr>
<tr><td>7</td><td>9.7</td><td>12</td><td>12</td><td>37.9</td><td>42.3</td></tr>
<tr><td rowspan="4"></td><td>7.9</td><td>20</td><td rowspan="2">13</td><td rowspan="2">43.7</td><td rowspan="2">32.7</td></tr>
<tr><td rowspan="2">8.8</td><td rowspan="2">14</td></tr>
<tr><td rowspan="2">14</td><td rowspan="2">34.7</td><td rowspan="2">34.4</td></tr>
<tr><td>-</td><td>20</td></tr>
<tr><td>8</td><td>8.0</td><td>19</td><td>15</td><td>42.3</td><td>44.7</td></tr>
<tr><td>9</td><td>8.6</td><td>16</td><td>16</td><td>58.6</td><td>47.3</td></tr>
<tr><td>10</td><td>8.3</td><td>18</td><td>18</td><td>41.1</td><td>42.0</td></tr>
</table>

Supplementary Table 2.1 | **Cladorhizid sponge and *Ephydatia muelleri* chromosome numbering.** The chromosome numbers of the cladorhizid sponge were selected to reflect homology with the *Ephydatia muelleri* chromosomes. 10 chromosomes were 1:1 homologs, without any major detectable fusions or fissions between the two species. Orthologous chromosome sizes varied between the assemblies of the two haplotypes, necessitating analyzing both haplotypes at once.

3 Genome sequencing, assembly, and annotation - Unicellular Outgroup Species:

3.1 Methods - Unicellular Outgroup Species

3.1.1 Hi-C library preparation - Unicellular Outgroup Species - Methods

Toward scaffolding the already-published genome assemblies to chromosome-scale, we prepared Hi-C libraries from *Salpingoeca rosetta* (ATCC® PRA-366™), *Capsaspora owczarzaki* (ATCC® 30864™), and *Creolimax fragrantissima* (ATCC® PRA-284™). The American Type Culture Collection (ATCC) samples that we used are the same accessions that were sequenced for the published genome assemblies for those three species[42–44].

We ordered one cell culture for each of the three ATCC accessions listed above. We briefly thawed each sample, aspirated 0.125 mL of cell culture stock per library, and prepared Hi-C libraries using a previously published Hi-C library preparation protocol[64]. For each species we prepared one library with the enzyme DpnII and one library with MluCI. These libraries were sequenced on a NovaSeq 6000 2x150 cycle run at MedGenome, Inc. in Foster City, California.

3.1.2 Genome scaffolding and annotation - Unicellular Outgroup Species - Methods

We scaffolded the existing genome assemblies for *S. rosetta* (GCF_000188695.1), and *C. fragrantissima* (GCA_002024145.1) using the Hi-C sequencing reads with Dovetail Genomics HiRise vAug2019[65]. Because there was a mate-pair-assisted manually curated genome assembly for *C. owczarzaki*, we used the version 4 genome assembly[70], SALSA2 commit cf0fa8e[66], and the Hi-C reads to scaffold the genome. Hi-C heatmaps were prepared as previously described[40]. This pipeline comprises the tools bwa mem 0.7.17[75], pairtools v0.3.0[76], pairix v0.3.7 (github.com/4dn-dcic/pairix), and Cooler v0.8.10[77]. We visualized the Hi-C matrices with HiGlass v1.10.0[67] and PretextView v0.2.4 (https://github.com/wtsi-hpag/PretextView/releases).

Inversions from genome misassembly, and assembly misjoins, were identified using the Hi-C heatmaps. Erroneous sequence inversions were corrected by replacing the region with the reverse complement of that region. Assembly mis-joins were split at the nearest gap of Ns.

In the *Creolimax fragrantissima* assembly we found and removed large regions and scaffolds that had no mapping Hi-C reads, including one megabase-scale region of a scaffold, and one entire chromosome-scale scaffold. Both of these regions corresponded to *C. fragrantissima* scaffold MWQC01000008.1 in the original assembly (GCA_002024145.1). A blastn search of the scaffolds without mapping Hi-C reads revealed many hits to fungal sequences.

In *C. fragrantissima* we performed several manual breaks and scaffold insertions such that every scaffold from assembly version 4[70] was placed on a chromosome-scale scaffold. Evidence of alternate chromosome configurations was used to generate an alternate reference sequence for *Capsaspora*.

We generated coordinates of gene positions along the chromosome-scale scaffolds by mapping the transcripts from the published genome annotation to the new chromosome-scale scaffolds with minimap2 v2.17[84].

3.1.3 Three assembly versions of *Capsaspora owczarzaki* - Unicellular Outgroup Species - Methods

In the *Capsaspora owczarzaki* genome assembly, the Hi-C map revealed that putative chromosome 7 contained two regions with inter-chromosomal puncta consistent with centromeric Hi-C patterns. The configuration of the Hi-C map led us to believe that the sequenced *Capsaspora owczarzaki* strain contains a heterozygous chromosome fusion. For this reason, we opted to generate alternate genome assemblies that represented putative chromosome 7 as two separate sequences. Using alternate assemblies would allow us to test our phylogenetic hypotheses with both of the inferred heterozygous chromosomal configurations.

We refer to the *Capsaspora owczarzaki* presented above in **Supplementary Information Section 3.1.2** as 'capsasporaA'. This assembly contains all of the scaffolds from Denbo et al. 2019[70] on 16 chromosome-scale scaffolds. No additional sequence curation has been performed to remove repetitive or incorrectly assembled regions.

We then generated a version of the genome assembly in which repetitive regions have been removed from the chromosome-scale scaffolds. This assembly version is referred to as capsasporaB. To create this assembly the .pairs files from the Hi-C maps mentioned above were converted to the .hic format with Juicebox Assembly Tools github commit 46c7ed1[68]. Repetitive regions were identified and placed into separate scaffolds using the .hic map with the Juicebox visualization system v1.11.08[115]. Putative *Capsaspora owczarzaki* chromosome 7 was not split into two scaffolds. Therefore, the 'capsasporaB' assembly represents the haplotype containing the heterozygous chromosome fusion.

The 'capsasporaB' assembly represents the haplotype in which the putative chromosome 7 in assemblies 'capsasporaA' and 'capsasporaB' is two separate chromosomes. To produce this assembly, the 'capsasporaB' assembly was split at the appropriate location, based on Hi-C evidence and using the Juicebox visualization system v1.11.08[115].

3.1.4 Putative centromere locations - Unicellular Outgroup Species - Methods

To identify putative centromeric positions from Hi-C data, Hi-C reads were preprocessed and aligned using Juicer[115] v1.5.6-64-g94ec691 and visualized in JuiceBox [69] v1.11.08 at both MapQ0 and MapQ30 contact filtering thresholds. Initial centromere-centromere puncta positions were estimated manually in JuiceBox and refined with Centurion [119] v0.1.0-3-g985439c using Knight-Ruiz-normalized[120] matrices at MapQ0 and 10kb resolution. If a Centurion-estimated (resolution=10000, coef=10) centromere position deviated from the initial, manual estimate by more than 10% [calculated as $100.0 \cdot \text{abs}(P_{\text{Centurion}} - P_{\text{initial}}) / \max(L_{\text{p-arm}}, L_{\text{q-arm}})$; where P is the estimated centromere position and L is the respective chromosome arm length], the output centromere position was reverted to the initial estimate[121].

3.2 Results and Discussion - Unicellular Outgroup Species

3.2.1 Hi-C sequencing summary - Unicellular Outgroup Species - Results

Each Hi-C library of the unicellular outgroup species was sequenced to at least 181x coverage, and up to 821x coverage, of the entire genome size. Because these species have small genomes, this coverage was attained by relatively shallow sequencing of between 20.8 million and 75.4 million read pairs. The percent of reads that contained a linker sequence indicating a successful Hi-C junction capture ranged between 10.3% and 44.1% in individual libraries. See **Supplementary Table 1.1** and **Supplementary Data 1** for a sequencing data summary.

3.2.2 Summary of genome assemblies - Unicellular Outgroup Species - Results

Each of our genome assemblies of the unicellular species contain fewer than 69 scaffolds, and at least 98.5% of the basepairs are in chromosome-scale scaffolds in each assembly (**Supplementary Tab. 1.4**). We found that *Salpingoeca rosetta* has 36 chromosomes, *Capsaspora owczarzaki* has 16 chromosomes, and *Creolimax fragrantissima* has 27 chromosomes (**Extended Data Fig. 5**). The centromere configuration was evident from the Hi-C maps of the *C. fragrantissima* and *C. owczarzaki* genomes. These assemblies were sufficient for use in inter-species chromosome-scale genome comparisons. Hi-C maps and comparisons to the original scaffold-level assemblies are shown in **Extended Data Figure 5g-i**. Final genome assembly statistics can be found in **Supplementary Table 1.4**.

The Hi-C matrix of *Capsaspora owczarzaki* contained signal for a heterozygous fusion of two chromosomes. One haplotype appears to contain two chromosomes fused end-to-end, composing the reference sequence *Capsaspora* chromosome 7 presented here. The alternate haplotype appears to have those two sequences as separate chromosomes.

3.2.3 Putative centromere locations - Unicellular Outgroup Species - Results

Only *C. fragrantissima* and *C. owczarkzaki* had a Hi-C pattern that was consistent with centromere signals found in other unicellular species. Therefore, the *S. rosetta* centromere estimates may be inaccurate. *C. fragrantissima* chromosome 7 (COW7) appears to have two regions associated with intra-chromosomal Hi-C hotspots. Future long-read sequencing efforts of the *Capsaspora* genome will be necessary to resolve the correct location of the centromeres on COW7. A table of putative centromere locations is in **Supplementary Table 3.1.**

Scaffold Number	Estimated *S. rosetta* centromere pos. (bp)	Estimated *C. fragrantissima* centromere pos. (bp)	Estimated *C. owczarzaki* assembly A centromere pos. (bp)	Estimated *C. owczarzaki* assembly B centromere pos. (bp)	Estimated *C. owczarzaki* assembly C centromere pos. (bp)
1	1185201	3885213	2810494	994617	994617
2	1975063	1770743	1545387	1104440	1104440
3	1231465	1400598	1602947	913706	913706
4	1169491	1478781	792781	810051	810051
5	443559	1097516	1340158	831078	831078
6	1220646	1565874	1488804	238541	238541
7	1715717	1023063	855936	204998<br>636624	204998
7b					129864
8	1511511	1467500	801448	813965	813965
9	257959	988242	1029780	479051	479051
10	780700	1346737	606463	588695	588695
11	943569	374410	804060	488552	488552
12	1384598	780145	501091	496490	496490
13	189623	331909	687713	283166	283166
14	1469194	1198569	491731	435614	435614
15	1125372	1223970	534250	396859	396859
16	790173	76818	511862	386500	386500
17	722278	78413			
18	1170119	1086172			
19	885200	1046705			
20	1203659	1047460			
21	1138189	810639			
22	5000	708873			
23	493330	745616			
24	32751	595222			
25	617076	530000			
26	395875	211415			
27	762959				
28	789207				
29	70685				
30	371498				
31	526128				
32	710970				
33	178356				
34	351173				
36	130290				
36	292335				

Supplementary Table 3.1 | **Estimated centromere positions in unicellular species.** The coordinates, in basepairs, of the estimated centromere positions in *S. rosetta*, *C. fragrantissima*, and *C. owczarzaki*.

4 Chromosome tectonic events and their phylogenetic implications

4.1 Introduction

Heritable changes in karyotype are caused by three major physical processes that change the macro structure of chromosomes: splitting single chromosomes into two (fissions), fusing two chromosomes into one (fusions), or swapping sections between chromosomes (translocations). While we use "fission" and "fusion" as coarse descriptions to simplify discussion, these terms should be understood as shorthand for processes that involve multiple double strand breaks and ensure that each resulting (non-holocentric) chromosome has a single centromere and two functioning telomeres. For further discussion see Schubert and Lysak (2011)[51]. In addition to these interchromosomal processes, changes in the linear sequence along chromosomes are caused by chromosomal inversions, in which a segment of a single chromosome is reversed. Over evolutionary time, chromosomal inversions cause the order of genes to rearrange dramatically, as is visible between homologous chromosomes in extant species diverged over tens to hundreds of millions of years[12,32,57].

Previous work has explored the idea that chromosome fusion events, followed by gene order rearrangement from many chromosomal inversions, is (a) an irreversible process, and (b) is heritable, and (c) is therefore phylogenetically informative[12]. Here, we will explore these ideas in the context of the ctenophore-/sponge-sister hypotheses, we will discuss why no other series of "chromosome algebra" moves is phylogenetically informative, and lastly we will consider possible caveats of using chromosomal fusion-with-mixing events as phylogenetically informative markers.

4.2 Assumptions

In this mode of phylogenetic analyses it is assumed that one knows at least one outgroup of the clade that is desired to study. For example, if one were trying to resolve a phylogenetic polytomy within the Bilateria, one could use species of Cnidaria, Porifera, or from more deeply diverged Opisthokonts as outgroups. In this manuscript, we work under the assumption that the Metazoa (Porifera, Ctenophora, Cnidaria, Placozoa, Bilateria) are monophyletic, that choanoflagellates are the closest relatives of the Metazoa[48,122], that the Filasterea are the closest relatives of a monophyletic clade containing the choanoflagellates and the Metazoa[49,122], and that the Ichthyosporea are the closest relatives of all of the above mentioned clades[49,122] (**Supplementary Fig. 4.1**).

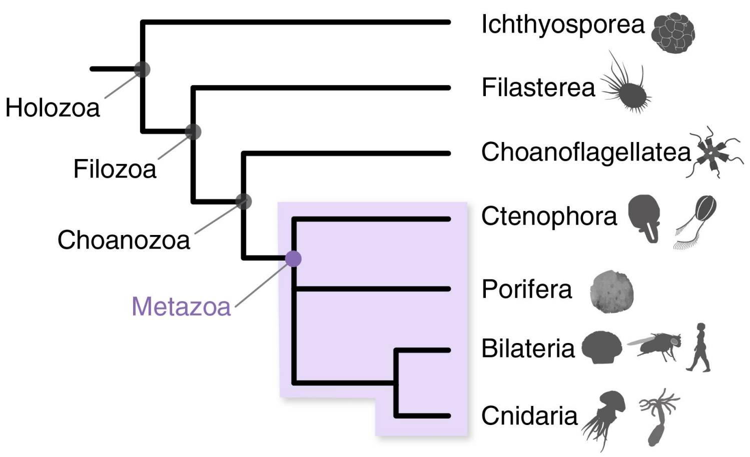

Supplementary Figure 4.1 | **The assumptions of the phylogenetic methodology used in this manuscript.** This phylogenetic tree is the assumption under which the evolutionary hypotheses presented in this manuscript are presented. Namely, that the Metazoa are monophyletic, and that the choanoflagellates, the filastereans, and ichthyosporeans are outgroups of the Metazoa.

4.3 Oxford dot plots allow identification of synteny between two genomes

To understand the relationship between the genomes of two or more species, it is essential to first identify orthologous regions between the chromosomes, and to infer the evolutionary history of those chromosomes. For closely related species, one can identify orthologous genome regions by performing whole-genome nucleotide alignments. For distantly-related species, it is instead possible to use protein sequence comparisons to create a map of orthologous positions between any given pair of chromosomes. Even if two species diverged hundreds of millions of years ago, it is possible to detect orthologous chromosomes, or regions of chromosomes, using protein orthology. Statistical significance can be established using Fisher's exact test[57]. One can then visualize the synteny relationships between these two species by plotting the orthologous protein coordinates. These plots depicting the synteny relationships between two species are called Oxford dot plots[57] (**Supplementary Fig. 4.2a**). In general, it is impossible to determine the ancestral state of the common ancestor of the two species depicted in Oxford dot plots without consideration of the relationship of these orthologs to the genome of outgroup species. (The exception is a 1:1 relationship between chromosomes of different species, which established that such a chromosomal unit was present in their common ancestor, which can be inferred even without considering outgroups.) Oxford dot plots are useful to characterize the relationship between the genomes of two species, and this information can guide downstream analyses and comparisons.

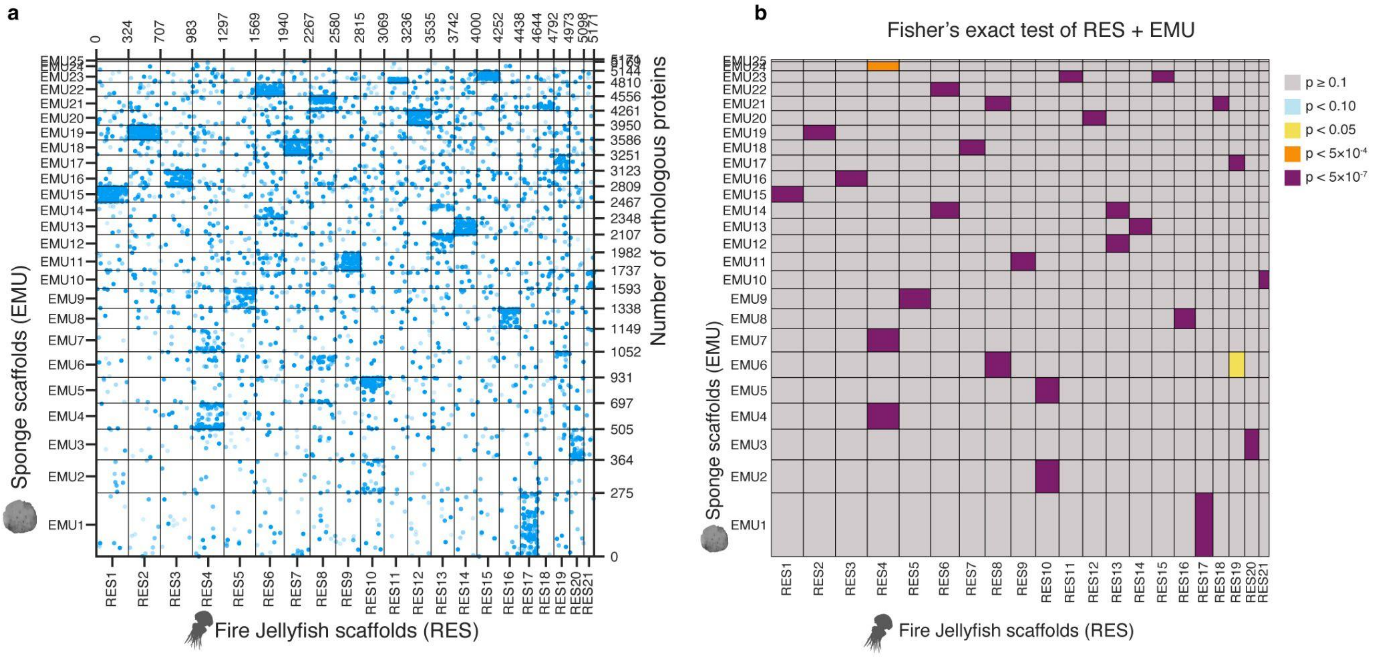

Supplementary Figure 4.2 | **Example Oxford dot plot between two species, with significance test. a.** depicts an Oxford dot plot of orthologous proteins identified between the fire jellyfish, *Rhopilema esculentum* (RES), and a freshwater demosponge, *Ephydatia muelleri* (EMU). Orthologous chromosomes are identifiable from many orthologous proteins being contained in one chromosome-chromosome bounding rectangle. Relationships such as chromosome fusions/fissions/translocations (RES4 composed of EMU4 and EMU7), and decay of synteny from small translocations (diffuse dots on non-homologous chromosomes) can be also seen in these figures. **b.** A one-sided Fisher's exact test with Bonferroni correction[57] calculates a *p*-value that two chromosomes are categorically similar compared to other chromosomes. The biological interpretation of this analysis is that a pair of chromosomes with a small *p*-value in the Fisher's exact test are derived from the same ancestral piece of DNA: they are homologous. Rows or columns with two or more significant *p*-values suggest a complicated evolutionary history involving fusion/fission/or translocation events.

4.4 Ancestral Linkage Group identification in three or more species

Performing orthology identification between three or more species provides a framework from which one can make inferences about chromosome evolution. Two species can be compared to a known outgroup and derived fission, fusion, and translocation events can be inferred from those relationships. Protein orthology offers the ability to identify orthologous genome positions between species that have diverged more than hundreds of millions of years ago, as we do in this manuscript for animals + *Salpingoeca* or animals + *Capsaspora*[50,123].

One stringent ortholog identification method is to use reciprocal-best blastp searches of two or more species (see Moreno-Hagelsieb and Latimer 2008[124], **Supplementary Information 5.2.3**). Each new species *n* added to the reciprocal-best blast search increases the number of reciprocal best blast pairs that must be satisfied by $n(n - 1)/2$. Therefore, the stringency for this orthology-finding method increases non-linearly with additional species. Once a set of orthologs have been inferred from *n* species, it is possible construct protein hidden Markov models to expand the orthologs to more species with the increased stringency imposed by requiring reciprocal-best blastp hits (**Supplementary Information 5.2.3,5.2.5**). Another common tool for ortholog inference for many species is OrthoFinder, which relies on blast-like tools to identify protein similarities, then uses downstream clustering and tree analyses to form "orthogroups"[98]. We use both methods of orthology inference in this manuscript.

Once protein orthologs have been identified in a group of organisms, it is necessary to first select only orthologs for which each species has only one protein, or if two or more proteins are present in the ortholog, the corresponding genes are located on the same chromosome. This filtering process removes the ambiguity in downstream analyses in which it is necessary to decide to which chromosome an ortholog belongs in each species. In analyses that use OrthoFinder, we perform this filtering step (described later in **Supplementary Information 10**). Since our ortholog-finding technique using hidden Markov models only finds one protein per species per orthologous group, no further filtering is necessary.

Now, with a collection of orthologs for many species, there will be many possible combinations of chromosomes on which the genes reside in each ortholog. For example, with the four species A, B, C, and D, we may see an ortholog whose proteins exist on chromosomes (**Supplementary Table 4.1**):

Supplementary Table 4.1 | **Hypothetical orthologs and chromosome coordinates from four species.**

ortholog	A chrom.	B chrom.	C chrom.	D chrom.
1	A1	B1	C1	D1
2	A1	B1	C1	D2

These two example orthologs have proteins on the same chromosomes in A, B, and C, but on different D chromosomes. With only these two orthologs, we have no way of determining which combination of chromosomes may represent some ancestrally linked genes, if either, or if their distribution on chromosomes is due to random chance.

Consider instead the following table (**Supplementary Table 4.2**):

Supplementary Table 4.2 | **More hypothetical orthologs and chromosome coordinates from four species.**

ortholog	A chrom.	B chrom.	C chrom.	D chrom.
1	A1	B1	C1	**D1**
2	A1	B1	C1	D2
3	A1	B1	C1	D2
4	A1	B1	C1	D2
5	A1	B1	C1	D2
6	A1	B1	C1	D2

In this table, we see that the configuration of orthologs on chromosomes A1-B1-C1-D2 occurs 5 times, and the configuration A1-B1-C1-D1 occurs only once. If we find that there are some unique pairs of combinations of chromosomes that exist more than we expect by chance, then these groups are likely to be ancestral linkage groups of genes (ALGs) (see Simakov et al. 2020[57] and Simakov et al. 2022[12] for further description).

In the analyses for this manuscript, we select cutoffs for the minimum size of ALGs to consider by simulation. We then randomly shuffle the genomes in the analysis and calculate the frequency of seeing ALGs of a given number of orthologs due to random chance. This minimum ALG size is smaller for deeply-diverged species that share little synteny, and larger for species that have diverged more recently.

The ALGs that remain after filtering out those that are small enough to be expected due to random chance are then candidates to consider for studying chromosome tectonic events.

4.5 Phylogenetic implications of chromosome tectonic events identified with 4-species ALGs

There are seven basic configurations that are often present when comparing ALGs found from four species, with three species (A, B, C) in a monophyletic clade and one outgroup (O) (**Extended Data Fig. 4**). Each of these seven configurations can be explained by a chromosomal fusion or fission, and in the cases of fusions, subsequent serial chromosomal inversions. Four of the seven configurations provide no information that can help polarize the relationship between the clade (A, B, C) (**Extended Data Fig. 4a,b,d,e,g**). One of the two remaining configurations is most easily explained by a single chromosome fission event that is likely a synapomorphy, but with a chance of homoplasy (**Extended Data Fig. 4f**). Only one configuration of these seven types is an irreversible change[12] and, as we show later, is more likely to arise from an ancestral event rather than convergence (**Extended Data Fig. 4d**). In the following sections we will explore each configuration in more detail.

In the following scenarios, we use the single letter "O" to refer to the outgroup, and species A, B, and C are the species that we would like to study. In the tables below there are no longer columns labeled "ortholog" because the orthologs are now grouped into each row. Instead there is now a column labeled "ALG" to differentiate between the unique combinations of chromosomes.

4.5.1 Fusion with mixing in two non-outgroup species

Let us first consider the only chromosome configuration of those shown above that provides evidence of an evolutionary synapomorphy: the chromosomal fusion-with-mixing[12] (See **Extended Data Fig. 4c**). The assumption of this hypothesis, and all those below, is that species A, B, and C form a monophyletic clade, or in other words that we know the outgroup for the analysis.

Supplementary Table 4.3 | **ALG table of a putative fusion-with-mixing event.**

O chrom.	A chrom.	B chrom.	C chrom.
O1	A1	B1	C1
O2	A2	B1	C1

Supplementary Table 4.3 shows that species B and C share two ALGs on the same chromosomes, while the two ALGs are present on separate chromosomes in species A and in the outgroup. An ribbon plot and Oxford dot plot of the positions of the orthologs along the chromosomes shows that the two ALGs are not only present on the same chromosomes in species B and C, but that the genes in those two ALGs are intermingled along the chromosomes (**Supplementary Figure 4.3b,c**). Given the irreversibility of chromosomal fusion-with-mixing events[12], and the less likely explanations of convergence (**Supplementary Figure 4.3d,e**), the most plausible explanation for these data is that species A is the sister clade of species B and C, joined together by a fusion-then-mixing event.

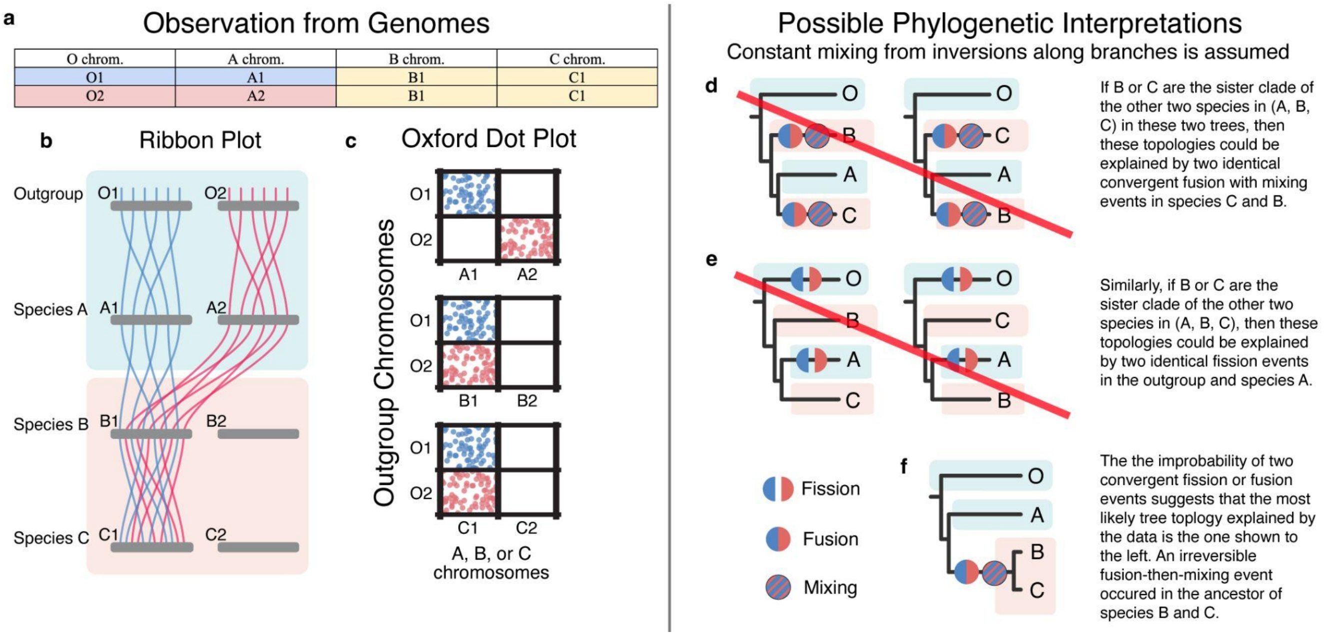

Supplementary Figure 4.3 | **ALGs involved in a fusion-with-mixing event.**
a. The table of two ALGs presented in **Supplementary Table 4.3**. **b.** A cartoon depiction of the orthologs plotted in the context of the positions on the chromosomes, and **c.** the orthologs plotted in two dimensions as Oxford dot plots. **d.** and **e.** show unlikely tree topologies caused by convergent fusions or fission events, while **f.** shows the most likely tree topology that requires the fewest number of chromosomal tectonic events.

4.5.2 ALGs with unique combinations of chromosomes

Let us now consider a scenario in which an ALG is recovered that has no shared chromosomes with other species (See also **Extended Data Fig. 4a**).

Supplementary Table 4.4 | **A single ALG with a unique combination of chromosomes.**

O chrom.	A chrom.	B chrom.	C chrom.
O1	A1	B1	C1

In this scenario, the only information present is that there are many genes conserved on the combination of chromosomes O3-A4-B3-C1. There are no other groups that were recovered. A single ALG contains no phylogenetic information to help resolve the polytomy of species A, B, and C. There are no chromosomal tectonic events to consider in the divergence of this conserved piece of DNA since the divergence of the common ancestor of O, A, B, and C. However, single ALGs are informative in that they are the remainder of a group of genes that were present on single chromosomes in the common ancestor of these species, and still persist today on the same chromosomes since divergence.

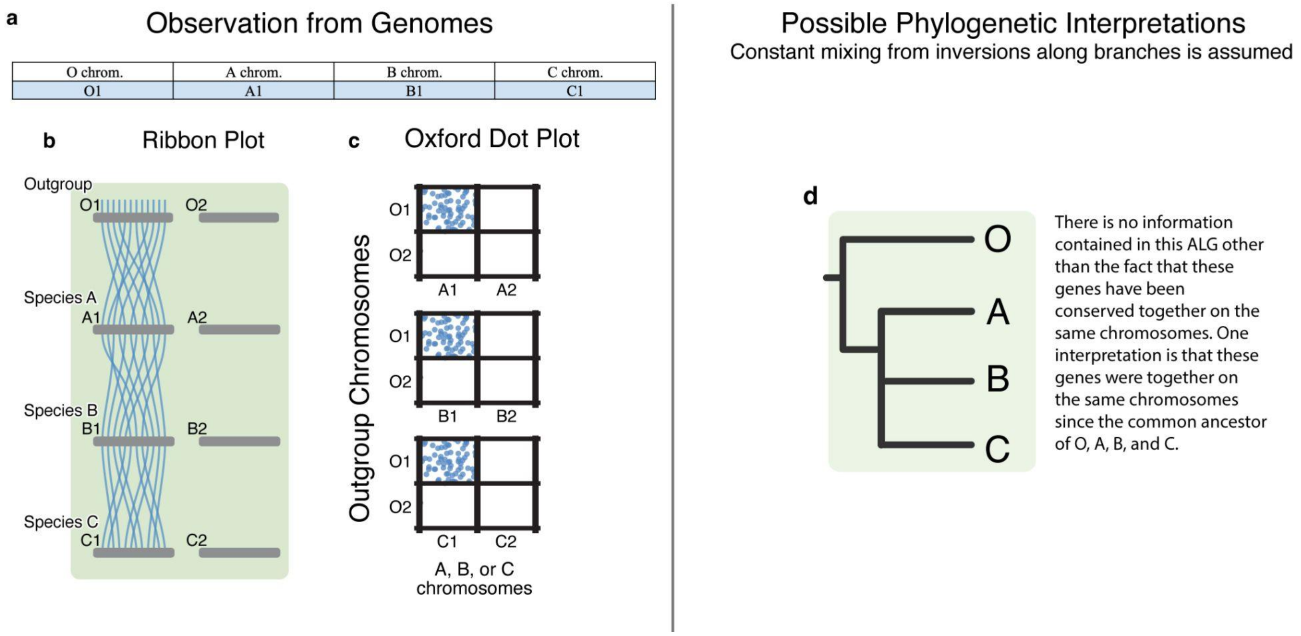

Supplementary Figure 4.4 | **Phylogenetic interpretations of ALGs with unique chromosome combinations**
a. There is only one ALG in the table. The genes in the ALGs exist on single chromosomes in all three species, shown in an **b.** ribbon plot, and **c.** an Oxford dot plot. **d.** There is no phylogenetic information in this ALG to help resolve the polytomy between species A, B, and C.

4.5.3 One fission event in the outgroup / One fusion in the ancestor of other species

Another hypothetical scenario is that two ALGs share the same chromosomes for species A, B, and C, but separate chromosomes for the outgroup (See also **Extended Data Fig. 4b**).

Supplementary Table 4.5 | **One fission event in the outgroup / One fusion in the ancestor of other species.**

O chrom.	A chrom.	B chrom.	C chrom.
O1	A1	B1	C1
O2	A1	B1	C1

There are two phylogenetic scenarios with only one chromosomal tectonic event that can explain the chromosome configuration of these two ALGs. One possibility is that there was a single ancestral fusion or translocation event that occurred in the branch leading to the clade containing species A, B, and C (**Supplementary Figure 4.5g**). The other possibility is that there was a chromosomal fission event in the branch leading to the outgroup (**Supplementary Figure 4.5g)**. All alternate explanations of the data involve multiple independent fusion events. For example, the same fusion event could have occurred convergently in the three lineages leading to species A, B, and C, since the divergence from their common ancestor (**Supplementary Figure 4.5f**). Other similar trees that could be explained by two convergent fusion events are also unlikely.

For all of these possible scenarios it is impossible to use this information to resolve the topology of the subtree containing species A, B, and C (**Supplementary Figure 4.5d,e**). There are no synapomorphy-forming events that can help polarize one of the species as having the ancestral state, while the other two have a shared derived and irreversible state.

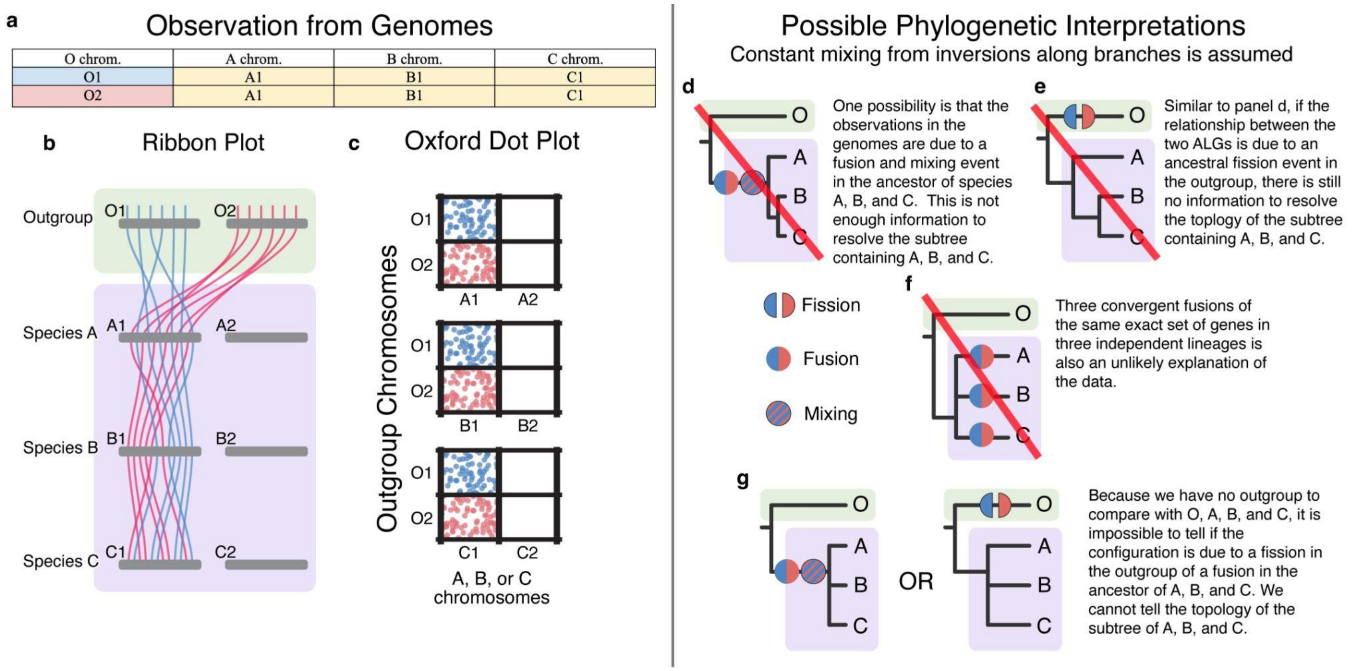

Supplementary Figure 4.5 | **An ambiguous scenario of an ancestral fusion or derived fission.**
a. There is only one ALG in the table. The genes in the ALGs exist on single chromosomes in all three species, shown in an **b.** ribbon plot, and **c.** an Oxford dot plot. **d.** and **e.** There is no phylogenetic information in this ALG to help resolve the polytomy between species A, B, and C. **f.** is unlikely due to the improbability of three convergent fusions. **g.** The topology can be explained by two equally probable single chromosomal tectonic events.

4.5.4 Two ALGs fused in a single ingroup species

One possibility is that two ALGs exist on separate chromosomes in the outgroup species, and two of the species in (A, B, C) (See also **Extended Data Fig. 4d**). On a third species in (A, B, C), the genes from the two ALGs are on the same chromosome and intermingled.

Supplementary Table 4.6 | **ALGs of a putative derived fusion with mixing.**

O chrom.	A chrom.	B chrom.	C chrom.
O1	A1	B1	C1
O2	A2	B2	C1

A fusion event that is only present in one of the ingroup species does not provide enough information to resolve the tree topology (**Supplementary Figure 4.6d**). Alternative explanations that involve convergent fission events are less likely than a single fission event, and also do not resolve the tree topology (**Supplementary Figure 4.6e**). Given that the ancestral state appears to be split, the fewest number of chromosome tectonic events that can explain the data is one: one fusion event in the ancestor of species C since its divergence from the common ancestor of (A, B, C) (**Supplementary Figure 4.6f**).

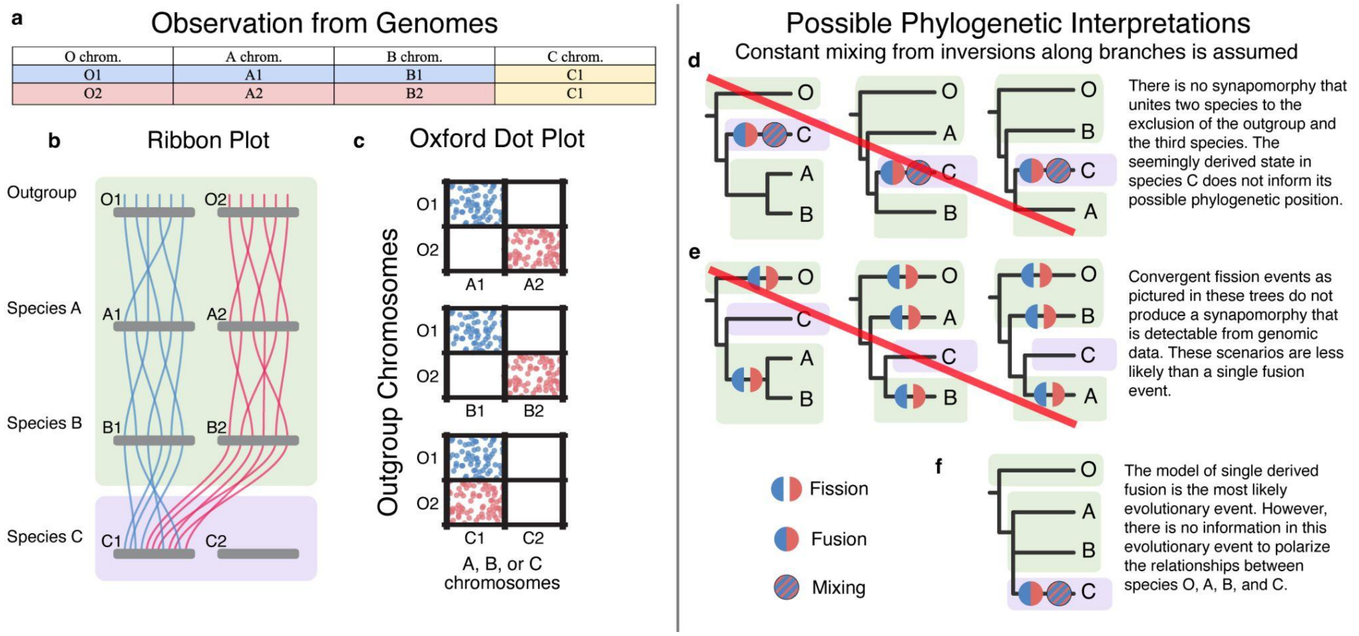

Supplementary Figure 4.6 | **Phylogenetic interpretations of two ALGs mixed only in one species.**
a. The table shows two ALGs that are on separate chromosomes in the outgroup and two species in (A, B, C). **b.** A cartoon ribbon plot of the ALGs. **c.** A cartoon Oxford dot plot of the ALGs. **d.** The possible chromosomal tectonic events that can explain these ALGs do not aid the resolution of the tree topology for (A, B, C). **e.** Explanations of these ALGs using convergent events are unlikely. **f.** The most likely explanation for the data are a derived fission event in the branch leading to C, however it is impossible to resolve the topology of the tree.

4.5.5 Two ALGs appear by fission in one ingroup species

Let us now consider the phylogenetic implications of two ALGs that exist on the same chromosomes in the outgroup, in species A, and in species B. In species C, however, these ALGs exist on two separate chromosomes. (See also **Extended Data Fig. 4e**).

Supplementary Table 4.7 | **A putative fission event in the ancestor of one species.**

O chrom.	A chrom.	B chrom.	C chrom.
O1	A1	B1	C1
O1	A1	B1	C2

The fewest number of chromosomal tectonic events that would be required to produce this structure between four genomes is a single fission event in species C (**Supplementary Figure 4.7d,f**). However, a derived fission in a non-outgroup species does not provide any evidence of a synapomorphy between two of the species in A, B, and C – the derived change could happen in any of the pictured topologies in **Supplementary Figure 4.7d**, therefore we must reject these topologies. Convergent fusion events explaining the ALGs also do not provide information to resolve the topology of (A, B, C). In addition, convergent fusion events are less likely to occur than a single fission event.

If this were the only information available to use from the outgroup, species A, species B, and species C, then the most likely tree topology is shown in **Supplementary Figure 4.7f**. (In any event, we do not find such events in our analysis of a diverse set of animal and unicellular outgroup genomes.)

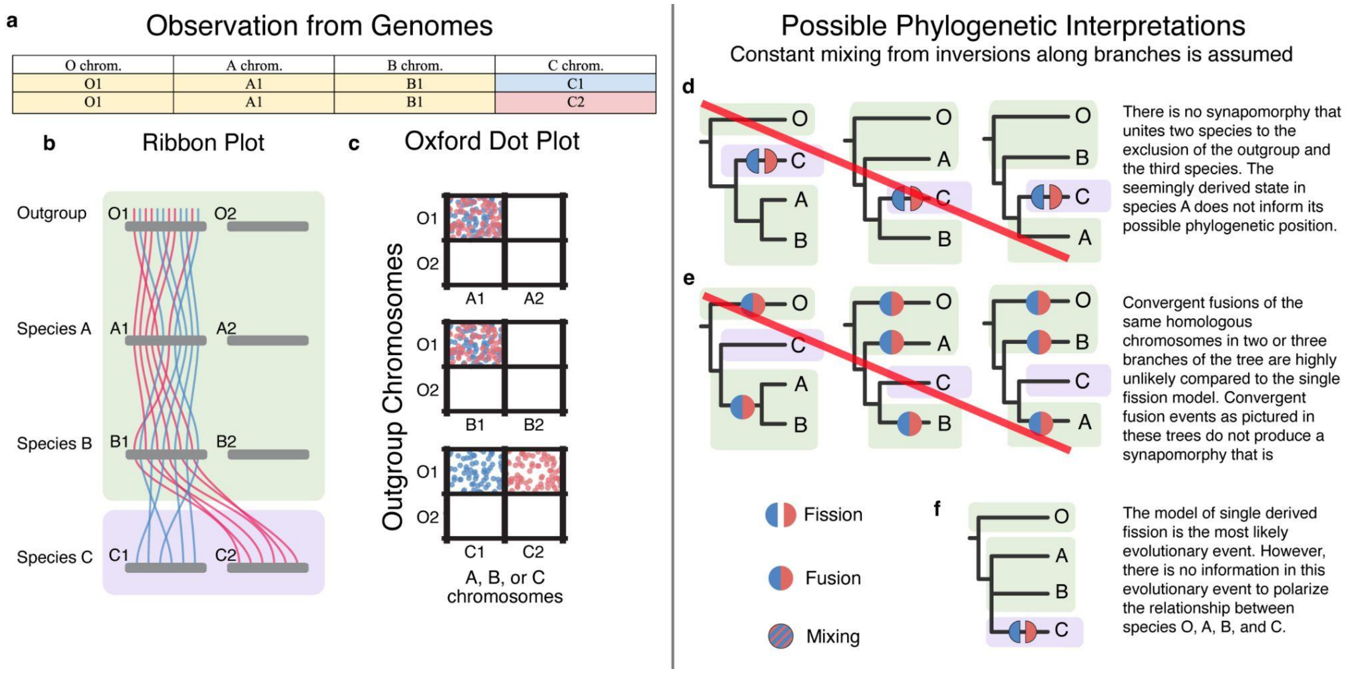

Supplementary Figure 4.7 | **Phylogenetic interpretation of two ALGs with one unfused species.**
a. the table of ALGs. **b.** The cartoon ribbon plot of the ALGs. **c.** The cartoon dot plot of the ALGs. **d.** A single fission event in species A, B, or C does not create a synapomorphy that informs the tree topology. **e.** Convergent explanations are less likely than a single fission event. **f.** Single derived fission events are the most likely evolutionary explanation.

4.5.6 One fission event in the ingroup lineage

Consider two ALGs that exist, and are mixed, on the same chromosomes in the outgroup and species A, but are on separate chromosomes in species B and C (See also **Extended Data Fig. 4f**).

Supplementary Table 4.8 | **Putative ancestral fission event in the ancestor of two species.**

O chrom.	A chrom.	B chrom.	C chrom.
O1	A1	B1	C1
O1	A1	B2	C2

In this scenario, species A shares the same state as the outgroup (two ALGs mixed on one chromosome), while species B and C have a different state than the outgroup and species A (the two ALGs on separate chromosomes). First, consider the possibility that species B is sister to a clade containing (A, C), or that species C is sister to a clade containing (A, B) (**Supplementary Figure 4.8d,e**). If this is the correct topology, then it would require convergent fusion events (**Supplementary Figure 4.8d**) or convergent fission events (**Supplementary Figure 4.8e**).

Convergent fusion or fission events are less likely than a single fusion event that occurred in the branch leading to a clade containing species B and C. (Similar to **Supplementary Information 4.5.5**, we do not find any such events in our analysis of animal and unicellular outgroup genomes.)

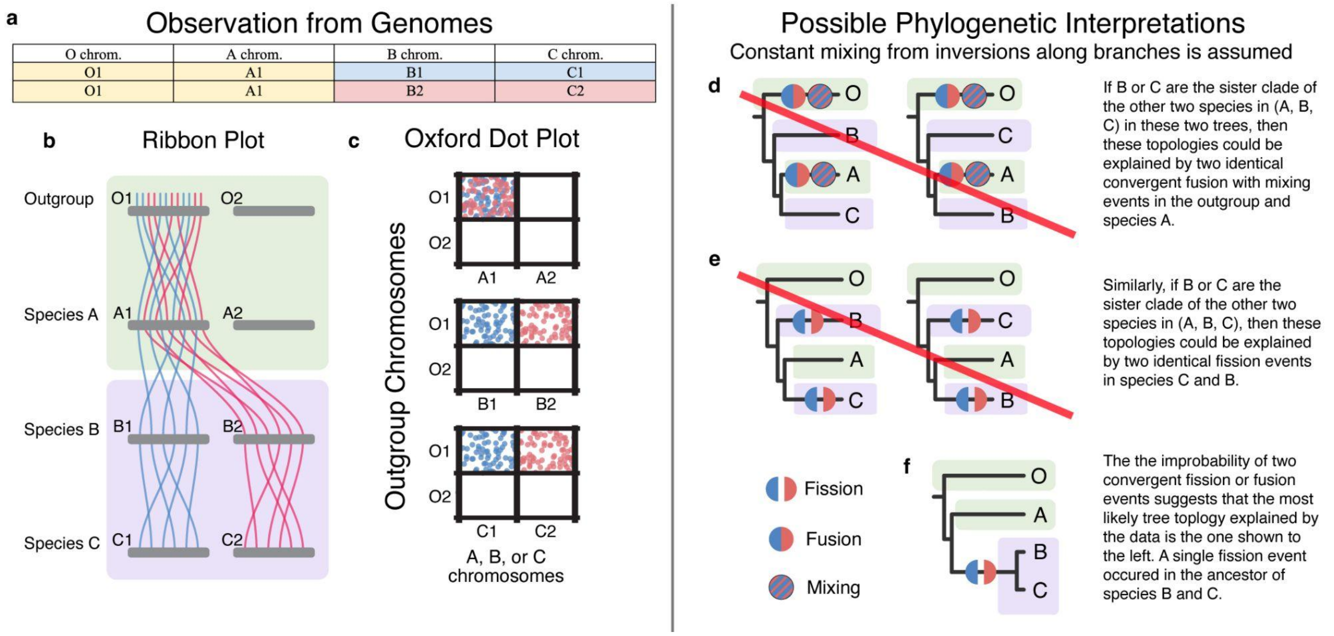

Supplementary Figure 4.8 | **Phylogenetic interpretations of an apparent derived fission.**
a. the table of ALGs. **b.** The cartoon ribbon plot of the ALGs. **c.** The cartoon dot plot of the ALGs. **d.** and **e.** Convergent fusion or fission events with B or C as the sister group to the others in (A, B, C) are not likely topologies. **f.**One single derived fusion event is the most likely evolutionary explanation.

4.5.7 One fission event in the ingroup lineage

Let us now consider the phylogenetic implications of finding evidence for one putative chromosome fission event in the outgroup (See also **Extended Data Fig. 4g**).

Supplementary Table 4.9 | **Fusion in the branch leading to the outgroup, or an ancestral fission.**

O chrom.	A chrom.	B chrom.	C chrom.
O1	A1	B1	C1
O1	A2	B2	C2

A fission event that is only present in one of the species in (A, B, C) is not enough information to resolve the tree topology (**Supplementary Figure 4.9d**). A fusion-with-mixing event in the branch leading to the outgroup is also not enough information to resolve the subtree topology (**Supplementary Figure 4.9e**). It is unlikely that two or three convergent fission events could explain the evolutionary history of these ALGs (**Supplementary Figure 4.9f**) when there are alternative explanations that require only one chromosomal tectonic event (**Supplementary Figure 4.9g**). Given that the ancestral state appears to be split, the fewest number of chromosome tectonic events that can explain the data is one: one fusion event in the ancestor of species C since its divergence from the common ancestor of (A, B, C) (**Supplementary Figure 4.9f**).

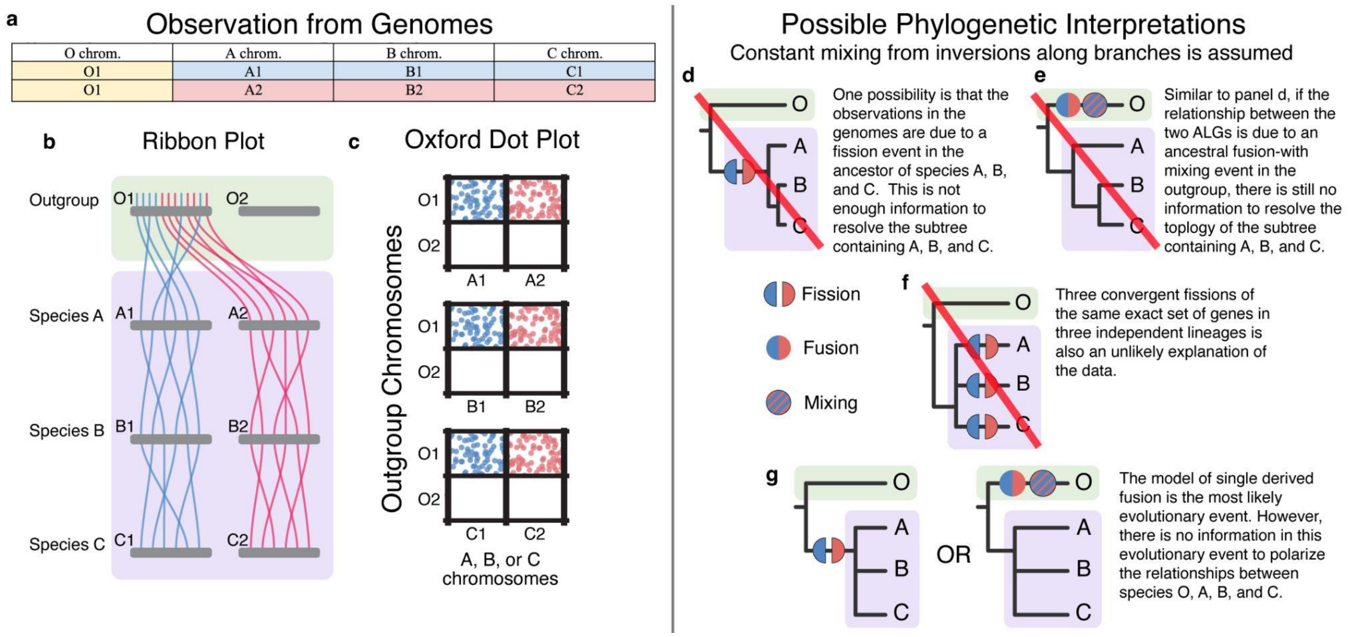

Supplementary Figure 4.9 | **Phylogenetic interpretations of two ALGs fused in the outgroup.**
a. the table of ALGs. **b.** The cartoon ribbon plot of the ALGs. **c.** The cartoon dot plot of the ALGs. **d.** The evolutionary history of these ALGs is best explained by a single fission event or e. a single fusion-with-mixing event. Neither can help resolve the topology of (A, B, C). **f.** Multiple fission events are less likely to explain the configuration of the ALGs. **g.** The configuration of the ALGs can be explained equally well by a fission in the ancestor of (A, B, C) or a fusion-with-mixing in the branch leading to the outgroup.

5 ODP: software to perform macrosynteny analyses

5.1 Introduction

We sought to detect whether unicellular outgroup species to animals shared synteny with the major clades of animals for which we have chromosome-scale genomes: ctenophores, sponges, cnidarians, and bilaterians. Toward this goal we developed a software package using existing techniques[12,57] to identify if there were groups of conserved genes between these clades. The analysis of the genomes of animals, and unicellular outgroup species of animals, using this software indicated that there were phylogenetically informative chromosome fusion-then-mixing events that united sponges, cnidarians, and bilaterians in a monophyletic group. We then further developed the software package to analyze the statistical significance of these findings, and whether alternate hypotheses could explain the findings.

5.2 Methods - Macrosynteny Analyses

5.2.1 Software implementation of synteny analyses - Methods

We developed a software package called `odp` (Oxford dot plots) to perform the analyses related to chromosome-scale synteny and gene linkage group fusion-then-mixing events (github.com/conchoecia/odp). The software contains: (1) scripts to find reciprocal-best-hit proteins between species, (2) scripts to generate chromosome-scale synteny plots between two species, (3) scripts to calculate the likelihood that whole or partial chromosomes are orthologous between two species, (4) scripts to find *n*-way reciprocal best protein hits, in other words highly conserved orthologs, between *n* species, (5) scripts to find significantly preserved gene linkage groups between *n* species, (6) scripts to merge orthologous gene groups between similar searches, (7) scripts to convert the orthologs to hidden markov models (HMMs) to search for orthologous genes in more species, (8) scripts to calculate how well-mixed pairs of linkage groups are along one species, (9) and scripts to perform simulations that test for varying evolutionary hypotheses. These scripts also include provisions to test for the presence and significance of the phylogenetically diagnostic chromosome fusion-then-mixing events.

The `odp` package was designed for reproducibility and scalability. Each script is a snakemake pipeline that is controlled by a yaml configuration file with a unified format. In other words, after making an initial configuration file for the datasets to analyze, it takes little effort to perform subsequent analyses with different scripts quickly. The number of species included in the analyses is only limited by compute time and disk space due to the scalability of snakemake.

The file formats used as input for `odp` are genome assembly fasta files, fasta files containing protein sequences, and files in a GFF-like format specifying the protein locations in the genomes. Internally, `odp` scripts use only four simple, new file formats to convey information between different pipeline steps. Reciprocal best blast hits between two or more species are encoded in tab-separated files, with the file suffix `'.rbh'`, that contain simple gene coordinate information.

The `.rbh` files can then be processed to group together groups of genes present on the same set of chromosomes in the species in the analysis. This, in effect, finds groups of genes linked together since the common ancestor of said species. These files also contain significance values calculated for each linked gene group from genome shuffling simulations. These files are labeled with the suffix `'.groupby'`, and are useful for analyzing chromosomal evolutionary events on a phylogenetic tree.

The `.groupby` files can be merged between multiple analyses. For example we merged the SRO-HCA-EMU-RES and COW-HCA-EMU-RES, joining the tables on orthologs shared on the same

sets of chromosomes in HCA-EMU-RES. The `.groupby` files can also be unwrapped back to `.rbh` files, and any columns or annotations added to the `.groupby` file will be present in the appropriate rows in the new `.rbh` file.

We note that performing *n*-way reciprocal best blast searches between more than two species is highly conservative, and each species added reduces the number of orthologs found. Therefore, if the user wishes to include many species in orthology analyses, it is possible to first conduct a reciprocal best hits search with three or four core species, then find the most closely related genes in additional species. This process builds an HMM from a protein alignment from the orthologs in the 2-/3-/or 4-species `.rbh` file, then uses the HMM to find the best hit for each ortholog in each additional species. The resulting file is another `.rbh` file.

More documentation for the software package can be found on the github page (https://github.com/conchoecia/odp). The version of the software available at publication time is also available on Dryad: https://doi.org/10.5061/dryad.dncjsxm47. In the following methodology, we will refer to the `odp` script that we used for each analysis.

5.2.2 Genome selection and data preparation - Methods

In addition to the genomes that we assembled for this manuscript, we gathered chromosome-scale genomes of cnidarians, sponges, ctenophores, and bilaterians for gene linkage group and macrosynteny analyses. We also used non-chromosome-scale genomes where necessary, namely the genome of the placozoan *Trichoplax adhaerens*. For each chromosome-scale genome assembly, we refer to individual chromosomes using the three-letter code for the genus and species, then the number. We used the chromosome numbers provided in the original manuscripts. For *Trichoplax*, we refer to the scaffolds using the name in the original assembly[102]. The genome annotation files were parsed to create GFF-like files used in `odp` for synteny analyses. See **Supplementary Table 7.1** for genomes used, three-letter species codes, and the publication source of the assembly.

5.2.3 Two-way and *n*-way reciprocal best blastp searches - Methods

We used blastp v2.10.0+[97] to blast all proteins from one species against all proteins of another species. We did not use diamond blastp because we found that it was less sensitive to distantly related proteins. These blast results were parsed to find proteins that were reciprocal best hits (rbh) between each pair of species. The best blast hit was defined as the hit with the highest bit score, then the smallest e-value, for each query. Two-way reciprocal best blast hits are implemented in the scripts `odp` and `odp_nway_rbh`.

We also scaled the analysis to enable rbh blast searches between more than two species. We used blastp to perform searches for all possible database-query combinations given *n* species. Therefore, we refer to this method as *n*-way rbh searches. These searches yield sets of proteins from *n* species that are highly conserved among all *n* species. In effect, this is a highly conservative search of single-copy orthologs between the *n* species.

For any given *n*-way rbh search, the blastp results are loaded into a graph in which proteins are nodes and best blast hits are directional edges. The graph is pruned to remove ties of best hits based on bitscore and evalue from the blast results. The sets of proteins that are *n*-way reciprocal best hits are connected components of degree *n*, and are bidirectionally complete. These sets of proteins are taken from the graph and printed as a `.rbh` file for further processing. This algorithm is implemented in the script `odp_nway_rbh`.

5.2.4 Plotting synteny between two species

Using the reciprocal-best blast results from the above species, we plotted Oxford Dot Plots of each species pairing to visualize synteny using `odp`. We calculated the significance that any two chromosomes in either species shared homology using a Bonferroni-corrected Fisher's Exact Test[57]. We also produced ODP plots in which each dot was assigned a color based on its ortholog in one of the 29 groups of ancestrally linked genes ALGs conserved between cnidarian, sponges, and bilaterians[12]. If there was no ortholog for those proteins in the ALGs, then the dot was colored gray. This enabled us to visualize not only the synteny of two species, but how each chromosome was related to the ancestral linkage groups. This also helped us visualize ALG splits across multiple ctenophore chromosomes.

5.2.5 Identifying reciprocal best hits in additional species using HMMs

Owing to the stringency of the *n*-way reciprocal-best blastp hit ortholog selection algorithm, the addition of one species to an *n*-way reciprocal best blastp search results in the identification of many fewer orthologs than using *n*-1 species. For this reason it is advantageous to perform the *n*-way reciprocal best blastp search using only one representative species for each clade of interest, preferably a species from that clade with a well-annotated genome. To observe the genome localization of (CFR, COW, SRO)-HCA-EMU-RES orthologs in additional species, we constructed a script in the `odp` package, `odp_rbh_to_hmm`, that constructs a hidden Markov model (HMM) of each ortholog to search for the best match in new genomes of interest.

The script performs the following steps: For each of the orthologs in a `.rbh` file, the protein sequences in that ortholog are aligned using MAFFT v7.310[100], and a hidden Markov model (HMM) from the alignment is generated using hmmbuild included with hmmer v3.3.2[101]. For each HMM model, and each target species, the proteins of each species' genome are searched using hmmsearch. All of the HMM results are concatenated on a per-species basis, and then best-matches are identified using a greedy gene assignment of ortholog-to-protein based on the bitscore of all of the HMM searches. Like the *n*-way reciprocal best blastp searches, the HMM search process results in one (or zero) genes per species added to each ortholog. If an HMM returns no hits, or has hits that are better matches to other HMMs, it is possible that no gene will be assigned for that ortholog in that species. This is implemented in the odp script `odp_rbh_to_hmm`.

5.2.6 Identifying instances of fusion-with-mixing in odp

Fusion brings together two sets of genes (ALG1 and ALG2) onto the same chromosome. There are two ways to text for mixing. The simplest designates a fusion as "mixed" if there is any overlap between the range (i.e., chromosome coordinate) of the two sets. This is a permissive test, in that even one gene from ALG1 within the territory spanned by ALG2 will lead to calling the fusion as "mixed." It is implemented as a condition within `odp_genome_rearrangement_simulation`. In the main text, we describe and use a second, more sophisticated test which allows for limited overlap based on simulations of mixing within the chromosome, as further discussed in **Supplementary Information 13**. This more sophisticated test is performed by `odp_rbh_plot_mixing`, but is not currently integrated within genome rearrangement simulations (**Supplementary Information 5.2.7**).

5.2.7 Genome shuffling simulations for hypothesis testing

In our analyses we must consider the possibility that patterns of ALG fusion and mixing could have arisen by chance. To ensure that our patterns of synteny have not arisen by chance, we devised a genome shuffling hypothesis test. The test described below is implemented in the program `odp_genome_rearrangement_simulation` using range overlap as described above in **Supplementary Information 5.2.6**).

In this hypothesis test, the user selects a quartet of species. There must be one known outgroup species, and three species (A, B, C) for which the user wishes to resolve the tree topology (**Extended Data Fig. 4, Supplementary Fig. 5.1**). There are three possible trees given the three species in the polytomy, and the program looks for evidence supporting each hypothesis (**Supplementary Fig. 5.1**) and performs genome shuffling simulations to determine if the data could be explained by a highly derived genome state.

The program takes as input a `.rbh` files of the ALGs as described in the previous sections, and uses that file to identify ALGs that participate in fusion-with-mixing events. If the fusion-with-mixing event supports one of the three possible topologies, that event and the number of genes in that event are scored as evidence.

At this stage, the program has tallied the evidence for each hypothesis based on the genomes of the four species that were provided. The program now tests the null hypothesis for each tree topology: that the fusion-with-mixing events grouping two of the clades together can be explained by random chance. The program tests the null hypothesis in four separate trials. Each trial shuffles the protein coordinates of the same species tens of thousands of times. Each time the protein coordinates are shuffled, the program tests to see if there are fusion-with-mixing events detected (using the simple range overlap test). Over tens of thousands of iterations the program explores the space of how many fusion-with-mixing events, and how many genes involved in those events, would exist due to random genome configurations of a single species.

After these trials, the false discovery rate is calculated by dividing the number of trials with the same degree of support for the sister-clade hypothesis as the real genomes divided by the total number of trials. For example, if the real genomes have 250 genes that support hypothesis #1, and 50 shuffling trials out of 1000 also had 250 genes that support hypothesis #1, then the false discovery rate is 50/1000, or 0.05. In our experience, the biological signal for fusion-with-mixing events tends to be unequivocally higher than what is seen in shuffled genomes.

odp_genome_rearrangement_simulation

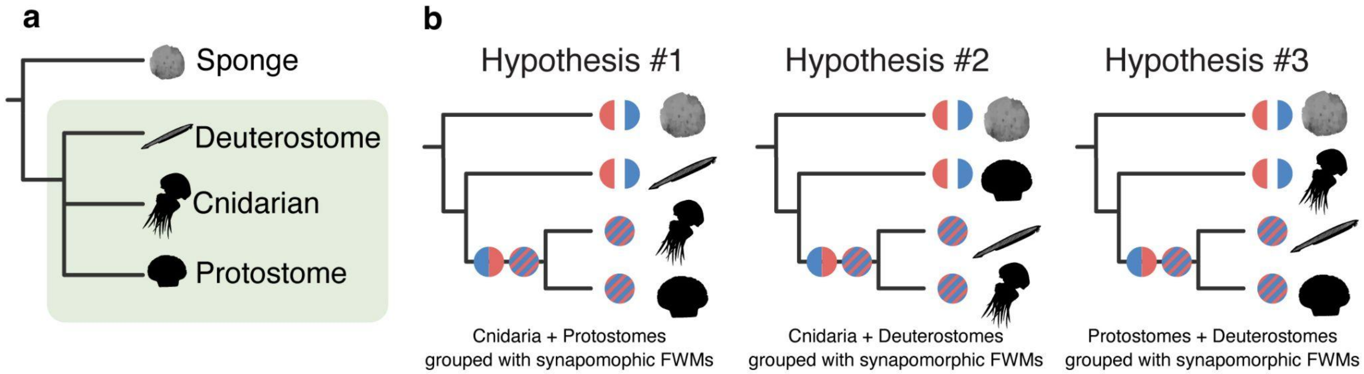

Supplementary Figure 5.1 | **The three hypothesis tests of odp_genome_rearrangement_simulation.**
a. Suppose we have four species: one known outgroup (a sponge), and three species for which we do not know the tree topology (a deuterostome, a cnidarian, and a protostome). **b.** The program `odp_genome_rearrangement_simulation` tests these three hypotheses by searching for synapomorphic fusion-with-mixing events that support each hypothesis. It then performs genome shuffling simulations to calculate if the fusion-with-mixing events can be explained due to random chance.

6 Validating the methodology and ODP software with other clades

6.1 Introduction

As a proof-of-concept, we tested if the *odp* software, in applying the phylogenetic analysis techniques described in this study (**Supplementary Information 4-5**), could recover previously-identified synapomorphic fusion-with-mixing events in various clades[12] For test cases, we selected the previously-identified fusion-with-mixing events unique to spiralians, bilaterians, or cnidarians (depicted in Simakov et al. 2022[12]).

In one test we checked for the presence of the four previously-identified BCnS ALG fusion-with-mixing events that occurred in the ancestor of extant spiralians: L⊗J2, Q⊗H, O1⊗R and O2⊗K[12]. One test sought to recover the four ALG fusion-with-mixing events that occurred in the ancestor of extant Bilateria: R⊗O1, Ea⊗Eb, A1a⊗A1b, and Qbd⊗Qc⊗Qa[12]. The third test sought to identify whether *odp* could recover the six fusion-with-mixing events that occurred in the common ancestor of extant Cnidaria: B1⊗B2, A2⊗N, A1b⊗B3, Qa⊗J1, Qd⊗O2, and Eb⊗F⊗Qb[12]. The species used in these analyses are described below in the methods.

6.2 Methods

We ran the script `odp_nway_rbh` (1) to perform 4-way reciprocal-best diamond blastp searches, (2) to group orthologs into ALGs, (3) to estimate the false discovery rate cutoffs of ALG size for each quartet, (4) to filter the ALGs based on the false discovery rate. We note that by using diamond blastp we are missing some potential orthologs that may have been identified with blastp, but this degree of sensitivity is not necessary for the analyses of the groups described below. The final output of this program is a `.groupby` file that contains the ALGs. The resulting .groupby file was run with the program `odp_genome_rearrangement_simulation` with 100,000 trials of shuffled genomes. This program finds pairs of ALGs that participate in fusion-with-mixing events that can polarize the relationship between three species and an outgroup, and estimates the strength of the biological signal supporting any sister-clade hypothesis over what one would expect from randomized genomes.

First, we tested whether the analysis recovered the bilaterian synapomorphic fusion-with-mixing events we selected the fire jellyfish *Rhopilema esculentum* (RES), the cephalochordate deuterostome *Branchiostoma floridae* (BFL), and the molluscan protostome *Pecten maximus* (PMA). To test whether the analysis recovered the fusion-with-mixing synapomorphies uniting the cnidaria we selected one protosome genome (*P. maximus*), and the genomes of two cnidarians (*R. esculentum* and *H. vulgaris*). Each of these analyses used the genome of the sponge, *E. muelleri* (EMU), as the outgroup.

6.3 Results and Discussion - Validation of the ODP methodology

6.3.1 Recovery of bilaterian fusion-with-mixing synapomorphies

The analysis recovered all three previously-identified fusion-with-mixing events that unite the bilaterians: A1a⊗A1b, Ea⊗Eb, and Qbd⊗Qc⊗Qa, made up of 242 orthologs **(Supplementary Fig. 6.)**.

In the figure shown below, the simulated data were generated by shuffling the *Rhopilema esculentum* genome to see if the support for the bilaterian synapomorphies might be due to the chromosome arrangement of the cnidarian. There were no synapomorphies found uniting the deuterostome and cnidarian, or the protostome and cnidarian. The only support for these two scenarios arose in the randomization trials. The presence of fusion-with-mixing events coupling the bilaterians to the exclusion of the cnidarian and sponge, and the lack of such FWM events in the simulated data, allow us to reject the null hypothesis. (Here mixing is defined by a simple range overlap test, rather than the more accurate test for mixing based on gene order shuffling as described and applied in the main text and **Supplementary Information 13**.) Deuterostome is *B. floridae* (amphioxus) and Protostome is *P. maximus* (scallop).

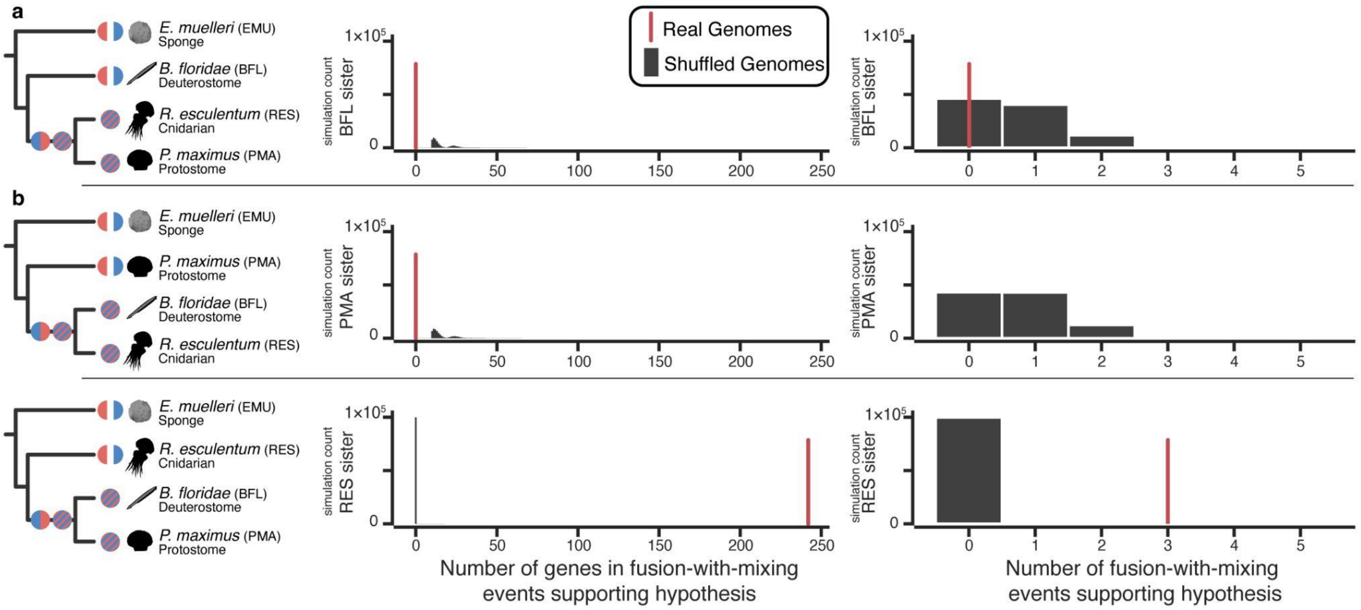

Supplementary Figure 6.1 | **Automated ALG analysis recovers bilaterian synapomorphies.** Every row shows which fusion-with-mixing (FWM) topology is being tested (left), the number of genes in fusion-with-mixing events that support that hypothesis (middle - red vertical line), and the number of fusion-with-mixing events that support that hypothesis (right - red vertical line). The gray bars are histograms of the same measurement made over 100,000 shuffled *Rhopilema* genomes (RES - labeled Cnidarian). **a.** There were no fusion-with-mixing events grouping the cnidarian and deuterostome. **b.** There were no FWM events grouping the deuterostome and the cnidarian. **c.** There were 242 orthologs in three FWM events grouping the deuterostome and protostome.

6.3.2 Recovery of cnidarian fusion-with-mixing synapomorphies

The analysis recovered five of the six previously-identified fusion-with-mixing events that unite the cnidaria: B1⊗B2, A2⊗N, A1b⊗B3, Qa⊗J1, Eb⊗F⊗Qb, made up of 278 orthologs (**Supplementary Fig. 6.3**). The sixth ancestral cnidarian fusion-with mixing event, Qd⊗O2, was not detected due to the stringencies of the four-way reciprocal best hits ortholog finding process, and the relatively low number of genes in ALG_Qd. (Here mixing is defined by a simple range overlap test, rather than the more accurate test for mixing based on gene order shuffling as described and applied in the main text and **Supplementary Information 13**.)

The simulated data shown below (gray bars) were generated by shuffling the *P. maximus* (PMA - labeled Protostome) genome. Cnidarian 1 is *Hydra* and Cnidarian 2 is jellyfish. The biological data only support fusion-with-mixing synapomorphies grouping these two cnidarian species. The biological signal is far stronger than any of the shuffled genomes seen in the simulations.

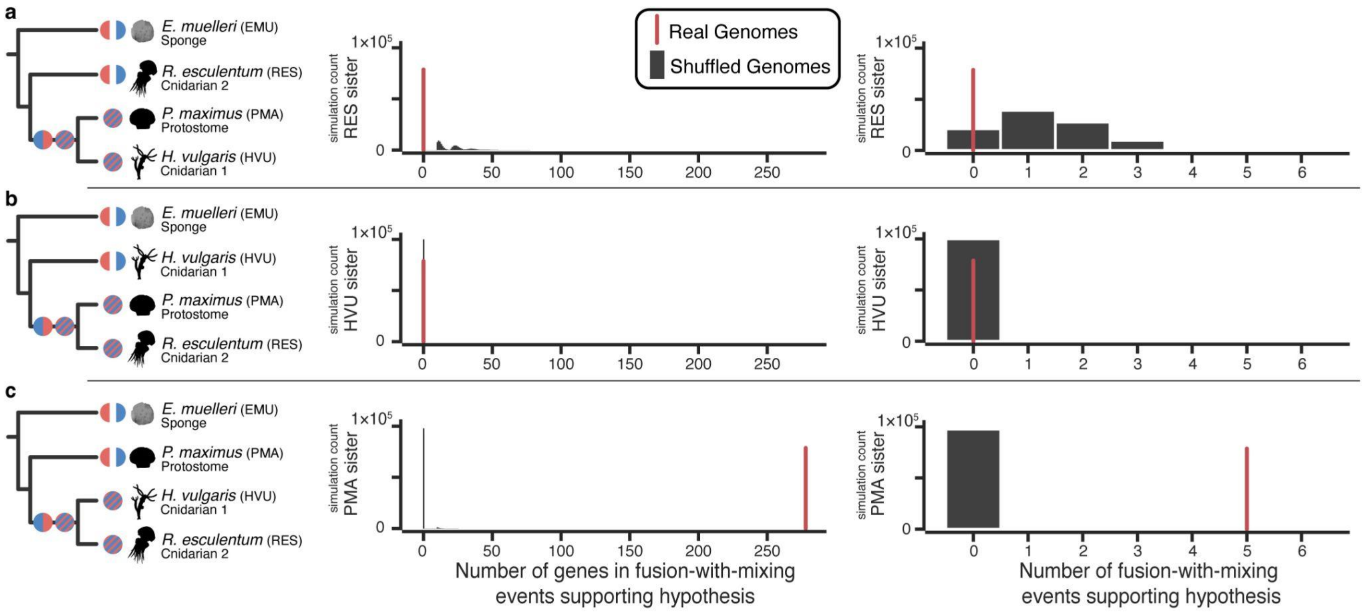

Supplementary Figure 6.2 | **Automated ALG analysis recovers cnidarian synapomorphies**. Every row shows which fusion-with-mixing (FWM) topology is being tested (left), the number of genes in fusion-with-mixing events that support that hypothesis (middle - red vertical line), and the number of fusion-with-mixing events that support that hypothesis (right - red vertical line). The gray bars are histograms of the same measurement made over 100,000 shuffled *P. maximus* (PMA - labeled Protosome) genomes. **a.** There were no fusion-with-mixing events grouping the cnidarian and deuterostome. **b.** There were no FWM events grouping the deuterostome and the cnidarian. **c.** There were 278 orthologs in three FWM events grouping the cnidarians.

7 Macrosynteny analyses of animals and their close unicellular relatives

7.1 Introduction - Macrosynteny Analyses

We compared the genomes of unicellular outgroups to those of diverse animals for which we have chromosome-scale genome assemblies: ctenophores, sponges, cnidarians, and bilaterians. Toward this goal we developed a software package that combines existing techniques[12,57] to identify conserved gene linkage groups. We further developed the software package to analyze the statistical significance of these findings, and test whether alternate hypotheses could explain the findings.

7.2 Results and Discussion - Macrosynteny Analyses

7.2.1 Conservation of ctenophore karyotype

We found that two distantly related ctenophore species (*H. californensis* and *B. microptera*) shared a conserved karyotype of 13 chromosomes despite diverging 160-260 million years ago[10]. The 13 chromosomes in *H. californensis* and B. microptera are in one-to-one correspondence (**Fig. 1d, Extended Data Fig. 2a**). While the four distantly related demosponges (*E. muelleri*[37], the cladorhizid sponge, *C. reniformis*[39,99], *P. ficiformis*[39,99]) exhibit residual gene order colinearity (**Fig. 1d, Extended Data Fig. 2f-h**), the two ctenophores show no appreciable gene order conservation, which indicates extensive intra-chromosomal rearrangement (**Fig. 1d, Extended Data Fig. 2a**). The one-to-one relationship implies that there have been no chromosome fusion or fission events since the two ctenophore lineages diverged from one another. We found extensive gene order rearrangement even between *H. californensis*[40] and scaffolds of the closely related *Pleurobrachia bachei*[5,125], which diverged only 10-40 million years ago[10]. These results suggest that while gene order rearrangement proceeds rapidly in ctenophores, exchange between chromosomes has not occurred. Such conservation of synteny without conservation of gene order is well-known within drosophilids [29] and is common in comparisons across multiple animal lineages[12].

These results indicate that the chromosomal organization of the *Hormiphora* and *Bolinopsis* genomes represents that of a 'typical' ctenophore genome, and that findings in one ctenophore species may be an adequate proxy for analyzing any lobate ctenophore genome. Due to the completeness of the *H. californensis* genome relative to other ctenophore genomes, and because all sequenced ctenophores share 13 homologous chromosomes, we have chosen to use *Hormiphora* as the model ctenophore for comparative analyses described here and in the main text.

7.2.2 Conservation of the demosponge karyotype

Deeply conserved synteny of BCnS chromosomes strongly suggests that other sponges will show extensive chromosome-scale synteny with *E. muelleri* and diverse cnidarians and bilaterians. In order to test this prediction we assembled both haplotypes, to chromosome-scale, of the 1n=18 genome of the bioluminescent carnivorous deep-sea poecilosclerid demosponge *Cladorhiza* sp. (CLA), whose lineage diverged from the freshwater spongillid *Ephydatia* lineage diverged between 350 Mya - 458 Mya[38].

The cladorhizid sponge has n=18 chromosomes, and *Ephydatia* has n=24. As anticipated by the broader conservation of chromosome-scale synteny across BCnS group, we find extensive chromosome-scale conservation of synteny between sponges (*Cladorhiza* sp., *E. muelleri*, *C. reniformis* (NCBI assembly GCA_947172415.1)[39,99], and *P. ficiformis* (NCBI assembly GCA_947044365.1)[39,99]), bilaterians, and cnidarians (**Fig. 1c, Extended Data Fig. 2f-m**). Ten of the chromosomes in each species had one-to-one homologous chromosomes in the other species, with no detectable fusions or fissions on those ten chromosomes in either species (**Supplementary Tab. 2.1**). The remaining 14 chromosomes in *Ephydatia* were split and fused into the 8 remaining cladorhizid sponge chromosomes.

In general, the gene order in the homologous regions were much less mixed than we observed in the homologous ctenophore chromosomes, suggesting a slower inversion rate over time in demosponges.

The fused chromosomes in the cladorhizid sponge were clearly homologous to the ALGs found using *Ephydatia*, *Hydra*, and *Branchiostoma*[12]. While this is by no means a broad sampling of sponge diversity, the conservation of the 29 ALGs in clearly partitioned chromosome fragments over at least 350 million years of divergence is evidence that, from a synteny perspective, both the *Ephydatia* and cladorhizid genomes can be taken as representatives of typical demosponge genomes.

7.2.3 Conservation of chromosomal-scale linkage between sponges, cnidarians, and bilaterians

The chromosome-scale syntenies of sponges, cnidarians, and bilaterians have already been shown to be highly conserved over deep evolutionary time [12], and genomes in these three clades can be described as mixtures of 29 constitutive ALGs spread across the chromosomes, barring cases of extreme rearrangements like *C. elegans* or *D. melanogaster*. We recovered the high degree of conservation between sponges, cnidarians, and bilaterians using `odp`, which was newly developed for this publication (**Fig. 1d, Extended Data Fig. 2**).

7.2.4 Rearranged karyotype in ctenophores relative to other animals

Comparisons between ctenophores and other animals revealed complex patterns of both conserved and disrupted synteny (**Fig. 1d, Extended Data Fig. 2**). We found that while some ancestral BCnS linkage groups are intact on single in ctenophore chromosomes, which extends these ancient syntenies to the metazoan ancestor, other BCnS synteny groups are partitioned across two (or more) ctenophore chromosomes. A certain degree of disruption by fusion is to be expected, since BCnS chromosomes can be expressed as combinations of 29 ancestral linkage groups (BCnS-ALGs[12]) while ctenophores have only n=13 (well-mixed) chromosomes.

In some cases BCnS-ALG syntenies are also conserved in ctenophores, an observation that extends these ancient syntenies (i.e., groups of linked genes) to the most recent common metazoan ancestor, regardless of the detailed branching order of ctenophores, sponges, and other animals (**Supplementary Tab. 7.2)**. In other cases, however, we found groups of genes that consistently occur together on the same chromosomes in bilaterians, cnidarians, and sponges but are partitioned across two (or more) chromosomes in ctenophores (**Figs. 2,3)**.

To quantify these results, we performed a 3-way reciprocal best blastp search between *Hormiphora*, *Ephydatia*, and *Rhopilema*. This yielded 2,623 orthologs conserved between the three species. From these orthologs we identified linkage groups of orthologs that appeared on the same set of chromosomes in the three species, and used genome shuffling simulations in the script odp_rbh_to_groupby to identify linkage groups that were larger than expected by random chance (9 genes or more, $\alpha \leq 9.5\times10^{-3}$)THese . There were 65 linkage groups comprising 1,272 orthologs that allowed us to characterize the relationship between the BCnS ALGs to the ctenophore chromosomes.

The symbol '⊕' has been previously introduced to indicated that whole chromosomes, or chromosome fragments, have fused, then mixed via inversions. Using this notation, we can describe each *Hormiphora* chromosome in terms of the major ALGs that comprise them. From the Oxford Dot Plots of ctenophores and other metazoans (**Extended Data Fig. 2**), colored by orthologs in ALGs, it is immediately clear that ALGs A1a, F, G, L, N, P all appear to be split across at least two ctenophore chromosomes (**Supplementary Tab. 7.2**). To better understand the state of the ALGs in the *Hormiphora* genome, we performed a three-way rbh search using proteins from the HCA-EMU-RES genomes. We found that the *Hormiphora* chromosomes were each composed of major groups of genes from between one and twelve ALGs ($\alpha < 8\times10^{-7}$ for most gene groups) identified in Simakov et al. 2022[12]. Only the ALGs B2, B3, C2, O1, R ($\alpha < 8\times10^{-7}$) and A2 ($\alpha = 1.53\times10^{-3}$) appeared to be present on single *Hormiphora* chromosomes (**Extended Data Tab. 1, Supplementary Tab. 7.2**).

We note that ALGs A1b, B2, and Qabcd have no significantly conserved synteny with *Hormiphora* chromosomes.

The ALGs A1a, B1, C1, D, Ea, F, G, H, J2, L, M, N, and P have significantly conserved synteny ($\alpha < 8\times10^{-7}$) to two Hormiphora chromosomes. This raised the question of whether the split ALGs found in ctenophores reflect the ancestral state, if they are derived splits in ctenophores, or a mixture of both. To answer this question, we later expanded our search to macrosynteny comparisons between animals and the unicellular outgroup species to animals (**Supplementary Information 8, 9, 10**).

7.2.5 The derived karyotype of hexactinellid sponges

In this study we generated a haplotype-resolved, chromosome-scale genome assembly of an undescribed hexactinellid sponge. We refer to this species here as the "tulip hexactinellid" because of its eponymous shape. See **Supplementary Information 2** and **Extended Data Figure 1** for information on the collection, species identification, and genome assembly of this sponge.

We first compared the genome assembly of the tulip hexactinellid to that of the recently reported genome of the closely-related *Oopsacas minuta* hexactinellid sponge[86]. Both of these hexactinellid sponges are in the order Lyssacinosida, however the age of the crown group of this order is not known. With Oxford dot plots based on protein orthology we found that these two genomes share a high degree of gene colinearity (**Supplementary Fig 2.1**). In some cases, single *Oopsacas* scaffolds corresponded to single tulip hexactinellid chromosome-scale scaffolds. These results suggest that genomes of these species likely represent a typical genome of the species in this order of hexactinellid sponges. Given that the tulip hexactinellid genome assembly is chromosome-scale, we limited further analyses to this species. It will be necessary to sequence genomes from a broader taxonomic sampling of hexactinellid sponges to determine whether the *Oopsacas* and tulip sponge genomes also represent typical Hexactinellida genome organization.

Oxford dot plots of the tulip hexactinellid and *Ephydatia* genome assemblies revealed that the lyssacinosid hexactinellid genomes are highly rearranged relative to demosponges (**Supplementary Fig. 2.1, Extended Data Fig. 3**). Fisher's exact test revealed that only BCnS ALGs A1a, Qa, R, D, O2, H, Eb, I, J2, O1, and B1 retain significant conservation on single chromosomes. Given the extensive conservation

of the BCnS ALGs in demosponges, and in other animals, the highly rearranged state of the lyssacinosid hexactinellid genomes appears to be the result of lineage-specific genome tectonic rearrangements. In later sections of the Supplementary Information we will discuss specific findings related to the BCnS ALG A1a.

The BCnS ALGs Ea and G, whose mixed states in bilaterians, cnidarians, and placozoans may be parahoxozoan synapomorphies, were not among the eleven ALGs significantly conserved on single chromosomes in the hexactinellid sponge genome assemblies. Genes from ALG_G and ALG_Ea were distributed across multiple chromosomes, often with orthologs from the _x and _y components present on single chromosomes. Despite the presence of _x and _y orthologs on the same genomes, the genes are scattered across so many chromosomes that it is not possible to tell if these groups underwent synapomorphic fusions in the stem lineage leading to extant sponges, or whether the _x and _y components of ALGs Ea and G became mixed through the extensive derived rearrangements found in the lyssacinosid glass sponges. Therefore, it is necessary to sequence the genomes of more sponge species, including from the Calcarea and Homoscleromorpha, in order to clarify the evolutionary history of sponges and the ALGs Ea and G.

7.2.6 A1a_x and A1a_y exist on separate chromosomes in lyssacinosid glass sponges

The ancestral metazoan linkage groups ALG_A1a_x and A1a_y are found on separate chromosomes in the unicellular outgroups COW and SRO and the ctenophores, but are fused and mixed in bilaterians and cnidarians. Together they comprise ALG_A1a. In the two demosponges we find that A1a_x and A1a_y are collocated on the same chromosome in each species (EMU19 and CLA15, respectively), but have limited overlap on these chromosomes and appear to be unmixed (see **Supplementary Information 13**).

In the lyssacinosid sponge, however, ALG_A1a_x and A1a_y are found on separate chromosome-scale scaffolds (**Extended Data Fig. 3**). Since in the demosponges CLA and EMU these two linkage groups are fused but likely unmixed, a straightforward interpretation of this finding (assuming sponge monophyly[8,10]) is that (1) the fusion of A1a_x and _y arose on the sponge+bilaterian+cnidarian stem lineage by a Robertsonian fusion, producing a biarmed chromosome that was initially resistant to mixing across the centromere; (2) a Robertonian fission in the hexactinellid lineage broke the fusion chromosome at the centromere and restored the ancestral state; (3) on the bilaterian+cnidarian stem lineage the fusion chromosome became mixed. (Robertsonian fissions have been described, e,g, in elephants[126], and shrews[127,128]). Alternately, the A1a_x and A1a_y fusion occurred convergently in demosponges and on the bilaterian+cnidarian stem lineage. Finally, sponges could be paraphyletic, with hexactinellids sister to the demosponge+bilaterian+cnidarian clade. Since fusion-without-mixing is a reversible change, however, we cannot conclude anything about sponge mono- or paraphyly based on synteny alone. Recent sequence-based analyses, however, strongly support sponge monophyly[8,10]. None of these scenarios, however, affect support for ctenophores as sister to all other animals, since the A1a fusion is one of seven shared derived fusion that unite sponges, bilaterians, and cnidarians to the exclusion of ctenophores (main **Figs. 3, 4**).

7.2.7 Karyotype of the choanoflagellate *Salpingoeca* compared to animals

Oxford dot plots of *Salpingoeca* versus animals revealed, perhaps expectedly, highly rearranged chromosomes between *Salpingoeca* and all animal species. However, one unexpected finding was that there were still regions of highly conserved synteny between some portions of *Salpingoeca* chromosomes

and portions of animal chromosomes, including ctenophores, cnidarians, sponges, and bilaterians (**Extended Data Fig. 7**).

Most *Salpingoeca* chromosomes appear to have macrosynteny with two or more chromosomes in metazoan species, and many ALGs appear to be split across multiple *Salpingoeca* chromosomes (**Extended Data Fig. 7**). This is similar to the splitting pattern found when comparing ctenophore chromosomes to ALGs (**Supplementary Tab. 7.2**) and ctenophore chromosomes to other metazoan clades (**Fig. 1, Extended Data Fig. 2b-e**).

However, this analysis does not directly answer whether the ALG splits found in *Salpingoeca* and ctenophores are homologous and ancestral linked groups of genes, or if the ALG splits found in both species' genomes use unrelated genes. This is addressed in later analyses that simultaneously compare multiple metazoan species and one unicellular outgroup species.

7.2.8 Karyotype of the filasterean amoeba *Capsaspora* compared to animals

Oxford dot plots of *Capsaspora* versus animals, and versus ALGs, showed that, despite at least over 800 million years of divergence[50], there were many remaining regions of macrosynteny (**Extended Data Figs. 6,7**). We also note that regions of macrosynteny between *Capsaspora* and animal chromosomes typically correspond to single arms of *Capsaspora* chromosomes. For example, one arm of *Capsaspora* chromosome 3 had significantly conserved macrosynteny with RES3 and RES5, while the other arm had significantly conserved macrosynteny with RES17 (**Extended Data Fig. 7**). It appears that the biology of *Capsaspora* chromosomes may have disfavored inversions across centromeres since the divergence of filastereans, choanoflagellates, and metazoans.

As in the genomes of the choanoflagellate *Salpingoeca* and the ctenophores, groups of genes orthologous to single ALGs are split across two or more *Capsaspora* chromosomes in significant clusters ($p < 5\times10^{-4}$) (**Extended Data Fig. 7**). Twelve BCnS-ALGs have significant conservation on single *Capsaspora* chromosome arms. Later analyses address whether these splits are related to those found in the genomes of ctenophores and choanoflagellates (**Supplementary Information 8, 9, 10**).

7.2.9 Karyotype of the ichthyosporean *Creolimax* compared to animals

Dot plots of the ichthyosporean *Creolimax fragrantissima* genome compared to animal genomes revealed little to no detectable conserved synteny (**Supplementary Fig. 7.2**). Given the conservation between the *Capsaspora*-metazoan genomes, possible explanations for the high degree of rearrangement between *Creolimax* and animals are that either (a) the ancestor of all Holozoa shared groups of linked genes identifiable in extant Filozoa, but the *Creolimax* genome underwent many branch-specific chromosome inversions, fusions, and fissions to disperse the linked genes across the 26 *Creolimax* chromosomes, or (b) major groups of linked genes arose in the genome of the common ancestor of Filozoans through chromosome rearrangements, and *Creolimax* retains elements of the ancestral chromosome state.

In any case, the divergence time of *Creolimax* and Filozoans is estimated to be over one billion years[50]. Given the loss of synteny, it is impossible to tell which scenario, or what mixture of both, can explain the rearranged genome of *Creolimax*. Because of the lack of syntenic signal between *Creolimax* and animals, we focus on the use of the *Capsaspora* and *Salpingoeca* genomes in subsequent analyses.

7.2.10 Genome comparisons between the unicellular outgroups

In whole-genome comparisons, there were no large regions of conserved synteny between the ichthyosporean *Creolimax* and either *Capsaspora* or *Salpingoeca* (**Supplementary Fig. 7.3**).

Some of the regions of significant synteny found in *Capsaspora*-metazoan comparisons, or *Salpingoeca-metazoan* comparisons, were also found in the comparison of *Capsaspora* and *Salpingoeca*. For example, one putative arm of COW3 has many orthologs on SRO5. Both of these chromosomes have many orthologs on HCA7, EMU19, RES2, PMA3, and the ALG A1a.

7.3 Supplementary Figures

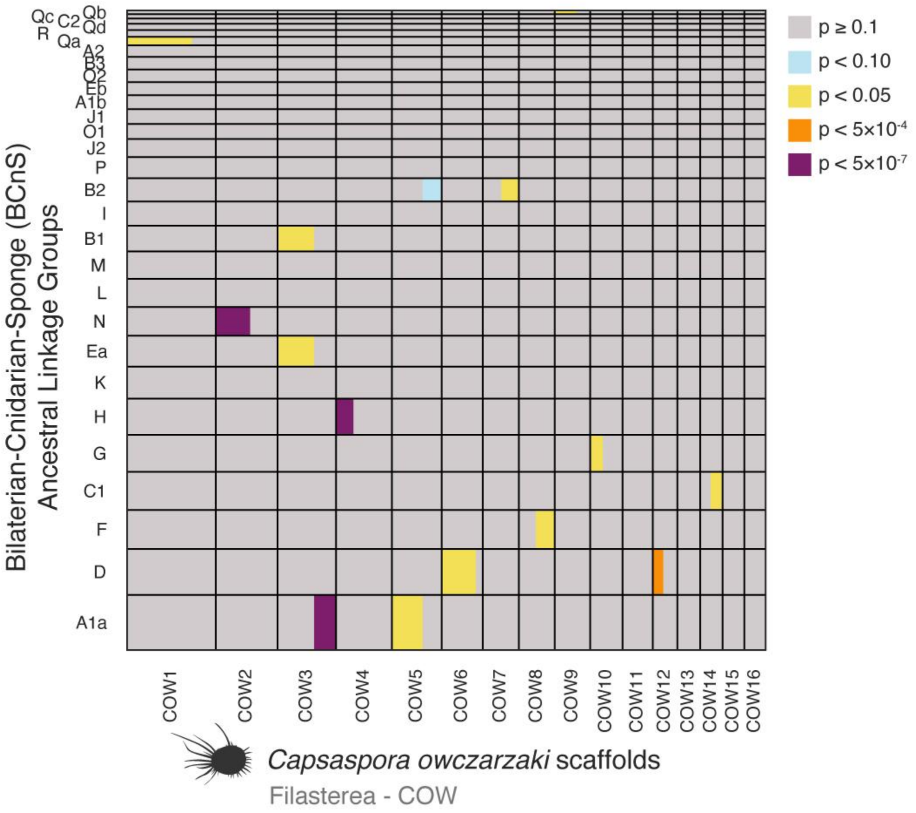

Supplementary Figure 7.1 | ***Capsaspora*-ALG synteny is limited to single *Capsaspora* chromosome arms.** A Bonferroni-corrected one-sided Fisher's Exact test[12] of BCnS-ALGs and *Capsaspora* chromosomes reveal that BCnS-ALGs are conserved on single arms of *Capsaspora* chromosomes. There were no BCnS-ALGs that were identified as being dispersed across the entire length of *Capsaspora* chromosomes. The significant syntenic regions comprise 11 of the 29 BCnS-ALGs.

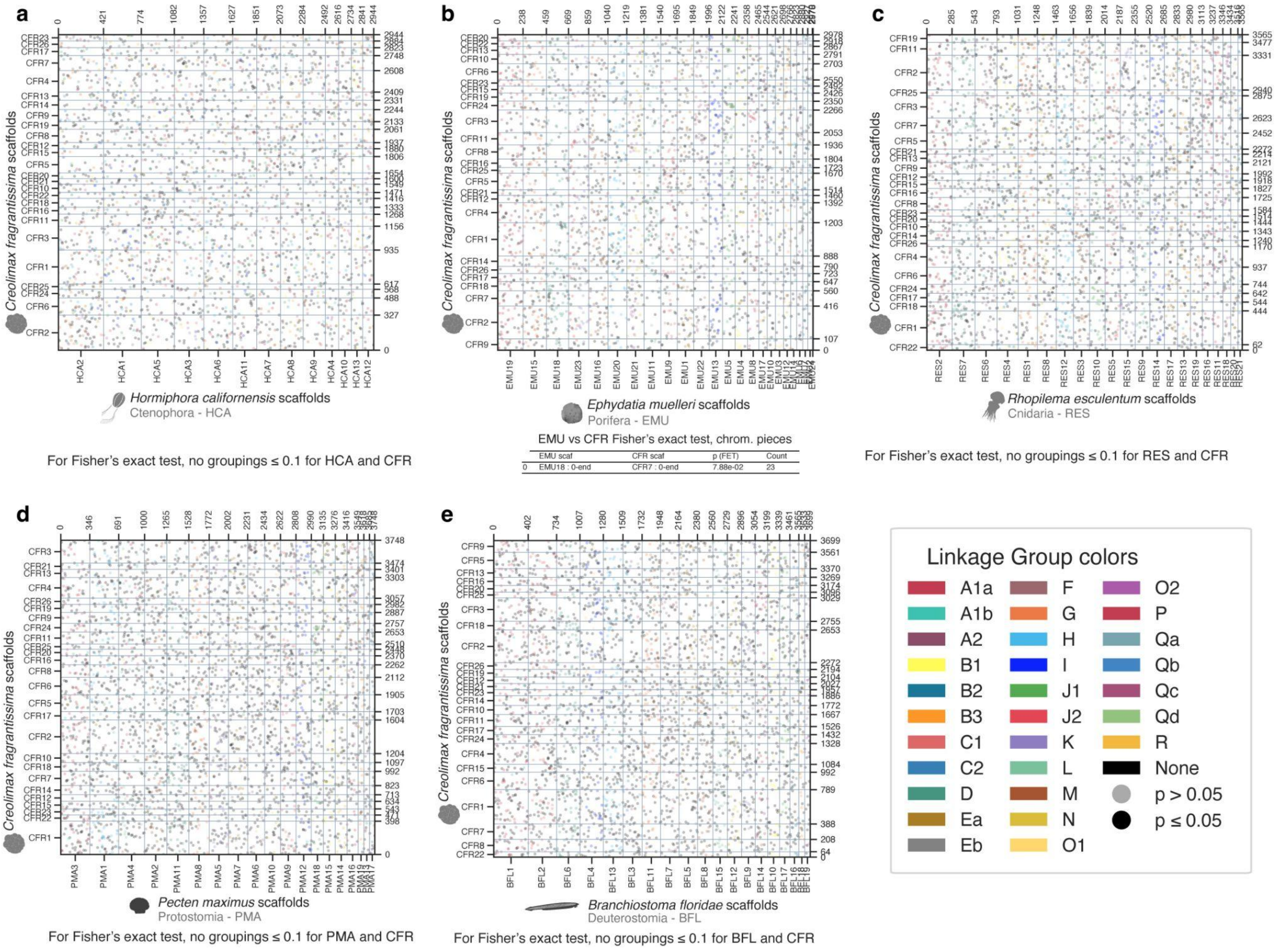

Supplementary Figure 7.2 | **The ichthyosporean *Creolimax* does not exhibit conserved macrosynteny with metazoans.** Two-way reciprocal best hits blast searches between *Creolimax* and animals (**a.-e.**) show that the *Salpingoeca* chromosomes are highly rearranged relative to animal chromosomes, that some regions of synteny remain, and that some ALGs are split across multiple *Salpingoeca* chromosomes. Orthologs are colored based on Simakov et al. 2022[12] BCnS-ALGs, and chromosome pairs with $p > 0.05$ (Bonferroni-corrected one-sided Fisher's exact test[12]) are colored at half opacity. Axis labels show cumulative number of orthologs. Putative centromeres are marked by dotted lines.

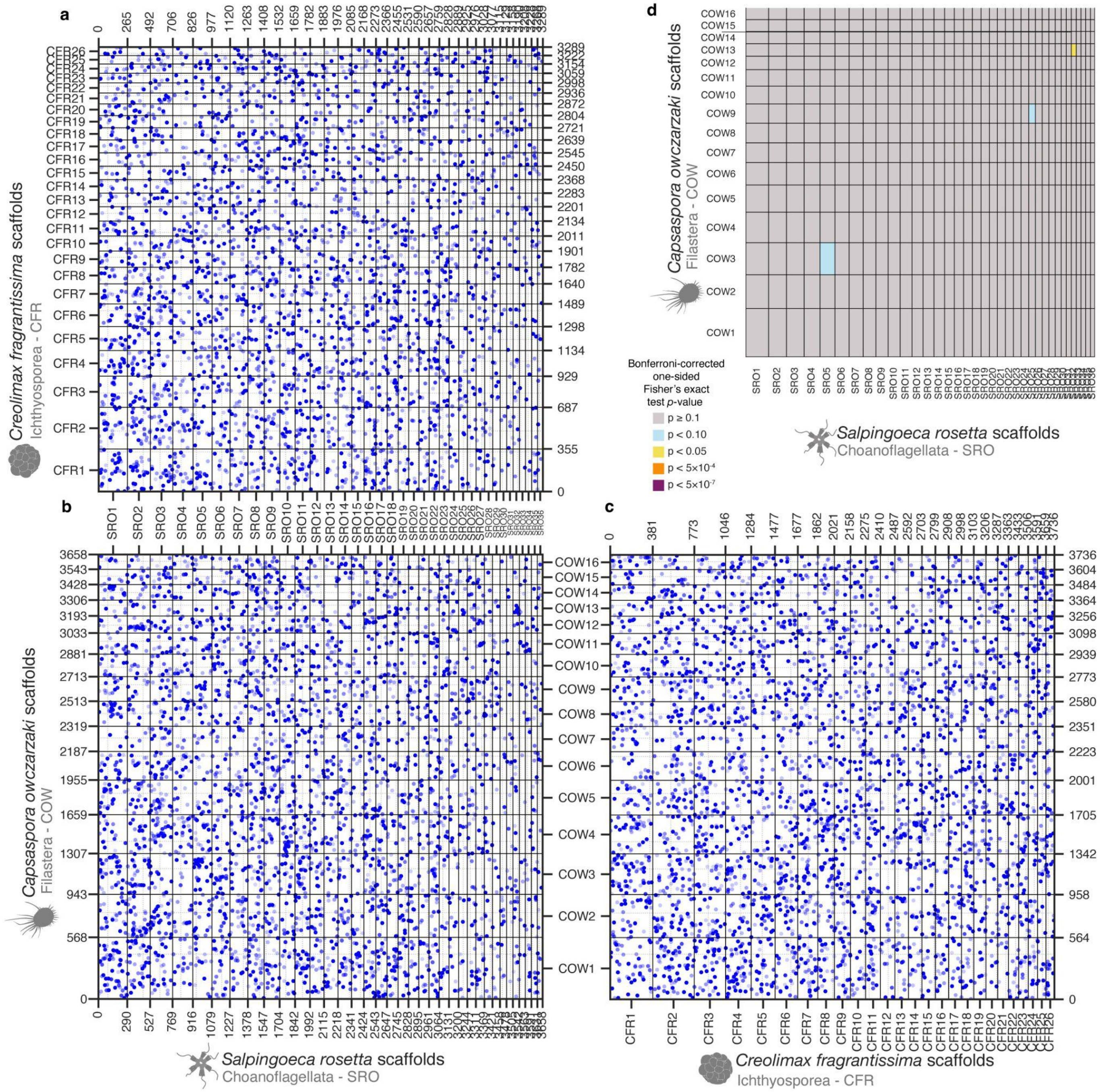

Supplementary Figure 7.3 | **Oxford dot plots comparing unicellular species.** The three unicellular species in this study share little conserved synteny (**a.**-**c.**) with each other. However, the choanoflagellate *Salpingoeca* and the filasterean *Capsaspora* (**d.**) share some macrosyntenic regions that were also conserved in *Salpingoeca*-metazoan (SRO5, SRO25, SRO32) and *Capsaspora*-metazoan (COW3, COW9, COW13) comparisons.

7.4 Supplementary Tables

Clade	Species	3-letter code	Chromosome-scale?	Publication
Ichthyosporea	*Creolimax fragrantissima*	CFR	YES	de Mendoza et al. 2015[44], this study
Filasterea	*Capsaspora owczarzaki*	COW	YES	Suga et al. 2013[43], this study
Choanoflagellata	*Salpingoeca rosetta*	SRO	YES	Fairclough et al. 2013[42], this study
Ctenophora	*Hormiphora californensis*	HCA	YES	Schultz et al. 2021[40]
Ctenophora	*Bolinopsis microptera*	BIN	YES	this study
Porifera	*Ephydatia muelleri*	EMU	YES	Kenny, Francis, et al. 2020[37]
Porifera	Cladorhizid sponge	CLA	YES	this study
Porifera	*Chondrosia reniformis*	CRE	YES	McKenna et al. 2021[39]
Profera	*Petrosia ficiformis*	PFI	YES	McKenna et al. 2021[39]
Placozoa	*Trichoplax adhaerens*	TAD	NO	Srivastava et al. 2008[102]
Cnidaria	*Rhopilema esculentum*	RES	YES	Li et al. 2020[46]
Cnidaria	*Hydra vulgaris*	HVU	YES	Simakov et al. 2022[12]
Cnidaria	*Nematostella vectensis*	NVE	YES	Zimmerman et al. 2020[103]
Bilateria	*Branchiostoma floridae*	BFL	YES	Simakov et al. 2020[57]
Bilateria	*Pecten maximus*	PMA	YES	Kenny, McCarthy, et al. 2020[45]

Supplementary Table 7.1 **| Genomes used in macrosynteny analyses.**

Hormiphora Chrom.		ALGs composing HCA chromosomes, sorted by False Discovery Rate (α)		
		$\alpha < 8\times10^{-7}$	$8\times10^{-7} \leq \alpha \leq 1\times10^{-3}$	$1\times10^{-3} < \alpha \leq 0.01$
HCA1	=	IⓍDⓍB1ⓍJ2ⓍB3	ⓍMⓍA1aⓍH	ⓍF
HCA2	=	GⓍC1ⓍC2ⓍEaⓍDⓍHⓍA1aⓍB2	-	ⓍJ1ⓍIⓍO2ⓍF
HCA3	=	KⓍGⓍDⓍP	ⓍF	ⓍHⓍA1a
HCA4	=	PⓍO1	-	-
HCA5	=	C1ⓍA1a	ⓍFⓍD	ⓍHⓍO2ⓍM
HCA6	=	HⓍNⓍL	-	ⓍDⓍIⓍA1a
HCA7	=	A1aⓍFⓍM	-	-
HCA8	=	EaⓍRⓍM	ⓍDⓍA1a	-
HCA9	=	LⓍM	ⓍD	ⓍIⓍHⓍK
HCA10	=	F	-	ⓍA2
HCA11	=	J1ⓍJ2	-	-
HCA12	=	A1a	-	-
HCA13	=	B1ⓍN	-	-

Supplementary Table 7.2 | ***Hormiphora* chromosomes and ALG arithmetic.** *Hormiphora* chromosomes can be described by the major ALGs that comprise them. ALGs are sorted into three columns based on the false discovery rate of finding a gene linkage group of that size in HCA-EMU-RES orthologs. Within each column, ALGs are sorted, descending, by the number of genes from that ALG in the gene linkage group. ALGs that appear to be split between two or more ctenophore chromosomes are not highlighted. ALGs that do not appear to be split between two ctenophore chromosomes are highlighted pink. ALGs A1b and Qabcd have no significantly conserved synteny with *Hormiphora* chromosomes. The false discovery rates, α, are one-sided false discovery rates calculated from ten million trials of a genome-shuffling permutation test.

8 Identification of gene groups linked since the ancestor of the Filozoa

8.1 Introduction - unicellular-metazoan-ALGs

Pairwise comparisons of bilaterian, sponge, cnidarian, ctenophore, and animal unicellular outgroup species' genomes revealed several major patterns. First, we confirmed previous findings of conservation of synteny between the chromosomes of bilaterians, cnidarians, and sponges[12], and the existence of ancestral linkage groups (ALGs) of genes in the common ancestor of bilaterians, cnidarians, and sponges (BCnS-ALGs) (**Fig. 1, Extended Data Fig. 2**). Our findings revealed that many BCnS-ALGs are partitioned across two or more ctenophore chromosomes (**Fig. 1; Extended Data Figs. 2,8; Supplementary Table 7.2**), and that karyotype is conserved across much of ctenophore diversity (**Extended Data Fig. 2a**). Remarkably we also found that the BCnS-ALGs were often "split" across two or more chromosomes in the genomes of the choanoflagellate *Salpingoeca rosetta* (SRO) and the filasterian amoeba *Capsaspora owczarzaki* (COW), and that these splits corresponded to the splits seen in ctenophores (**Figs. 2,3; Extended Data Figs. 7**).

Using a multi-species approach we identified groups of genes that are both conserved among diverse species across the Filozoa (the clade that includes animals, *Capsapora*, and *Salpingoeca*), and that persist on the same set of chromosomes in those species. Finding such groupings of genes provides evidence for ancestrally-linked groups of genes that were present in the common ancestor to the species in question[32,57]. In addition, finding sets of genes with common fusions in two species to the exclusion of others is phylogenetically informative, if the fusions or fissions are shown to be irreversible through mixing[12].

Toward this goal, we performed reciprocal-best-hit blastp searches between quartets that included the ctenophore *Hormiphora*, the sponge *Ephydatia*, the cnidarian *Rhopilema*, and one of the unicellular outgroup species (*C. fragrantissima*, or *C. owczarzaki*, or *S. rosetta*).

8.2 Results and Discussion - unicellular-metazoan-ALGs

8.2.1 COW-HCA-RES-EMU gene linkage groups - Results and Discussion

Using *Capsaspora* as the outgroup, the four-way reciprocal best blastp search between COW-HCA-RES-EMU yielded 29 groups of genes of between 5 (false discovery rate, $\alpha = 3.2\times10^{-4}$) and 15 genes ($\alpha < 1.0\times10^{-7}$) that are linked together on the same chromosome in the four query species (**Extended Data Tab. 2a**). We estimated the false discovery rate, α, using 10 million permutations of gene indices. Conserved syntenic groups of 8 or more genes never appeared in these simulations, bounding the false discovery rate as $< 10^{-7}$. The reported α values should therefore be considered a conservative upper bound.

Five pairs of these groups follow a pattern in which two groups exist on the same *Ephydatia* and *Rhopilema* chromosomes, but are on separate chromosomes in *Hormiphora* and *Capsaspora*. These five pairs correspond to five BCnS-ALGs identified in Simakov et al. 2022[12], and because the ALGs are partitioned across two gene groups in the COW-HCA-RES-EMU comparison, we refer to the metazoan-ALGs using the suffixes _x and _y. We argue in later sections that these are the gene groups that support the ctenophore-sister hypothesis (A1a_x, A1a_y; C1_x, C1_y, Ea_x, Ea_y; F_x, F_y; G_x, G_y).

Conversely, we did not find any pairs of gene linkage groups that were fused in *Hormiphora* and *Rhopilema* (i.e., on single chromosomes in both genomes) but on separate chromosomes in *Ephydatia* and *Capsaspora*. Thus we find no support for a clade joining ctenophores and cnidarians, with sponges as their sister group. There is also no support for sponge-sister when bilaterians in place of the cnidarian *Rhopilema*, as shown below.

Two additional gene linkage groups that predominantly contain genes from ALG_C1 (designated C1_z1 and C1_z2) are on two separate chromosomes in *Hormiphora* but on one chromosome in the *Capsaspora* genome (**Extended Data Tab. 2a**). This suggests a ctenophore-specific fission of these two gene linkage groups.

Nine gene linkage groups appear to have been on separate chromosomes in the unicellular outgroups, but are merged onto single chromosomes in the common ancestor of the Metazoa (gene linkage groups corresponding to BCnS-ALGs B1, H, I, and K) (**Extended Data Tab. 2a**). These BCnS-ALGs can therefore be interpreted as Metazoan-ALG, and arose either by fusion on the metazoan stem lineage or fission in the *Capsaspora* lineage.

Six gene linkage groups do not appear to have undergone any phylogenetically diagnostic fusions (BCnS-ALGs C2, D, J1, J2, L, and M). These are BCnS-ALGs that are also Metazoan-ALGs, and trace their ancestry back to the common filozoan ancestor.

8.2.2 SRO-HCA-RES-EMU gene linkage groups - Results and Discussion

Similarly, using the choanoflagellate *Salpingoeca* as the outgroup, the four-way reciprocal best blastp search between SRO-HCA-RES-EMU yielded 20 groups of between 5 (false discovery rate, $\alpha = 2.3\times10^{-5}$) and 11 genes ($\alpha < 2.0\times10^{-7}$) that are linked together on the same chromosomes in the four query species (**Extended Data Tab. 2b**). Nine gene groups followed a pattern in which two or three groups exist on the same *Ephydatia* and *Rhopilema* chromosomes, but are on separate chromosomes in *Hormiphora* and *Salpingoeca* (A1a_x, A1a_y, G_x, G_y, L_x, L_y, N_x, and N_y). The gene groups A1a_x, A1a_y, G_x, and G_y also appeared in the COW-HCA-RES-EMU search, and support ctenophore-sister hypothesis as described in the main text.

Conversely, we did not find any pairs of gene linkage groups that were fused on single chromosomes in both *Hormiphora* and *Rhopilema* but split across separate chromosomes in *Ephydatia* and *Salpingoeca*. Thus as with the Capsaspora analysis of **Supplementary Information 8.2.1**, we find no support for a clade joining ctenophores and cnidarians, with sponges as their sister group. There is also no support for sponge-sister when bilaterians in place of the cnidarian *Rhopilema*, as shown below.

Two gene linkage groups appear to have been on separate chromosomes in the unicellular outgroups, but merged onto single chromosomes in at least the common ancestor of the Metazoa (gene linkage groups corresponding to ALG_I). These two groups were also present in the COW-HCA-RES-EMU search.

Nine gene linkage groups do not appear to have undergone any phylogenetically diagnostic fusions (BCnS ALGs B1, C1, C2, D, F, H, K, M, and P). These BCnS-ALGs are therefore also metazan-ALGs whose ancestry can be traced back to the common filozoan ancestor.

8.2.3 CFR-HCA-RES-EMU gene linkage groups - Results and Discussion

Using the icthyosporean *Creolimax* as the outgroup, the four-way reciprocal best blastp search between CFR-HCA-RES-EMU yielded 8 groups of between 5 (false discovery rate, $\alpha = 8.0\times10^{-5}$) and 8 genes ($\alpha < 4.0\times10^{-8}$) that occurred on the same sets of chromosomes in the four query species (**Extended Data Tab. 2c**). None of these gene linkage groups follow a pattern that differentiates between the ctenophore-sister or sponge-sister hypotheses.

Six gene linkage groups appear to have been on separate chromosomes in the unicellular outgroups, but merged onto single chromosomes in at least the common ancestor of the Metazoa (gene linkage groups corresponding to BCnS-ALGs G, H, and I).

Two gene linkage groups do not appear to have undergone any phylogenetically diagnostic fusions (ALG_A1a, K). As before, these are ancient metazoan-ALGs whose ancestry can be traced back to the common filozoan ancestor.

In the analyses of **Supplementary Information 8.2.1-8.2.3** we used the jellyfish *Rhopilema* to represent the cnidarian-bilaterian clade. We have extended these analyses to include bilaterian genomes instead of jellyfish (**Supplementary Information 9, Fig. 3**), and also performed identical analyses using the orthologies identified with OrthoFinder using bilaterian genomes (**Supplementary Information 10, Extended Data Fig. 10**). The results of these additional analyses are consistent with each other and the findings described in the main text.

8.2.4 Merging the (COW, SRO, CFR)-HCA-RES-EMU analyses - Results and Discussion

We used odp_merge_rbh to merge the significant COW-HCA-EMU-RES, CFR-HCA-EMU-RES, and SRO-HCA-EMU-RES gene linkage groups. We merged orthologous groups that shared genes in the three metazoans HCA-EMU-RES. The resulting table consisted of 291 orthologs in 31 groupings. These synteny groups are shown in **Figure 3** and are listed in **Supplementary Data 2, tabs 7 and 8**.

Since the three tables were joined on HCA-EMU-RES, each orthologous gene family contained a gene for all three animals (HCA, EMU, RES). All of the 291 genes occurred in at least one unicellular OG, 81 occurred in at least two OGs, and 9 occurred in all three OGs. Each grouping was composed of between 5 and 29 genes.

Fourteen of the thirty one groupings contain genes that occur on the same chromosomes in *Rhopilema* and *Ephydatia*, but on separate chromosomes in *Hormiphora* and in the unicellular outgroups (ALGs A1a, C1, Ea, F, G, L and N) (**Supplementary Data 2**).

In the case of ALG N, it appears that there has been a fusion of N_x and N_y onto a single chromosome in *Capsaspora owczarzaki*, convergent with the state found in *Ephydatia* and *Rhopilema*. The alternative explanation is that there were two convergent, independent and identical fissions of ALG_N into N_x, and N_y in *Salpingoeca* and *Hormiphora*. Considering that *Capsaspora* has a reduced chromosome number relative to the other unicellular outgroup species (16 vs 26 or 36), it is plausible that this topology could be explained by a convergent fusion. Furthermore, in later analyses we found that N_x and N_y are not fully mixed on *Capsaspora* chromosome 2, suggesting the possibility of a recent fusion.

Five of the thirty one groupings, corresponding to BCnS-ALGs I and K, exist on two or more chromosomes in the unicellular OG species, but are on single chromosomes in the metazoans. This suggests that these ALGs arose on the metazoan stem and are Metazoan-ALGs.

Four of the thirty one groupings, corresponding to ALGs B1 and H, have a fusion pattern suggesting that these BCnS-ALGs arose in the ancestor of the Choanozoa. These are also Metazoan-ALGs.

Two of the groupings, corresponding to a component of ALG_C1, are on single chromosomes in *Capsaspora*, *Rhopilema*, and *Ephydatia*, but are on two chromosomes in *Hormiphora*. This suggests another partial ctenophore-specific split for part of ALG_C1.

The remaining six groupings are conserved among OGs and metazoans, and so do not differentiate between the ctenophore-sister vs. sponge-sister hypotheses. The core genes of ALGs C2, D, J1, J2, M, and P have been colocalized on the same chromosomes since the common ancestor of the Filozoa over 800 million years ago[50].

8.2.5 Alternate *Capsaspora* genome assemblies do not change linkage group results

We note that the above analyses performed on the three alternate *Capsaspora owczarzaki* genome assemblies did not change the groups of linked genes identified in 4-way reciprocal best blastp searches, or the individual *Capsaspora owczarzaki* genes in the linkage groups. No linkage groups of genes were found on the two alternate haplotypes of the fused, or split, configuration of putative *Capsaspora owczarzaki* chromosome 7 (**Extended Data Tab. 1**).

9 Extension of gene linkage groups to other metazoan species

9.1 Introduction - Extension of gene linkage groups

We next asked how the 291 orthologs in 31 groups were distributed across chromosomes in other animal species, and whether the findings based on gene quartets that support the ctenophore-sister hypothesis are supported by the genomes of other animal species. This amounts to a test of our hypotheses from the quartet analyses of **Supplementary Information 8**.

Reciprocal best blastp searches across multiple species is a highly conservative approach to finding orthologous gene families. Each new species added to the analysis reduces the total number of orthologs identified because reciprocal-best blast hit is a stringent criterion, and the number of reciprocal blast conditions that must be satisfied for each additional species *n* scales non-linearly with *n*. Each new species requires n(n – 1)/2 more reciprocal-best blastp hits. To develop a more sensitive method for identifying distant orthologs, we constructed hidden Markov models of each of the 291 orthologous gene families and used them to to identify the most closely related genes in other species beyond the (COW, CFR, SRO)-HCA-RES-EMU set of orthologs.

As shown in **Figure 3** of the main text, we found that the gene linkage groups that support the ctenophore-sister hypothesis, A1a_x, A1a_y, C1_x, C1_y, Ea_x, Ea_y, F_x, F_y, G_x, G_y, L_x, L_y, N_x, and N_y, also occur on single chromosomes in sponge, cnidarian, and bilaterian genomes. We also analyzed the gene linkages on the sub-chromosomal assembly of the placozoan *T. adhaerens* and found evidence for _x and _y genes on the same scaffolds. We analyzed the sub-chromosomal genome of the sponge *A. queenslandica*, but found it to be so fragmented that very few scaffolds had more than one or two genes from the _x and _y components per scaffold, when they occurred.

9.2 Methods - Extension of gene linkage groups

9.2.1 HMM search of 291 orthologs in additional species

For each of the 291 orthologous gene families conserved between unicellular OGs and animals we used the script `odp_rbh_to_hmm` to identify the best matches of the orthologs in additional ctenophore, bilaterian, sponge, and cnidarian species.

We performed HMM-based searches for the 291 orthologous gene families in the genomes of the ctenophore *B. microptera*, the cladorhizid sponge, *T. adhaerens*[102], *H. vulgaris*[12], *N. vectensis*[103], *B. floridae*[57], *P. maximus*[45]. To test for GO enrichment of the sets of orthogroups using PANTHER[104], we also searched for the orthologs in *H. sapiens*.

9.3 Results and Discussion - Extension of gene linkage groups

9.3.1 Conservation of ancestral linkage groups in additional species

After performing the HMM search, the database contained 291 orthologous gene families and the genome coordinates of their members in the core species (CFR, COW, SRO, HCA, EMU, RES), as well as the gene identities and genome coordinates of the best matches in additional animal species. Using the script `odp_rbh_plot_mixing`, we identified the primary chromosomes in the additional species that contained genes from the groups of genes that participate in the phylogenetically informative mixings (A1a, C1, Ea, F, G, L, N). Consistent with our findings in the sponge *Ephydatia* and the cnidarian *Rhopilema*, and consistent with the findings of Simakov et al. 2022[12], the majority of genes in _x and _y

components of the ancestral linkage groups corresponding to ALGs ALG_A1a, C1, Ea, F, G, L, and N were present on single chromosomes in the additional species listed in **Supplementary Table 7.1**. See **Figure 3** in the main text.

9.3.2 Conservation of ancestral linkage groups in *Trichoplax*.

The genome assembly of the placozoan *Trichoplax adhaerens*[102] is not chromosome-scale. However, we found that some of the sub-chromosomal scaffolds of these two genomes contained several genes belonging to both the _x and _y components of the conserved linkage groups, as seen in the chromosome-scale assemblies of the BCnS species.

The sub-chromosomal genome assembly of the placozoan *Trichoplax* shares several BCnS-ALG syntenic synapomorphies with cnidarians[12]. We find that *Trichoplax* scaffolds share at least five of the seven BCnS-stem fusions, with the remaining two fusions unresolved due to the fragmented nature of the current *Trichoplax* scaffolds (**Fig. 3**). The ALGs C1_x and C1_y are present on scaffold TRIADscaffold_3, genes from Ea_x and Ea_y are also on TRIADscaffold_3, genes from Ea_x and Ea_y exist on TRIADscaffold_5, genes from A1a_x and A1a_y exist on scaffold TRIADscaffold_6, and lastly we found G_x and G_y on scaffold TRIADscaffold_9 (**Fig. 3**). These fusion-with-mixing events therefore represent putative syntenic synapomorphies of the bilaterian-cnidarian-placozoan clade, the so-called "Parahoxozoa"[14].

Simakov et al. (2022)[57] united cnidarians and placozoans as sister lineages to the exclusion of bilaterians and sponges based on the putative Ea⊗F fusion-with-mixing shared by placozoans and cnidarians, a grouping also supported by some recent gene trees[55,129,130]. While the current draft genome assemblies of placozoans do not allow to integrate placozoans into our the status of the ancestral pieces of myriazoan ALG_Ea to be determined (**Fig. 3**), it is a strong prediction of our approach that chromosome-scale assemblies of placozoans will show them to be mixed. As stated in the main text of this manuscript, the nesting of placozoans and cnidarians within Myriazoa using synteny rejects both a placozoan-sister-to-other-animals hypothesis[56,131] and the old notion of a Coelenterata clade that would unite ctenophores with cnidarians[53]. Furthermore, since placozoans are nested within Parahoxozoa, homologies between the mouth, gut, and nervous systems of cnidarians and bilaterians imply that placozoans are secondarily flattened, and have lost an ancestral nervous system, rather than representing the ancestral parahoxozoan state.

10 OrthoFinder analysis recovers support for the ctenophore-sister hypothesis

10.1 Introduction

While the approach of ortholog finding using reciprocal best hits (rbh) is generally regarded as a precise and stringent ortholog-finding method[47,124,132,133], one limitation of the rbh approach can be a lower rate of detecting true positive orthology in clades with many duplications[134]. As mentioned before, the stringency does not scale linearly with the number of species *n* used in these searches. There are $n(n-1)/2$ reciprocal-best blastp searches that must be satisfied for *n* species. In this regard, the use of *n*-way reciprocal best blast searches compounds the weakness of the two-species reciprocal best hits methodology.

To overcome this weakness in our *n*-way reciprocal best hit methodology, and to test our phylogenetic hypothesis with results from widely-used ortholog-finding software, we generated orthologs with OrthoFinder. OrthoFinder's ortholog-inference algorithm is also based on a reciprocal-best blastp or diamond blastp search strategy, but allows for orthologs to contain more than one gene per species. This enables each "orthogroup" to more accurately reflect the evolutionary history of the proteins in question.

10.2 Methods

10.2.1 OrthoFinder - methods

We performed an OrthoFinder v2.3.7[98] analysis using standard parameters with diamond blastp v0.9.24[81] for the sequence search, and mafft v7.310[100] for protein alignment. We stopped the software after inferring orthogroups (option -og). The species included in the alignment were: the unicellular organisms *C. fragrantissima* (CFR), *C. owczarzaki* (COW), and *S. rosetta* (SRO); the ctenophores *H. californensis* (HCA) and *B. microptera* (BMI); the sponges *E. muelleri* (EMU) and the undescribed cladorhizid (CLA); the cnidarians *H. vulgaris* (HVU), *R. esculentum* (RES), and *N. vectensis* (NVE); the placozoan *T. adhaerens* (TAD); and the bilaterians *P. maximus* (PMA) and *B. floridae* (BFL). See Supplementary Table 7.1 for citations for each of these genomes.

10.2.2 Species quartets from OrthoFinder - methods

We analyzed the OrthoFinder results in species quartets that can be used to distinguish between the ctenophore- and sponge-sister hypotheses; the use of quartets minimizes the impact of missing data due to gene loss and/or missed orthology. Based on OrthoFinder-orthogroups, we generated .rbh files for all combinations of species such that each quartet contained one unicellular species (COW or SRO), one ctenophore species, one sponge species, and one bilaterian or cnidarian species. Cnidarians were represented by *R. esculentum* or *N. vectensis* for consistency with our rbh-based analyses. The placozoan *T. adhaerens* was omitted as its genome is not chromosome-scale. Since we are interested in chromosome-scale linkage, we discarded ambiguous orthogroups in which any species had orthogroup members on multiple chromosomes. The .rbh files were then analyzed with the `odp_genome_rearrangement_simulation` script that finds groups of genes participating in putative fusion-then-mixing events that can help polarize the phylogenetic relationship of three species given a known outgroup (**Supplementary Information 5.2.7**). We collated these results and compared them to the synteny groups found by the rbh method.

10.3 Results and Discussion

10.3.1 Species quartet analyses support the ctenophore-sister hypothesis

The orthofinder analysis described above yielded 35102 orthogroups. Orthogroups do not always have a gene from every species, so we next selected orthogroups that include a gene for at least one unicellular species, at least one ctenophore, at least one sponge, and at least one cnidarian or bilaterian, i.e., orthogroups that satisfy the condition:

(COW | SRO) & (HCA | BMI) & (EMU | CLA) & (RES | NVE | BFL | PMA)

where genomes are represented by their three letter acronym as described in the main text, "|" means logical "OR", and "&" means logical "AND". There are 32 such four-species quartets. This condition ensures the presence of at least one gene from the outgroup, ctenophore, sponge, and bilaterian/cnidarian clades., This filtering step yielded 3746 orthogroups, or 10.67% of the original 35102 orthogroups. The median number of genes per species per orthogroup was 1.

From these 3746 orthogroups, we generated .rbh files for the 32 possible species quartets that include an outgroup, ctenophore, sponge, and bilaterian/cnidarian species (**Extended Data Fig. 9**). These .rbh files were analyzed with the analysis pipeline `odp_genome_rearrangement_simulation`. Every analysis recovered between one and six fusion-with mixing events that supported ctenophores as the sister clade of other animals. The linkage groups A1a_x and A1a_y appeared the most often and were present in all 32 analyses.

All of the syntenic synapomorphies shown in **Figures 3** and **4** were also found by the OrthoFinder analyses, i.e., A1a_x + A1a_y, C1_x + C1_y, Ea_x + Ea_y, F_x + F_y, G_x + G_y, L_x + L_y, and N_x + N_y. The OrthoFinder analysis also detected a grouping of B1_x + B1_y in a fusion-with-mixing configuration that supports the ctenophore-sister hypothesis. The B1_x+B1_y grouping has the fewest orthologs of any of the linkage group fusions that we identified supporting the ctenophore-sister hypothesis (11 orthologs total in B1_x + B1_y); the small number of genes involved explains why this was not detected by the more conservative reciprocal best hit method. Altogether there were 146 OrthoFinder-orthogroups that participated in large linkage groups, were mixed across the chromosomes in sponge, cnidarians, and bilaterians, and were configured in a fusion-with-mixing event supporting ctenophores as the sister clade of all other animals.

The OrthoFinder-based quartet analyses are unanimous in their support of ctenophore-sister, based on the syntenic synapomorphies shown in **Figures 3 and 4** and described in the previous paragraph. Three of the 32 quartets, however, exhibit an unusual distribution of BCnS ALG_H in the cladorhizid sponge genome that merits further discussion, as it demonstrates how to polarize more recent changes in synteny within a clade. Specifically, genes from BCnS ALG_H are split across two chromosomes in the cladorhizid sponge and four chromosomes in *Capsaspora* (**Extended Data Figs. 9c,d**). The manner in which ALG-H is split in the two genomes, however, is not the same (**Extended Data Fig 9c**), suggesting an independent fission in the cladorhizid genome. The independence of this fission is supported by analysis of other sponges, including *E. muelleri,* the stony sponge *Petrosia ficiformis* (three-letter code PFI - NCBI genome accession GCA_947044365.1)[39,99], and the distantly-related *Chondrosia reniformis* (three-letter code CRE - NCBI genome accession GCA_947172415.1)[39,99] (**Extended Data Fig. 9c-h**), all of which possess an intact ALG_H on a single chromosome. Based on phylogenetic relationships of demosponges[38,109] (**Extended Data Fig. 9b**), and the presence of BCnS ALG_H on single chromosomes in the sponges *E. muelleri* (EMU20), *C. reniformis* (CRE4), and *P. ficiformis* (PFI8) the most parsimonious explanation of the evolutionary history of BCnS ALG_H in the demosponges is that it ancestrally existed

on a single chromosome, and became split onto two chromosomes as an autapomorphy in the cladorhizid sponge lineage. Since ALG_H also exists on single chromosomes in all animal clades {the ctenophores (HCA6 and BMI6), the cnidarians (HVU11, NVE13, RES12), and the bilaterians (BFL13, PMA1) and on a single scaffold in the placozoan *T. adhaerens* (Scaffold 2)}, we conclude that ALG_H was syntenic in the metazoan ancestor.

10.3.2 Overlap in gene content between rbh and OrthoFinder analyses

We compared the phylogenetically informative gene families derived from rbh and OrthoFinder analyses, i.e., orthogroups that participate in synteny group fusions defined in the main text. Out of 291 rbh-orthogroups (**Supplementary Information 8,9**), 144 were phylogenetically informative. We searched for these rbh genes in the 3756 Orthofinder-orthogroups remaining after the initial filtering step. We found that 116/144 of the phylogenetically informative rbh-orthogroups (extended with other species in **Supplementary Section 9**) had 100% of their genes present in the most similar OrthoFinder-orthogroup. The remaining 28/144 rbh-orthogroups have between 70%-92% of their genes present in the most similar OrthoFinder orthogroup. This demonstrates that the rbh method produces a robust but conservative set of orthogroups.

11 Detecting conserved macrosynteny between highly rearranged genomes

11.1 Introduction

We have shown, both here and in Simakov et al. 2022[12], that Fisher's exact test can be used to detect conserved synteny between species that have experienced limited large-scale chromosomal changes since diverging from their common ancestor. For example, the chromosomes of the ctenophores *H. californensis* and *B. microptera* are in one-to-one correspondence, with only a few genes translocated between chromosomes since their common ancestor (**Fig 1d**, main text, and **Extended Data Figure 2a**). In these cases, conserved chromosome-scale (i.e., “macro”) synteny is easily detected using Fisher’s exact test, and Bonferroni-corrected *p*-values for significant conservation of synteny between a chromosome pair can fall below python’s underflow limit of approximately 2×10^{-308}. Animals with half a billion years of divergence often share highly conserved synteny[12]. For example, the sponge *E. muelleri* and the jellyfish *R. esculentum* diverged before the Cambrian Era but have chromosomes whose homology is easily detectable by eye with an Oxford dot plot[12]. Bonferroni-corrected *p*-values of Fisher's exact test of conserved synteny between whole chromosomes range between 0.0375 for the smallest conserved segments (sharing 12 orthologs), to 1.72×10^{-199} for an orthologous chromosome pair (sharing 188 orthologs).

While Fisher’s exact test provides a conservative *p*-value, the test can lack power to detect smaller conserved groups of genes when one or both of the genomes in question in a two-species comparison have undergone many rearrangements and interchromosomal transfers. Specifically, we observed regions of conserved macrosynteny between the genomes of *C. owczarzaki* (three-letter code COW) and sponges, cnidarians, and bilaterians (**Extended Data Fig. 7**). Notably, in *C. owczarzaki* the conservation was often limited to single chromosome arms, and the same arms that were conserved between COW and sponges, cnidarians, and bilaterians were also visibly conserved between COW and ctenophores. Despite this consistent conservation across animals, application of Fisher’s exact as described in Simakov[12] test yielded only two significant COW-ctenophore conserved syntenic associations with Bonferroni-corrected *p*-values, 0.0124 and 2.15×10^{-7} (**Extended Data Fig. 7a**). The fact that these same COW chromosome arms show significant conserved synteny with other animals that coincide with significant

ctenophore-other animal conservation provides further confidence that they represent a signal of ancient conserved synteny (**Extended Data 7b-d**, **Extended Data Figs. 7b-7d**).

Based on these observations we reasoned that an analysis that considered multiple species simultaneously would have greater power to detect conserved syntenies between highly rearranged genomes. Such a method would enhance signal-to-noise by developing a more refined set of syntenic orthologs for consideration using Fisher's exact test. To this end we developed a method based on ortholog network conservation scores that considers a pair of focal genomes (e.g., COW and HCA) in the context of other related genomes.We show that (1) this method does not detect conservation between the most distantly related of our outgroups, ichthyosporean *Creolimax fragrantissima* (CFR), and the ctenophore *Hormiphora californensis* HCA), consistent with visual inspection, but an finds significant conserved syntenies between the closer outgroups *Capsaspora* (COW) and *Salpingoeca* (SRO) that are consistent with those found between these two outgroups and other animals, and between ctenophores and other animals.

11.2 Methods

11.2.1 Interpreting two-species comparisons in a muti-species context

Gene translocation during evolution is a stochastic process[144,145], uninterrupted conserved gene linkages across species are likely relicts of ancient gene linkages[144]. These properties lead us to consider conserved gene linkage across many species as a conservation score to determine whether a given gene pair colocalized on a chromosome reflects an ancestral configuration, or a derived rearrangement.

Consider a chromosome that contains 1000 genes in an ancestral species whose descendants diverge into 26 independently-evolving species $s_{A...Z}$ with a complex evolutionary history. During the course of the chromosome evolution of these species there are many unique and independent heritable gene translocation events. Visualizing the synteny between any two genomes, say of species s_A and s_B, on the tips of that evolutionary tree will show that there have been gene translocations since the divergence of those two genomes from the ancestral state. However, if we look at the position of the genes translocated between $s_{A,B}$ in the genomes of the other 24 extant species $s_{C...Z}$, we may see that those genes tend to exist on the same chromosomes of those 24 species. In this case, we know that the genes translocated between $S_{A,B}$ are conserved on the same chromosome in most other species, and therefore likely share an evolutionary history[144]. This degree-of-conservation is at the gene-gene level, and the degree-of-conservation can also be calculated at the level of gene communities when considering many gene-gene conservations in a network. This concept is represented in **Extended Data Figure 7**, a formal definition of the ortholog network conservation scores follows in **Supplementary Information 11.2.2-11.2.4**, and our findings are presented in **Supplementary Information 11.3**. This program is available in the FigShare repository under the name `orthology_conservation_score.py`.

11.2.2 Ortholog data structure

To perform these analyses, we must first estimate orthogroups between many species using Orthofinder v2.5.4 using blastp. See **Supplementary Information 10** detailing our analyses and species included for the Orthofinder analysis performed for this study.

As we are looking for conservation of gene colocalizations of two species based on the gene colocalization in other species, this Orthofinder analysis must include at least three species. The species included in the Orthofinder analysis can be described as a set s.

Below, we will analyze the macrosynteny of species s_1 and s_2 in the context of the orthologs' conservation in the species $[s_3, \ldots, s_k]$.

$$s = [s_1, s_2, \ldots, s_k] \text{ where } |\, s \,| \geq 3 \tag{1}$$

Every species s_k has the additional property of a list of chromosome/scaffold IDs. Let p be the number of scaffolds in the genome assembly and annotation for a given species $s_k \in s$.

$$\beta(s_k) = [x_1, \ldots, x_p] \tag{2}$$

Let us represent each orthogroup resulting from an Orthofinder analysis, or from another method of orthology finding, as a node V_i in the set of nodes V.

$$V = \{v\}_{v \in \text{ in the ortholog-finding analysis of } \forall s_k \in s} \tag{3}$$

Each orthogroup $V_i \in V$ will have m genes for a given species s_k, and each gene has a chromosome x_m on which it resides. We access the chromosomes on which each gene resides with the function:

$$\kappa(V_i, s_k) = [x_1, \ldots, x_m] \tag{4}$$

In this analysis, we are comparing the macrosynteny of species s_1 and s_2. To avoid chromosome misidentifications, for each species s_i we select only orthogroups $V_i \in V$ that contain one gene, or contain multiple genes only on one chromosome. We define a function returning boolean values indicating whether the gene(s) in each ortholog V_i exist(s) on single chromosomes in species s_i.

$$\sigma(V_i, s_k) = \begin{cases} True & \text{when } |\, \{x \,|\, x \in \kappa(V_i, s_k)\,\} \,| = 1 \\ False & \text{otherwise} \end{cases} \tag{5}$$

Moving forward, we limit our analyses to orthogroups $V_i \in V$ if and only if the function $\sigma(V_i, s_k)$ is satisfied for both species s_1 and s_2. We call this subset of orthogroups W.

$$W = \{V_i \in V \,|\, \sigma(V_i, s_1) \text{ and } \sigma(V_i, s_2)\} \tag{6}$$

11.2.3 Conservation score

The set W contains orthogroups that contain genes that only exist on single chromosomes in both species s_1 and s_2. In the first step toward constructing a pairwise conservation score we select pairs of orthogroups that exist on the same pairs of chromosomes p and q in the species s_1 and s_2.

$$W_{p,q} = \{W_i \in W \mid \{\kappa(W_i, s_1)\} = p \text{ and } \{\kappa(W_i, s_2)\} = q\,\} \tag{7}$$

We also define a set of edges $E_{p,q}$ that each ortholog is connected to in $W_{p,q}$. The set of all edges in the graph is defined by E.

$$E_{p,q} = \{(u, v) \in W_{p,q} \times W_{p,q} | u \neq v\} \tag{8}$$

$$E = \bigcup\{\{e \in E_{p,q}\} p \in \beta(s_1), q \in \beta(s_2)\} \tag{9}$$

We designate an identity function to test whether two chromosome IDs x_i and x_j are the same. For example, we later use this function to see if two orthogroups W_i and W_j exist on the same chromosome in species s_k.

$$\delta(x_i, x_j) = \begin{cases} 1 & \text{when } x_i = x_j \\ 0 & \text{otherwise} \end{cases} \tag{10}$$

For a given edge (W_i, W_j) in set of edges $E_{p,q}$, and a species s_k in $s_{3,\ldots,|s|}$ we quantify whether the orthologs W_i and W_j, which are present on the same chromosomes in species s_1 and s_2, are present on the same chromosome in species s_k. We define this value below to be returned by the function $c(W_i, W_j, s_k)$.

To quantify the changes in chromosome position that may have occurred during the evolutionary history of orthogroups, and because orthogroups sometimes have multiple sequences for a single species, we calculate the value for $c(W_i, W_j, s_k)$ in the range $[0, 1]$. The value $c(W_i, W_j, s_k) = 0$ means that none of the sequences in the orthogroups W_i and W_j exist on the same chromosome in species s_k. Conversely, the value $c(W_i, W_j, s_k) = 1$ means that all of the sequences in the orthogroups W_i and W_j exist on the same chromosome in species s_k.

$$c(W_i, W_j, s_k) = \frac{\Sigma_{m \in \kappa(W_i, s_k)} \Sigma_{n \in \kappa(W_j, s_k)} \delta(m, n)}{|\kappa(W_i, s_k)| \cdot |\kappa(W_j, s_k)|} \tag{11}$$

Because we wish to analyze the conservation of gene organization between species s_1 and s_2 relative to many other species, we calculate the gene pair conservation scores, $c(W_i, W_j, s_k)$, of the species in $[s_3, \cdots, s_k]$. We chose to summarize the results of ortholog-ortholog conservation across many species with a median of those values. Measuring the median of the conservation scores is robust against outliers in a scenario in which the gene pair is conserved on single chromosomes in most, but not all, species in $[s_3, \cdots, s_k]$. Therefore, the expression of the gene colocalization conservation score of two orthogroups W_i and W_j is:

$$C((W_i, W_j)) = \text{median}(\{c(W_i, W_j, s_3), \ldots, c(W_i, W_j, s_k)\}_{k \in \{3,\ldots,|s|\}}) \quad (12)$$

From the measurement of ortholog-ortholog conservation on a single chromosome in many species, we can also estimate a measure of conservation of a single ortholog in the context of all genes located on the same chromosome pair in species s_1 and s_2. This measure gives an approximation of the percent of genes in a single chromosome pair are conserved.

$$C(W_i) = \text{mean}(\ \{\ e \in E \mid W_i \in e\ \}\ ) \quad (13)$$

11.2.4 Significance Testing

The measures $C((W_i, W_j))$ and $C(W_i)$ are the two measures of gene-gene colocalization conservation we noted in earlier sections. Either of these two measures can be now be used in Fisher's exact test to test for the significance of macrosyntenic relationships between two species.

To test for significance for single gene-gene conservation scores, we use a Fisher's exact test on the counts of the the gene-gene conservation scores $C((W_i, W_j))$ greater than or equal to a threshold t. The threshold t is the same range as the conservation scores: $[0, 1]$. The Fisher's exact test table is constructed as shown below. We use the notation $\neg p$ to refer to all components of $\beta(s_1)$ except p. Likewise $\neg q$ refers to all components of $\beta(s_2)$ except q.

	Inside $\beta(s_1)_p$	Outside $\beta(s_1)_p$
Inside $\beta(s_2)_q$	$\lvert\{e \in E_{p,q} \mid C(e) \geq t\}\rvert$	$\lvert\{e \in \bigcup E_{\neg p,q} \mid C(e) \geq t\}\rvert$
Outside $\beta(s_2)_q$	$\lvert\{e \in \bigcup E_{p,\neg q} \mid C(e) \geq t\}\rvert$	$\lvert\{e \in \bigcup E_{\neg p,\neg q} \mid C(e) \geq t\}\rvert$

The resulting Fisher's exact test is Bonferroni-corrected by multiplying the p-value by $|\beta(s_1)| \cdot |\beta(s_2)|$. This method of significance testing may be highly sensitive in that it only rewards conservation across many species.

Measuring Fisher's exact test on the ortholog-network-level conservation $C(W_i)$ measures gene conservation in the context of the entire chromosome, and is therefore more sensitive to derived chromosome breaks in s_1 or s_2.

We performed Fisher's exact test at the ortholog-network-level with the following calculation, also performing a Bonferroni correction by multiplying the p-value by $|\beta(s_1)| \cdot |\beta(s_2)|$:

	Inside $\beta(s_1)_p$	Outside $\beta(s_1)_p$
Inside $\beta(s_2)_q$	$\lvert\{w \in W_{p,q} \mid C(w) \geq t\}\rvert$	$\lvert\{w \in \bigcup W_{\neg p,q} \mid C(w) \geq t\}\rvert$
Outside $\beta(s_2)_q$	$\lvert\{w \in \bigcup W_{p,\neg q} \mid C(w) \geq t\}\rvert$	$\lvert\{w \in \bigcup W_{\neg p,\neg q} \mid C(w) \geq t\}\rvert$

11.3 Results and Discussion

We performed the ortholog network conservation score method described above to test for significant conserved synteny between the outgroups *Capsaspora* and *Salpingoeca,* and the ctenophore *Hormiphora,* using all other genomes for network context. The orthology network was obtained using OrthoFinder as described in **Supplementary Information 10**.

11.3.1 False positives vs the ortholog-ortholog conservation score

To test the methodology we first revisited the comparison between the unicellular icththyosporean *Creolimax fragrantissima* (CFR) and the ctenophore HCA. Based on visual inspection and other analyses we anticipate minimal, if any, conserved synteny. Thus the CFR-HCA comparison (in the network context of other animals) is a test of whether the our orthology network conservation score method produces false positive conserved syntenies. We applied Fisher's exact test to (1) graph edges of the ortholog network (**equation 12** above), and (2) the nodes of the ortholog network graph (**equation 13**) with varying cutoffs. For this test we compared the chromosome arms of CFR to the chromosome arms of HCA. Since various other custom analyses detected no conserved synteny between CFR and HCA, we reasoned that any conserved synteny identified by our ortholog network conservation method would be a likely false positive.

Supplementary Table 11.1 shows the number of synteny groups that are significant using Bonferroni-corrected Fisher's exact test p-value ≤ 0.05 (column labeled BC-FET) as a function of network cutoff t. While low (i.e., permissive) values of the ortholog-ortholog network cutoffs yield presumptive false positive signals, these decrease with t so that when $t \geq 0.75$ there are no false positives remaining. This is as expected, since the cutoff t acts on the median of the single-species conservation scores for each ortholog pair (**equation 11**), and a t closer to 1 means that the ortholog pair is present on the same chromosome in the majority of species in the OrthoFinder results. Further development of this metric might focus on an automated method to estimate appropriate values for t for different datasets.

Cutoff t	Number of BC-FET significant groups
0	50
0.05	36
0.1	14
0.15	11
0.2	5
0.25	4
0.3	3
0.4	4
0.5	2
0.6	3
0.7	1
0.8	0
0.9	0
1.0	0

Supplementary Table 11.1 | **CFR-HCA ortholog-ortholog conservation score significant groupings.** This table shows the number of significant CFR-chromosome-arm/HCA-chromosome interactions there were when performing Fisher's exact test on the ortholog-ortholog-conservation scores (**equation 12**).

11.3.2 False positives in the ortholog network conservation score

Similarly, we compared CFR to HCA using the node-based ortholog network score C(W). Supplementary Table 11.2 shows the number of synteny groups that are significant using Bonferroni-corrected Fisher's exact test *p*-value ≤ 0.05 (column labeled BC-FET) as a function of network cutoff *t* (**equation 13** above). For this test, we found at most one presumptive false positive over the entire range of *t*. This test retains orthologs that are conserved on the same chromosome in other species with a proportion t of orthologs on the same chromosome-chromosome pair. In other words, this test performs Fisher's exact test only on orthologs that are part of a conserved ortholog linkage group found in other species. The only significant grouping detected by this method is conserved synteny between between HCA5 and the long arm of CFR1.

Comparing **Supplementary Tables 11.1** and **11.2** e we conclude that ortholog network conservation score (**equation 13**) is not subject to the same concerns of false positives as the Fisher's exact test of the ortholog-ortholog conservation score (**equation 12**).

Cutoff *t*	Number of BC-FET significant groups
0	0
0.05	1
0.1	1
0.15	0
0.2	1
0.25	0
0.3	0
0.4	1
0.5	1
0.6	0
0.7	0
0.8	1
0.9	0
1.0	0

Supplementary Table 11.2 | **CFR-HCA ortholog network conservation score significant groupings.** This table shows the number of significant CFR-chromosome-arm/HCA-chromosome interactions there were when performing Fisher's exact test on the ortholog network scores (**equation 13**).

11.3.3 Sensitivity compared with Fisher's exact test results

We next tested the ortholog network methods for their ability to identify true positives – that is chromosome syntenies that were previously detected with Fisher's exact test on pairwise comparisons without filtering by conservation score. We considered the pairwise comparison of the unicellular outgroup *C. owczarzaki* (COW) and the fire jellyfish *R. esculentum* (RES). Bonferroni-corrected one-sided Fisher's exact test identified 19 significant macrosyntenies between the RES chromosomes and COW chromosome arms (**Supplementary Fig. 11.1**). We asked how many of these groupings were recovered with different t values for the both the ortholog-ortholog conservation score and ortholog network conservation score methods described above.

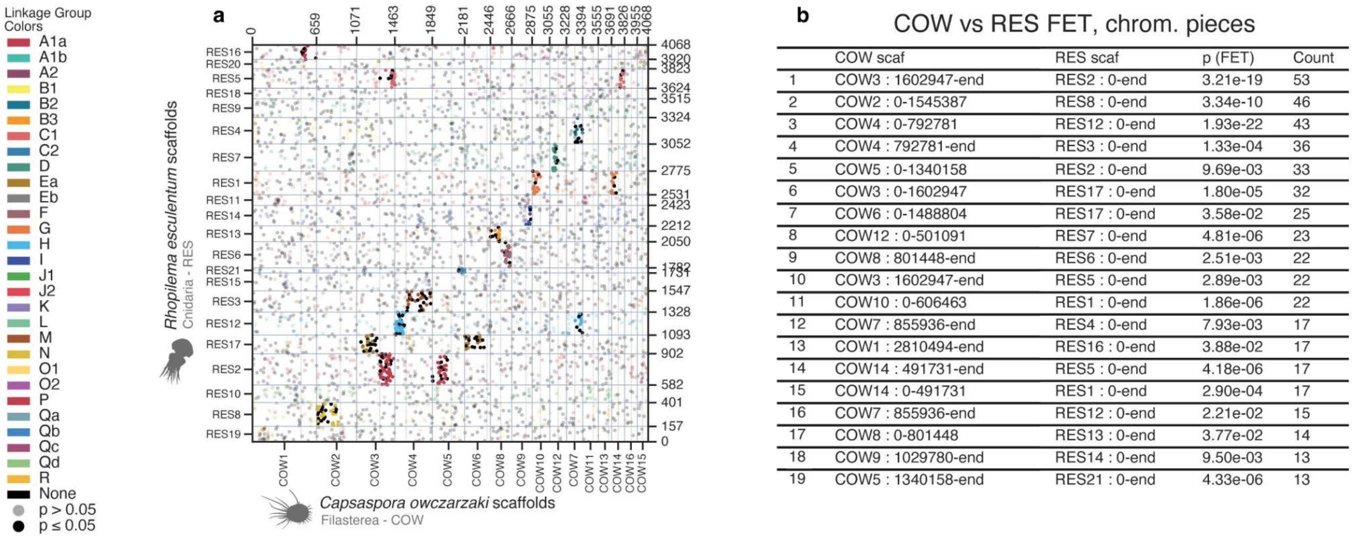

b

COW vs RES FET, chrom. pieces

	COW scaf	RES scaf	p (FET)	Count
1	COW3 : 1602947-end	RES2 : 0-end	3.21e-19	53
2	COW2 : 0-1545387	RES8 : 0-end	3.34e-10	46
3	COW4 : 0-792781	RES12 : 0-end	1.93e-22	43
4	COW4 : 792781-end	RES3 : 0-end	1.33e-04	36
5	COW5 : 0-1340158	RES2 : 0-end	9.69e-03	33
6	COW3 : 0-1602947	RES17 : 0-end	1.80e-05	32
7	COW6 : 0-1488804	RES17 : 0-end	3.58e-02	25
8	COW12 : 0-501091	RES7 : 0-end	4.81e-06	23
9	COW8 : 801448-end	RES6 : 0-end	2.51e-03	22
10	COW3 : 1602947-end	RES5 : 0-end	2.89e-03	22
11	COW10 : 0-606463	RES1 : 0-end	1.86e-06	22
12	COW7 : 855936-end	RES4 : 0-end	7.93e-03	17
13	COW1 : 2810494-end	RES16 : 0-end	3.88e-02	17
14	COW14 : 491731-end	RES5 : 0-end	4.18e-06	17
15	COW14 : 0-491731	RES1 : 0-end	2.90e-04	17
16	COW7 : 855936-end	RES12 : 0-end	2.21e-02	15
17	COW8 : 0-801448	RES13 : 0-end	3.77e-02	14
18	COW9 : 1029780-end	RES14 : 0-end	9.50e-03	13
19	COW5 : 1340158-end	RES21 : 0-end	4.33e-06	13

Supplementary Figure 11.1 | **COW-RES unfiltered Fisher's Exact Test Results.** There are 19 RES chromosome + COW chromosome arm syntenies that were detected as significant from a Bonferroni-corrected one-sided Fisher's exact test[12] performed on blastp reciprocal best hit results between COW and RES.

Similar to the CFR-HCA analysis, applying Fisher's exact test to syntenies filtered using ortholog-ortholog conservation (edges, **eq. 12**) shows a negative correlation between t and the number of significant groups identified. Many of these are likely false positives; as for the CFR-HCA analysis a cutoff of t~0.5 balances sensitivity and specificity. Applying Fisher's exact test to the results of the ortholog network conservation score (nodes, **eq. 13**) suggest that $t \geq 0.15$ will return more significantly conserved groups than would be returned from performing Fisher's exact test on the raw orthologs alone.

Cutoff *t*	Number of BC-FET significant groups (ortholog-ortholog FET, equation 12)	Number of BC-FET significant groups (Ortholog network FET, equation 13)
0	101	13
0.05	90	19
0.1	81	18
0.15	77	18
0.2	75	18
0.25	74	17
0.3	71	19
0.4	56	19
0.5	37	14
0.6	17	14
0.7	6	9
0.8	3	5
0.9	0	2
1.0	0	0

Supplementary Table 11.3 | **COW-RES conservation scores significant groupings.** This table shows the number of significant COW-chromosome-arm/RES-chromosome interactions there were when performing Fisher's exact test on the ortholog-ortholog conservation score (equation 12) and on the ortholog network scores (equation 13).

11.3.4 COW-HCA network conservation score analysis

With the CFR-HCA and COW-RES test cases in hand, we applied the network conservation score to the unicellular outgroup species and ctenophores to test for macrosynteny conservation only between genes that share a common evolutionary history on single chromosomes. For the ortholog-ortholog conservation score we used cutoff of $t \geq 0.5$, meaning that the ortholog pair is present on the same chromosomes in at least half of the other species in the OrthoFinder analysis. Fisher's exact test recovered 18 significant groupings based on ortholog-ortholog conservation alone, four of which represent complete two fusion-with-mixing events that we analyzed in previous sections (A1a_x, A1a_y, Ea_x, Ea_y). In addition, the other five fusion-with-mixing events that we identified in previous sections (C1_y, F_y, G_x, L_y, and N_y) all had one _x or _y component that appeared as significant in the analysis. See **Supplementary Table 11.4**.

Performing Fisher's exact test on the gene network conservation scores using a cutoff threshold of t ≥ 0.35 produced a set of syntenic regions similar to those in **Supplementary Table 11.4**. The previously identified fusion-with mixing events A1a_x/A1a_y and Ea_x/Ea_y both had the _x and _y components present. The method also detected components for four additional previously-identified fusion-with-mixing events (C1_y, F_y, G_x, N_y). See **Supplementary Table 11.5**.

	COW	HCA	FET *p*-value	Number of Ortholog-ortholog connections t ≥ 0.5	Corresponding Group
1	COW3:1602947-end	HCA7:0-end	8.87×10^{-30}	47	A1a_x
2	COW5:0-1340158	HCA12:0-end	7.09×10^{-11}	12	A1a_y
3	COW3:0-1602947	HCA13:0-end	5.57×10^{-9}	16	B1_z1
4	COW14:491731-end	HCA5:0-end	5.97×10^{-25}	23	C1_y
5	COW3:1602947-end	HCA5:0-end	5.50×10^{-4}	19	C1_z2
6	COW5:1340158-end	HCA2:0-end	2.14×10^{-3}	11	C2
7	COW12:0-501091	HCA1:0-end	3.00×10^{-40}	54	D
8	COW3:0-1602947	HCA2:0-end	2.62×10^{-6}	23	Ea_x and C1_z1
9	COW4:792781-end	HCA8:0-end	6.08×10^{-8}	14	Ea_y
10	COW8:801448-end	HCA10:0-end	1.30×10^{-11}	11	F_y
11	COW10:0-606463	HCA2:0-end	4.19×10^{-11}	20	G_x
12	COW4:0-792781	HCA6:0-end	3.54×10^{-32}	50	H_z1
13	COW6:1488804-end	HCA6:0-end	6.16×10^{-12}	20	H_z2
14	COW11:0-804060	HCA11:0-end	1.29×10^{-10}	11	J2
15	COW6:0-1488804	HCA3:0-end	7.38×10^{-5}	16	K_z2
16	COW1:0-2810494	HCA9:0-end	3.25×10^{-9}	35	L_y
17	COW2:0-1545387	HCA13:0-end	1.31×10^{-5}	12	N_y
18	COW4:792781-end	HCA9:0-end	6.54×10^{-12}	29	None

Supplementary Table 11.4 | **COW-HCA ortholog-ortholog t ≥ 0.5 conservation score table.** The results of the Bonferroni-corrected one-sided Fisher's exact test of the ortholog-ortholog conservation scores for COW-HCA when $t \geq 0.5$. Rows colored red and blue are _x and _y components of previously identified fusion-with-mixing events. Yellow rows are _x or _y components from previously identified fusion-with-mixing events that lack their complementary _x or _y component.

	COW	HCA	FET p-value	Number of Ortholog network connections $t \geq 0.35$	Corresponding Group
1	COW3:1602947-end	HCA7:0-end	3.0×10^{-6}	19	A1a_x
2	COW5:0-1340158	HCA12:0-end	2.09×10^{-4}	9	A1a_y
3	COW14:0-491731	HCA5:0-end	7.05×10^{-3}	10	C1_y
4	COW14:491731-end	HCA5:0-end	4.71×10^{-4}	9	C1_y
5	COW12:0-501091	HCA1:0-end	1.5×10^{-5}	14	D
6	COW3:0-1602947	HCA2:0-end	2.69×10^{-3}	32	Ea_x and C1_z1
7	COW4:792781-end	HCA8:0-end	2.15×10^{-3}	17	Ea_y
8	COW8:801448-end	HCA10:0-end	5.12×10^{-3}	6	F_y
9	COW10:0-606463	HCA2:0-end	6.78×10^{-3}	15	G_x
10	COW4:0-792781	HCA6:0-end	4.9×10^{-5}	16	H_z1
11	COW11:0-804060	HCA11:0-end	4.99×10^{-2}	8	J2
12	COW2:0-1545387	HCA13:0-end	2.24×10^{-2}	9	N_y

Supplementary Table 11.5 | **COW-HCA ortholog network $t \geq 0.35$ conservation score table.** The results of the Bonferroni-corrected one-sided Fisher's exact test of the ortholog network conservation scores for COW-HCA when $t \geq 0.35$. Rows colored red and blue are _x and _y components of previously identified fusion-with-mixing events. Yellow rows are _x or _y components from previously identified fusion-with-mixing events that lack their complementary _x or _y component.

11.3.5 SRO-HCA network conservation score analysis

Applying the ortholog-ortholog method to the choanoflagellate *S. rosetta* and the ctenophore *H. californensis* also recovered syntenies from previously identified fusion-with-mixing events (A1a_x, A1a_y, G_x, G_y, N_x, N_y) with $t \geq 0.5$. The method found L_y as conserved between ctenophore and choanoflagellate, but not the complementary L_x group (**Supplementary Table 11.6**). Fisher's exact test on the ortholog network conservation scores $t \geq 0.35$ recovered several components from fusion-with-mixing events (A1a_x, L_y, and G_y) (**Supplementary Table 11.7**).

	SRO	HCA	FET	Number of Ortholog-ortholog connections t ≥ 0.5	Corresponding Group
1	SRO5:0-end	HCA7:0-end	1.41×10^{-16}	23	A1a_x
2	SRO3:0-end	HCA12:0-end	1.15×10^{-9}	16	A1a_y
3	SRO1:0-end	HCA5:0-end	4.98×10^{-3}	13	C1
4	SRO2:0-end	HCA1:0-end	8.70×10^{-5}	33	D
5	SRO1:0-end	HCA10:0-end	4.94×10^{-3}	7	F
6	SRO4:0-end	HCA2:0-end	7.03×10^{-4}	13	G_x
7	SRO17:0-end	HCA3:0-end	2.23×10^{-9}	12	G_y
8	SRO10:0-end	HCA6:0-end	2.19×10^{-12}	21	H
9	SRO25:0-end	HCA1:0-end	2.19×10^{-8}	15	I_z3
10	SRO9:0-end	HCA3:0-end	1.15×10^{-5}	14	K
11	SRO27:0-end	HCA9:0-end	1.59×10^{-11}	14	L_y
12	SRO16:0-end	HCA9:0-end	6.68×10^{-17}	23	M
13	SRO19:0-end	HCA6:0-end	9.34×10^{-7}	10	N_x
14	SRO2:0-end	HCA13:0-end	9.76×10^{-7}	17	N_y
15	SRO14:0-end	HCA7:0-end	2.65×10^{-2}	6	None
16	SRO3:0-end	HCA8:0-end	4.12×10^{-6}	13	None
17	SRO6:0-end	HCA4:0-end	2.03×10^{-5}	8	P

Supplementary Table 11.6 | **SRO-HCA ortholog-ortholog t ≥ 0.5 conservation score table.** The results of the Bonferroni-corrected one-sided Fisher's exact test of the ortholog-ortholog conservation scores for SRO-HCA when $t \geq 0.5$. Rows colored red and blue are _x and _y components of previously identified fusion-with-mixing events. Yellow rows are _x or _y components from previously identified fusion-with-mixing events that lack their complementary _x or _y component.

	SRO	HCA	FET	Number of Ortholog network connections t ≥ 0.35	Corresponding Group
1	SRO5:0-end	HCA7:0-end	3.00×10-6	18	A1a_x
2	SRO27:0-end	HCA9:0-end	2.09×10-4	8	L_y
3	SRO1:0-end	HCA10:0-end	7.05×10-3	14	F
4	SRO29:0-end	HCA9:0-end	4.71×10-4	7	None
5	SRO10:0-end	HCA6:0-end	1.50×10-5	13	H
6	SRO16:0-end	HCA9:0-end	2.69×10-3	12	M
7	SRO17:0-end	HCA3:0-end	2.15×10-3	10	G_y
8	SRO3:0-end	HCA8:0-end	5.12×10-3	15	None
9	SRO33:0-end	HCA11:0-end	6.78×10-3	3	None
10	SRO9:0-end	HCA3:0-end	1.15×10^{-5}	14	K
11	SRO27:0-end	HCA9:0-end	1.59×10^{-11}	14	L_y
12	SRO16:0-end	HCA9:0-end	6.68×10^{-17}	23	M

Supplementary Table 11.7 | **SRO-HCA ortholog network t ≥ 0.35 conservation score table.** The results of the Bonferroni-corrected one-sided Fisher's exact test of the ortholog network conservation scores for SRO-HCA when $t \geq 0.35$. Rows colored red and blue are _x and _y components of previously identified fusion-with-mixing events. Yellow rows are _x or _y components from previously identified fusion-with-mixing events that lack their complementary _x or _y component.

11.4 Discussion

In the conservation score analysis of *C. owczarzaki* vs. *H. californensis* and *C. owzarzaki* vs. *H. californensis*, scored by conservation of orthologs across eleven species from an OrthoFinder analysis, we recovered four of the previously identified ancestral linkage groups involved in fusion events (ALG_A1a, ALG_Ea, ALG_G, ALG_N). For the remaining three linkage groups shown in **Figs. 3** and **4** (ALG_C1, ALG_F, ALG_L), we identified at least one of the _x or _y components. This methodology independently finds the same outgroup-ctenophore linkage groups found by analysis of blastp rbh searches (**Extended Data Tab. 2**, **Supplementary Information 8**), and of species quartet analyses using OrthoFinder (**Extended Data Fig. 9**, **Supplementary Information 10**).

This methodology may prove useful to future studies that seek to identify gene linkages in distantly related organisms. Future work on this approach would benefit from automated cutoff value identification to reduce false positives in ortholog-ortholog conservation score detection.

12 GO enrichment analysis of ALGs conserved in Filozoans

12.1 Introduction

While the colocalization of functionally related genes on short chromosome stretches can be attributed to regulatory constraints[137], for example HOX clusters[138], it is less clear whether overlapping networks of regulatory constraint are sufficient to explain the preservation of chromosome-scale syntenies between animals described here[139]. In addition to hypothetical regulatory constraints, there are other general factors that could lead to deeply-conserved synteny. Specifically, synteny is broken by partial arm translocations. When a partial arm translocation first arises it will typically be in a heterozygous state with wild type chromosomes. Translocation heterozygotes, however, have reduced fertility[31,140] due to the production of unbalanced gametes. The reduced fertility of translocation heterozygotes, in turn, limits the fixation of translocations[31,32] that would disrupt synteny, especially in large populations where selection is most effective[31,32].

If regulatory constraints are important, we might expect functional relationships between the genes belonging to the same conserved linkage groups. We explored this possibility by performing gene ontology (GO) enrichment analyses.

12.2 Methods

Using the ODP script `odp_rbh_to_hmm`, we searched for the *H. sapiens* orthologs of the genes in the ALGs presented in this manuscript. As a search database, we used the proteins available on the NCBI genome page for GRCh38.p14 (https://www.ncbi.nlm.nih.gov/genome/?term=homo+sapiens). To test for GO enrichment we tested each ALG individually, and specifically the ancestral metazoan _x and _y components identified in this manuscript. For each ALG, we used PANTHER v17.0[104] to perform a GO enrichment analysis.

12.3 Results and Discussion

GO categories relating to several fundamental cellular processes (e.g., protein folding) were enriched, but the enrichment patterns did not appear for every ALG, similar to the findings of previous studies[12]. Of note, we found that the proto-filozoan group corresponding to ALG_J2 contained both proteins in the mitochondrial fatty acid beta-oxidation multienzyme complex (HADHA, HADHB), suggesting ancient origins and genome colocalization of genes involved in critical cellular processes. While it is possible that the colocalization of these genes on single chromosomes has been critical to their co-regulation since the ancestor of all filozoans, more in-depth studies on how chromosomal topology and long-range interactions may affect gene regulation are required. Future studies might thus focus on using tools of functional genomics in many animal species to test the biological relationship between the gene linkages reported in this study. Spreadsheets containing the results of the GO enrichment analyses are located in **Supplementary Data 3**.

13 Entropy of gene mixing analysis

13.1 Methods - Gene mixing analysis

13.1.1 Visualizing gene mixing of two groups of genes on single chromosomes

The fusion of ancestral linkage groups in a stem lineage can serve as shared derived characters (synapomorphies) of a clade. Broadly speaking there are two types of fusions: (1) those in which the fused groups become colocated on the same chromosome, but do not mix (e.g., in Robertsonian fusions of acrocentric chromosomes to form a metacentric chromosome) and (2) fusions that are followed by the mixing of previously-separated groups of genes[12]. Fusion-with-mixing provides a particularly powerful type of synapomorphy, because once the two ancestral groups have become intermixed, reversion to the ancestral state is highly unlikely (in the same sense that unmixing of two pigments dissolved in water is unlikely).

To visualize mixing, we simply plot the gene coordinates from the two gene linkage groups along the length of the chromosome on which they are mixed. This is implemented in the script `odp_rbh_plot_mixing`. The results are reported in **Figure 3** of the main text.

13.1.2 Quantifying the degree of mixing of two groups of genes on single chromosomes

To quantify the degree of mixing, consider the fusion of two ancestral linkage groups A and B. We can represent the fused chromosome as a string of As and Bs, where each gene is assigned a letter depending on the ancestral linkage group it belonged to before fusion. Genes that are not members of ALGs A or B are not considered. An end-end or Robertsonian fusion is then represented by "AAA…AAA BBB…BBB." In this unmixed configuration, A's are followed by A's as we move from left to right along the string, and B's are followed by B's except at the point of fusion, where we find A followed by B. If we consider the ensemble of possible random strings, however, the two-letter substrings will generally include AA, AB, BB, and BA in roughly equal number. The number of AB and BA then provides a measure of how mixed the two letters are in the string of A's and B's.

For a given string we define |A| and |B| as the number of A's and B's, so that |A|+|B|=N. Similarly, we define the number of each two-letter substring to be |..|, so that |AA|+|AB|+|BB|+|BA| is N-1. We define the mixing statistic as the number of AB and BA transitions divided by the expected number of transitions for a long random string, which is given approximately by 2|A| |B|/N -1:

We then define the mixing statistic by:

$$m = \frac{|AB| + |BA| - 1}{(2|A|\,|B|\,/\,N) - 1}$$

The -1 in the numerator accounts for obligatory transition present in the unmixed string "AAA…AAA BBB…BBB.", so this string has m=0. If |A|=|B| (that is, the two ancestral linkage groups are of equal size), then $m \leq 2$, with the maximum attained by the perfectly interleaved string "ABAB…". By definition, the average value of *m* for a long random string is 1.

For each fusion, we modeled the distribution of *m* values for complete mixing by generating random strings the corresponding $|A|$ and $|B|$. An empirical distribution for *m* for these random strings was built over 100000 iterations. We treated this distribution as a null model with complete mixing (random order of A and B). We used a one-tailed test to determine whether this null model should be rejected.

13.2 Results and Discussion - Gene mixing analysis

13.2.1 Degree of mixing between phylogenetically informative groups of linkage groups

Of the seven fusions uniting sponges, bilaterians, and cnidarians, we found that the four corresponding to ALGs C1, F, L, and N, were consistent with fusion-then-mixing (**Fig. 3**, *p*-values for the mixing test can be found in **Supplementary Data 4, 5**). The other three fusions (Ea, G, and A1a) show more limited evidence of mixing. Linkage groups Ea_x and Ea_y have no overlapping genes on chromosome 1 of both demosponges (*Ephydatia* and the cladorhizid), and so are unmixed in sponges, but mixed in bilaterians and cnidarians. Similarly, the ancestral components of ALGs G and A1a have only limited overlap between their respective _x and _y components, and so are unmixed in both demosponges by our criteria based on the statistic m above, but are mixed in bilaterians and cnidarians, suggesting that the G and A1a-bearing chromosomes in demosponges have only undergone a handful of rearrangements since the fusion of these respective ancestral metazoan linkage groups in the demosponge lineage.

13.2.2 Expanding membership of ancestral metazoan linkage groups

The requirement that orthogroups include an outgroup gene is a stringent one that limits both our ability to detect statistically-significant ancestral metazoan linkage groups and our power to test the null model of mixing described above. Having used this stringent criterion to establish ancestral linkage groups that are "split" in the same way in ctenophore and outgroups relative to other metazoans, we can expand the number of orthologs to use in statistical analyses of mixing by relying on metazoan orthogroups that may not contain genes from outgroups. We therefore used the three-way reciprocal best blastp search including *Hormiphora*, *Rhopilema*, and *Ephydatia* and use the methodology described in **Supplementary Information 5.2.3** to find 65 linkage groups conserved across these species comprising 1,272 genes (**Supplementary Data 2**, tab 9), and extended membership in these orthogroups to other metazoan species using the HMM search method (**Extended Data Figure 8**, **Supplementary Table 7.1)**. We then performed the gene mixing analysis described above on these more complete ALGs, subject to the condition that the ancestral metazoan state of these ALGs be supported by the outgroup genes that they do contain.

Using this expanded membership, we confirm that the linkage group pairs corresponding to A1a_x/A1a_y, G_x/G_y, and the Ea_x/Ea_y fusions are mixed across bilaterians and cnidarians (as found in the more stringent analysis including outgroups **Supplementary Information 13.2.1**). When the expanded linkage groups are considered, however, these pairs are also mixed in both demosponges (**Extended Data Figure 8**). In the main text, we use the more conservative analysis of **Supplementary Information 13.2.1** that includes outgroups, and consider the respective _x and _y components of A1a, G, and Ea to be unmixed in demosponges. Additional sequencing and analysis, however, may find evidence of these mixings, which would further strengthen our conclusion that sponges are united with bilaterians and cnidarians to the exclusion of ctenophores, by these additional irreversible synapomorphies.

14 Analyzing chromosomal tectonic events in a Bayesian phylogenetic framework

14.1 Introduction

We have shown that fusions of ancestral linkage groups can serve as phylogenetically informative characters. The value of these fusion characters can be easily appreciated within a parsimony framework that (1) minimizes the number of convergent fusions and (2) excludes reversions of fusions-with-mixing, since these are effectively irreversible[12]. In this way shared derived fusions, and shared derived fusions with mixing, provide robust synteny-based synapomorphies for identifying monophyletic clades.

Here we describe the construction of a character matrix based on linkage group fusions and its analysis in a Bayesian framework. Briefly, for each pair of ancestral linkage groups X and Y inferred using conserved linkages present in outgroups, there are three possible states for each species:

- State 0: X and Y are on different chromosomes, and therefore not fused.
- State 1: X and Y are on the same chromosome, and are unmixed by the criteria of **Supplementary Information 13**.
- State 2: X and Y are on the same chromosome, and are determined to be mixed by the criteria of **Supplementary Information 13**.

A simpler binary encoding combines States 0 and 1 above into a single state, since fusion without mixing (as realized by, e.g., a Robertsonian translocation) is in principle reversible, leading to the simpler two-state encoding

- State 0: X and Y are on different chromosomes, or are on the same chromosome but are unmixed by the criteria of **Supplementary Information 13**.
- State 1: X and Y are on the same chromosome, and are determined to be mixed by the criteria of **Supplementary Information 13**.

14.2 Methods

14.2.1 Constructing character matrices of chromosome fusion states

We produced two character matrices to encode the fusion states of the ALGs found in **Extended Data Table 1.** In total, we identified 162 unique ALG-ALG fusion events to be used as phylogenetic characters. We analyzed species COW, SRO, HCA, BIN, CLA, EMU, NVE, RES, BFL, PMA and excluded the *Trichoplax adhaerens* genome as its genome was not chromosome-scale. These character matrices can be found in **Supplementary Data 6.**

14.2.2 Bayesian phylogenetic analysis

We used RevBayes version 1.1.1[106] and MrBayes version 3.2.7a[52] to implement a binary "restriction" model that describes transitions from unfused or unmixed (0) to fused-with-mixing (1) state and vice versa. Both programs have a minimum character change probability (0.001) that is far larger than the extremely low probability of unmixing after fusion estimated in ref[12], so we used the lowest allowed value. This has the effect of making our calculations more conservative, since reversal of fusion-with-mixing is permitted In MrBayes we used state frequency priors using the following command:

```
prset statefreqpr=fixed(0.01,0.99)
```

MrBayes was run for 100,000 generations with a burn-in of the first 25% of the trees. The remainder of the trees were used to generate a consensus tree.

Similarly, in RevBayes we defined the instantaneous rates as:

```
rates := [ [ 0 ,      0.1 ], [0.001,    0  ] ]
```

This produces the instantaneous rate matrix (rescaled=false in fnFreeK):

```
Q := [ [ -0.1000, 0.1000 ] , [ 0.001, -0.001 ] ]
```

For branch length of 1, the resulting transition probabilities are:

```
P = [ [ 0.905, 0.095 ], [ 0.001, 0.999 ] ]
```

The rates were chosen to allow for an intermittent chance of fusion (e.g., 9.5% for branches of length 1, corresponding to the observed rate of fusions in the character matrix), yet a much lower (but still unrealistically high) chance of unmixing (0.1% for branch length of 1). In RevBayes we were also able to specify the root frequencies (1 for unmixed and 0 for mixed state), given that the ancestral (starting) state is by definition unmixed.

For the three-state model, because we do not have separate estimates for the rate of fusion and for subsequent mixing (or fission, in the absence of mixing), we tested a range of rates (0.01, 0.1, 0.2, 0.5) for both `0<->1` and `1->2` transitions (`2->1` transition rate was set again to the lowest non-zero value of 0.001).

RevBayes was run for 100,000 generations and the first 25% of the trees were used as burn-in, all runs converged.

14.3 Results and Discussion

Both RevBayes (three-state) and MrBayes (two-state) produced trees with ctenophores as sister to BCnS with posterior probability 1.00 for the monophyly of bilaterians, sponges, and cnidarians (**Supplementary Fig. 14.1**). This is to be expected based on arguments given in the main text, since the most parsimonious state transitions (as shown in main **Figs. 2 and 4**) are also the most likely series of state transitions in the more sophisticated probabilistic model. In the RevBayes trees, BFL and PMA (bilaterians), NVE and RES (cnidarians), HCA and BIN (ctenophores) each formed monophyletic groups with posterior probabilities between 0.99 and 1.0 (**Supplementary Fig. 14.1**). In both models, however, there is weak support for demosponge paraphyly, with EMU sister to bilaterians and cnidarians (posterior probability 0.47 (three-state, RevBayes) and 0.73 (two-state, MrBayes), and CLA sister to the EMU+bilaterian+cnidarian clade. This ambiguity in the relative placement of EMU and CLA arises in the Bayesian analysis of syntenic characters because (1) there are no clear sponge syntenic synapomorphies based on our outgroup-metazoan comparisons, and (2) CLA includes additional fusions that are not found in other animals, creating a long branch. The ambiguous relative placement of EMU and CLA, however, has no impact on the robust support for ctenophore-sister. Since demosponge monophyly is not in doubt based on other[8,10], in **Fig. 5 of** the main text we show the result of three-state RevBayes analysis with demosponge monophyly constrained. We note that changes to the instantaneous rate matrices, even by orders of magnitude, did not affect the relative branching order of ctenophores and sponges as long as the asymmetry between mixing and unmixing is included. Ctenophores are always the earliest diverging animal group.

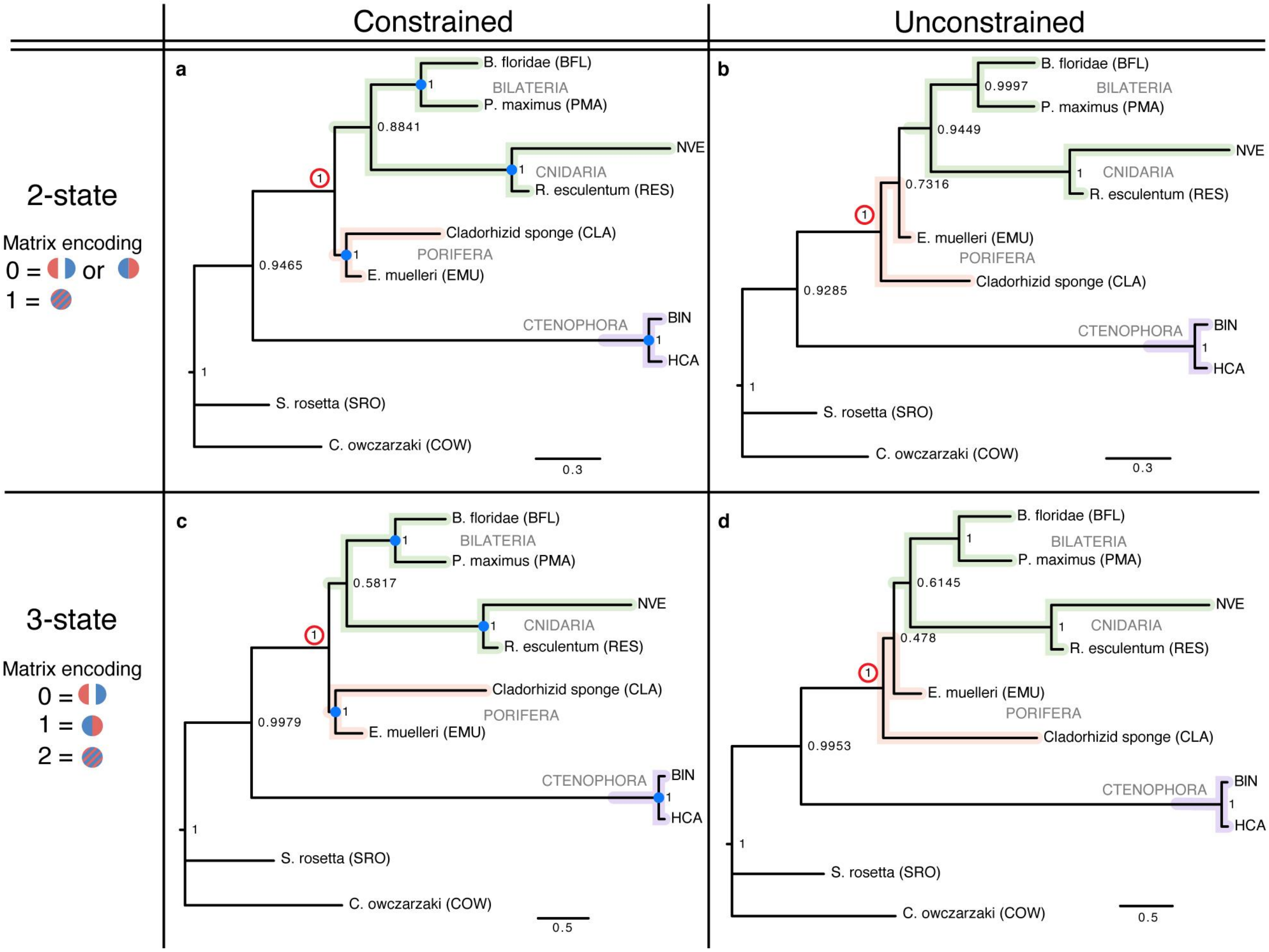

Supplementary Figure 14.1 | **Bayesian analysis of chromosome fusion states recovers ctenophore-sister.** Panels **a.-d.** show Bayesian phylogenetic trees inferred from a character matrix of gene linkage group fusion states. The panels show trees from two variables in the analysis conditions: trees with clade constraints (blue dots are constrained nodes - ctenophore monophyly enforced, sponge monophyly enforced, cnidarian monophyly enforced, and bilaterian monophyly enforced), and trees run under a 2-state or 3-state model. In each tree, the support values supporting sponge-sister or ctenophore-sister are circled in red. Each tree was run for 100,000 generations with 25% burn-in.

15 Null hypothesis testing of the ctenophore-sister topology

15.1 Introduction - Genome Shuffling Simulations

We considered whether the chromosomal data supporting the ctenophore-sister hypothesis could be explained by the highly rearranged state of the ctenophore genomes compared to the relatively stable genomes of other animal clades. We implemented a simulation to test whether the gene groups that we find supporting the ctenophore-sister hypothesis would also appear in randomly shuffled genomes.

15.2 Methods - Genome Shuffling Simulations

15.2.1 Design of genome shuffling simulation

One trial of the simulation consisted of the following steps. The gene labels in the *Hormiphora* genome annotation file were randomly shuffled. The chromosomes in the shuffled genome contain the same number of genes as the real *Hormiphora* genome. We refer to the shuffled genome with the prefix 's' in front of the three-letter code, i.e., sHCA is the shuffled *Hormiphora* genome. We used the sHCA genome to perform a COW-sHCA-EMU-RES four-way reciprocal best blastp search. The orthologs were grouped by the common chromosomes on which they occurred in the four species. We consider these to be (simulated) ancestral linkage groups. As in the main analysis, we used a false-discovery rate of 0.05, and a minimum of five genes in one linkage group, to remove linkage groups that contained so few genes that they could be explained by random chance. The remaining rows were used to identify phylogenetically informative (PI) fusion events: fusion events that supported either the ctenophore-sister or the sponge-sister hypothesis. From the PI events, we recorded the number of PI fusion events supporting the ctenophore-sister or sponge-sister hypotheses, the size of the largest linkage group in each PI event, the number of genes in single PI fusion events, and the total number of genes in PI events supporting either hypothesis. We performed the trial shuffling the *Hormiphora* genome 100 million times, and compared the distribution of the parameters we measured to what we observed in the COW-HCA-EMU-RES comparison with the real *Hormiphora* genome.

In additional computational experiments, we also shuffled the *Capsaspora*, *Ephydatia*, and *Rhopilema* genomes (sCOW-HCA-RES-EMU, COW-HCA-sRES-EMU, and COW-HCA-RES-sEMU), and we performed the corresponding simulations using SRO as the outgroup (sSRO-HCA-RES-EMU, SRO-sHCA-RES-EMU, COW-HCA-sRES-EMU, and COW-HCA-RES-sEMU). These results are shown in main **Figure 5** and **Extended Data Fig. 10.**

15.3 Results and Discussion - Genome Shuffling Simulations

15.3.1 Genome shuffling simulation results

In all eight simulations, all measured parameters were much higher in the real genomes than when one of the genomes was randomly shuffled (**Extended Data Fig. 10**). For example, the observed number of genes supporting the ctenophore-sister hypothesis in the COW-HCA-EMU-RES searches (using actual genomes) was 67. In the four shuffling simulations using these four species, the maximum number of genes supporting ctenophore-sister (in 400 million individual trials) was only 11, far less than the 67 observed (**Extended Data Fig. 10a-d**, "Gene count of all PI FLGs"). This simulation bounds the false discovery rate (of data as strong as the observed result with the actual genomes) based on of the number of trials performed in each simulation ($\alpha < 1\times10^{-8}$).

Remarkably, none of the 800 million trials (shuffling each species in the quartets with COW or SRO as outgroups) had any (simulated) linkage groups supporting the sponge-sister hypothesis. This is perhaps not surprising given the highly conserved karyotype between sponges, cnidarians, and bilaterians (**Fig. 1, Extended Data Fig. 2**).

These results are strong evidence that the linkage groups that we see conserved between ctenophores, sponges, cnidarians, and unicellular outgroup species are not the result of random linkages arising from the highly rearranged genome configuration found in ctenophores, but are truly relicts of ancestral linkage groups that have been present on single chromosomes since the common ancestor of metazoan and the outgroups.

16 Supplementary Information References

Please see the main manuscript for the references cited there. The references below were cited only in the Supplementary Information.

108. Imakaev, M. *et al.* Iterative correction of Hi-C data reveals hallmarks of chromosome organization. *Nat. Methods* **9**, 999–1003 (2012).
109. Redmond, N. E. *et al.* Phylogeny and systematics of demospongiae in light of new small-subunit ribosomal DNA (18S) sequences. *Integr. Comp. Biol.* **53**, 388–415 (2013).
110. Patry, W. L., Bubel, M., Hansen, C. & Knowles, T. Diffusion tubes: a method for the mass culture of ctenophores and other pelagic marine invertebrates. *PeerJ* **8**, e8938 (2020).
111. Dawson, M. N., Raskoff, K. A. & Jacobs, D. K. Field preservation of marine invertebrate tissue for DNA analyses. *Mol. Mar. Biol. Biotechnol.* **7**, 145–152 (1998).
112. Sambrook, J. & Russell, D. W. Purification of nucleic acids by extraction with phenol:chloroform. *CSH Protoc.* **2006**, db–prot4455 (2006).
113. Rio, D. C., Ares, M., Jr, Hannon, G. J. & Nilsen, T. W. Purification of RNA using TRIzol (TRI reagent). *Cold Spring Harb. Protoc.* **2010**, db.prot5439 (2010).
114. Buchfink, B., Xie, C. & Huson, D. H. Fast and sensitive protein alignment using DIAMOND. *Nat. Methods* **12**, 59–60 (2015).
115. Durand, N. C. *et al.* Juicer Provides a One-Click System for Analyzing Loop-Resolution Hi-C Experiments. *Cell Syst* **3**, 95–98 (2016).
116. Robinson, J. T. *et al.* Integrative genomics viewer. *Nat. Biotechnol.* **29**, 24 (2011).
117. Schultz, D. T. *et al.* Conserved novel ORFs in the mitochondrial genome of the ctenophore *Beroe forskalii*. *PeerJ* **8**, e8356 (2020).
118. Ekins, M., Erpenbeck, D. & Hooper, J. N. A. Carnivorous sponges from the Australian Bathyal and Abyssal zones collected during the RV Investigator 2017 Expedition. *Zootaxa* **4774**, zootaxa.4774.1.1 (2020).
119. Varoquaux, N. *et al.* Accurate identification of centromere locations in yeast genomes using Hi-C. *Nucleic Acids Res.* **43**, 5331–5339 (2015).
120. Knight, P. A. & Ruiz, D. A fast algorithm for matrix balancing. *IMA J. Numer. Anal.* **33**, 1029–1047 (2012).
121. Bredeson, J. V. *et al.* Conserved chromatin and repetitive patterns reveal slow genome evolution in frogs. *bioRxiv* 2021.10.18.464293 (2021) doi:10.1101/2021.10.18.464293.
122. Shalchian-Tabrizi, K. *et al.* Multigene phylogeny of choanozoa and the origin of animals. *PLoS One* **3**, e2098 (2008).
123. Douzery, E. J. P., Snell, E. A., Bapteste, E., Delsuc, F. & Philippe, H. The timing of eukaryotic evolution: does a relaxed molecular clock reconcile proteins and fossils? *Proc. Natl. Acad. Sci. USA* **101**, 15386–15391 (2004).
124. Moreno-Hagelsieb, G. & Latimer, K. Choosing BLAST options for better detection of orthologs as reciprocal best hits. *Bioinformatics* **24**, 319–324 (2008).
125. Hoencamp, C. *et al.* 3D genomics across the tree of life reveals condensin II as a determinant of architecture type. *Science* **372**, 984–989 (2021).
126. Frönicke, L., Wienberg, J., Stone, G., Adams, L. & Stanyon, R. Towards the delineation of the ancestral eutherian genome organization: comparative genome maps of human and the African elephant (*Loxodonta africana*) generated by chromosome painting. *Proc. Biol. Sci.* **270**, 1331–1340 (2003).
127. Searle, J. B. & Wójcik, J. M. Chromosomal evolution: the case of *Sorex araneus*. in *Evolution of Shrews* (eds. Wójcik, J. M. & Wolsan, M.) 219–268 (Mammal Research Institute, Polish Academy of Sciences, 1998).

128. Larson, A., Prager, E. M. & Wilson, A. C. Chromosomal evolution, speciation and morphological change in vertebrates: the role of social behaviour. in *Chromosomes Today: Volume 8 Proceedings of the Eighth International Chromosome Conference held in Lübeck, West Germnay, 21–24 September 1983* (eds. Bennett, M. D., Gropp, A. & Wolf, U.) 215–228 (Springer Netherlands, 1984).
129. Laumer, C. E. *et al.* Support for a clade of Placozoa and Cnidaria in genes with minimal compositional bias. *Elife* **7**, (2018).
130. Pandey, A. & Braun, E. L. Phylogenetic Analyses of Sites in Different Protein Structural Environments Result in Distinct Placements of the Metazoan Root. *Biology* **9**, (2020).
131. Syed, T. & Schierwater, B. *Trichoplax adhaerens*: Discovered as a missing link, forgotten as a hydrozoan, re-discovered as a key to Metozoan evolution. *Vie et Milieu/Life & Environment* 177–187 (2002).
132. Wall, D. P., Fraser, H. B. & Hirsh, A. E. Detecting putative orthologs. *Bioinformatics* **19**, 1710–1711 (2003).
133. Wolf, Y. I. & Koonin, E. V. A tight link between orthologs and bidirectional best hits in bacterial and archaeal genomes. *Genome Biol. Evol.* **4**, 1286–1294 (2012).
134. Dalquen, D. A. & Dessimoz, C. Bidirectional best hits miss many orthologs in duplication-rich clades such as plants and animals. *Genome Biol. Evol.* **5**, 1800–1806 (2013).
135. Nadeau, J. H. & Taylor, B. A. Lengths of chromosomal segments conserved since divergence of man and mouse. *Proc. Natl. Acad. Sci. USA* **81**, 814–818 (1984).
136. Sankoff, D. & Ferretti, V. Karyotype distributions in a stochastic model of reciprocal translocation. *Genome Res.* **6**, 1–9 (1996).
137. Montavon, T. & Duboule, D. Chromatin organization and global regulation of Hox gene clusters. *Philos. Trans. R. Soc. Lond. B Biol. Sci.* **368**, 20120367 (2013).
138. Carroll, S. B. Homeotic genes and the evolution of arthropods and chordates. *Nature* **376**, 479–485 (1995).
139. Ghavi-Helm, Y. *et al.* Highly rearranged chromosomes reveal uncoupling between genome topology and gene expression. *Nat. Genet.* **51**, 1272–1282 (2019).
140. White, M. J. D. *Animal cytology & evolution*. (Cambridge university press, 1954).
