# Supplementary material for: Ancient gene linkages support ctenophores as sister to other animals
Source: Nature. 2023 May 17;618(7963):110–7. doi: 10.1038/s41586-023-05936-6 (PMC10232365; doi:10.1038/s41586-023-05936-6)
Supplement: Supplementary file 2 — Reporting Summary [file 41586_2023_5936_MOESM2_ESM.pdf]

nature portfolio

Corresponding author(s): Darrin T. Schultz, Oleg Simakov, Daniel S. Rokhsar

Last updated by author(s): Feb 27, 2023

Reporting Summary

Nature Portfolio wishes to improve the reproducibility of the work that we publish. This form provides structure for consistency and transparency in reporting. For further information on Nature Portfolio policies, see our Editorial Policies and the Editorial Policy Checklist.

Statistics

For all statistical analyses, confirm that the following items are present in the figure legend, table legend, main text, or Methods section.

n/a	Confirmed	
☐	☒	The exact sample size (*n*) for each experimental group/condition, given as a discrete number and unit of measurement
☐	☒	A statement on whether measurements were taken from distinct samples or whether the same sample was measured repeatedly
☐	☒	The statistical test(s) used AND whether they are one- or two-sided<br>*Only common tests should be described solely by name; describe more complex techniques in the Methods section.*
☐	☒	A description of all covariates tested
☐	☒	A description of any assumptions or corrections, such as tests of normality and adjustment for multiple comparisons
☐	☒	A full description of the statistical parameters including central tendency (e.g. means) or other basic estimates (e.g. regression coefficient) AND variation (e.g. standard deviation) or associated estimates of uncertainty (e.g. confidence intervals)
☐	☒	For null hypothesis testing, the test statistic (e.g. *F*, *t*, *r*) with confidence intervals, effect sizes, degrees of freedom and *P* value noted<br>*Give P values as exact values whenever suitable.*
☐	☒	For Bayesian analysis, information on the choice of priors and Markov chain Monte Carlo settings
☒	☐	For hierarchical and complex designs, identification of the appropriate level for tests and full reporting of outcomes
☒	☐	Estimates of effect sizes (e.g. Cohen's *d*, Pearson's *r*), indicating how they were calculated

Our web collection on statistics for biologists contains articles on many of the points above.

Software and code

Policy information about availability of computer code

Data collection	The software developed for this manuscript is available at https://github.com/conchoecia/odp and at https://doi.org/10.5061/dryad.dncjsxm47.
Data analysis	The software packages used to analyze data in this manuscript were: odp v0.2.0 and v0.3.0, HiRise vAug2019, SALSA2 v2.3, wtdbg v2.4, hifiasm v0.16.1-r375, TGS-Gapcloser v1.1.1, Purge Haplotigs v1.0.4, pilon v1.23, Diamond v0.9.24, Blobtools v1.0, minimap2 v2.17 and v2.23, tblastn v2.10.0+, miniprot v0.2, BRAKER v2.14, , STAR v2.7.1a, ProtHint v2.6.0, Trinity v2.5.1, OrthoFinder v2.3.7 , hmmer v3.3.2, PANTHER v17, snakemake v7, BUSCO v5, lima v2.2.0, isoseq3 v3.4.0, jellyfish v2.2.10, GenomeScope 2, bwa mem v0.7.17, PretextView v0.2.4, HiGlass v1.10.0104, Juicebox Assembly Tools github commit 46c7ed1105, Juicebox visualization system v1.11.08106, TransDecoder v5.5, D-genies v1.4.0, pairtools v0.3.0 pairix v0.3.7, Cooler v0.8.10, MAFFT v7.310, RevBayes version 1.1.1, MrBayes version 3.2.7a, and FigTree v1.4.4.

For manuscripts utilizing custom algorithms or software that are central to the research but not yet described in published literature, software must be made available to editors and reviewers. We strongly encourage code deposition in a community repository (e.g. GitHub). See the Nature Portfolio guidelines for submitting code & software for further information.

Data

Policy information about availability of data

All manuscripts must include a data availability statement. This statement should provide the following information, where applicable:

- Accession codes, unique identifiers, or web links for publicly available datasets
- A description of any restrictions on data availability
- For clinical datasets or third party data, please ensure that the statement adheres to our policy

The authors confirm that all data presented in this manuscript are available in public repositories. The sequencing reads are available in the NCBI database under BioProject accession numbers PRJNA818620, PRJNA818630, PRJNA903214, and PRJNA818537. The genomes for each species are available through the above BioProjects, with the exception that the genomes of C. fragrantissima, C. owczarzaki, and S. rosetta, which are available on Dryad: https://doi.org/10.5061/dryad.dncjsxm47. The scripts and results of the supplementary information, when not contained in figures, are also available in the aforementioned Dryad repository. Publicly available sequencing data and genomes were downloaded from NCBI from BioProject accession numbers PRJNA168, PRJDB8655, PRJNA12874, PRJNA20249, PRJNA20341, PRJEB28334, PRJNA30931, PRJNA31257, PRJNA37927, PRJEB56075, PRJEB56892, PRJNA64405, PRJNA193541, PRJNA193613, PRJNA213480, PRJNA278284, PRJNA281977, PRJNA377365, PRJNA396415, PRJNA512552, PRJNA544471, PRJNA576068, PRJNA579531, PRJNA625562, PRJNA667495, PRJNA761294, and PRJNA814716. The Ephydatia muelleri genome was downloaded from https://spaces.facsci.ualberta.ca/ephybase/. All of the above information is also included in the main text of the manuscript.

Human research participants

Policy information about studies involving human research participants and Sex and Gender in Research.

Reporting on sex and gender	N/A
Population characteristics	N/A
Recruitment	N/A
Ethics oversight	N/A

Note that full information on the approval of the study protocol must also be provided in the manuscript.

Field-specific reporting

Please select the one below that is the best fit for your research. If you are not sure, read the appropriate sections before making your selection.

☒ Life sciences ☐ Behavioural & social sciences ☐ Ecological, evolutionary & environmental sciences

For a reference copy of the document with all sections, see nature.com/documents/nr-reporting-summary-flat.pdf

Life sciences study design

All studies must disclose on these points even when the disclosure is negative.

Sample size	These categories are not applicable to this study, which involves analysis of genome sequences and chromosome architecture. Sample sizes of permutation tests and the resulting statistics are included in the manuscript.
Data exclusions	DNA sequences were filtered (for quality) during the assembly process as described in the methods.
Replication	Our findings were replicated by (1) performing many analyses using combinations of different species, and (2) performing the same range of analyses using multiple orthology inference techniques. Otherwise, because this was a comparative genomics study using replicates of the same was not relevant.
Randomization	Randomization is not relevant to this study, as the genomes used in comparisons are selected at the point of experimental design. It is not possible to randomize these selections as we are dependent on the phylogenetic relationships between the species in the comparisons to draw conclusions.
Blinding	As above, the conclusions of this comparative genomics study were dependent on selecting groups of species with particular phylogenetic relationships. Therefore, blinding was not relevant to this study.

Reporting for specific materials, systems and methods

We require information from authors about some types of materials, experimental systems and methods used in many studies. Here, indicate whether each material, system or method listed is relevant to your study. If you are not sure if a list item applies to your research, read the appropriate section before selecting a response.

Materials & experimental systems

n/a	Involved in the study
☒	☐ Antibodies
☒	☐ Eukaryotic cell lines
☒	☐ Palaeontology and archaeology
☐	☒ Animals and other organisms
☒	☐ Clinical data
☒	☐ Dual use research of concern

Methods

n/a	Involved in the study
☒	☐ ChIP-seq
☒	☐ Flow cytometry
☒	☐ MRI-based neuroimaging

Animals and other research organisms

Policy information about studies involving animals; ARRIVE guidelines recommended for reporting animal research, and Sex and Gender in Research

Laboratory animals

No laboratory animals were used in this study.

Wild animals

Samples of adult Bolinopsis microptera were collected on May 24th, 2015 in the Monterey Bay, California (36.63°N, 121.90°W) with jars from the surface waters, with permission under the State of California Department of Fish and Wildlife collecting permit SC-2026 to the Monterey Bay Aquarium. These samples were transported to the Monterey Bay Aquarium for rearing.

One individual of an undescribed hexactinellid sponge was collected on June 1st, 2021, in the Monterey Bay, California (34.57°N, 122.56°W) from the seafloor at 3,852 meters depth using the MBARI ROV Doc Ricketts aboard the R/V Western Flyer. On the following day one individual of an undescribed bioluminescent cladorhizid sponge was collected from a nearby site (35.49°N, 124°W) from the seafloor at 3,975 meters depth. The collection temperature of both samples was 1.5°C. The cladorhizid sample was consistent in morphology and locale with previously reported bioluminescent, carnivorous, cladorhizid sponges (1). Upon retrieval from the ROV, the samples were washed gently with 1°C filtered seawater to remove debris and maintained in the dark at 1°C for no more than 30 minutes after being collected from the ROV. Then, both samples were flash-frozen in liquid nitrogen. The samples were collected with the State of California Department of Fish and Wildlife collecting permit SC-4029 granted to the Haddock Laboratory at the Monterey Bay Aquarium Research Institute.

(1) Martini, S., Schultz, D. T., Lundsten, L. & Haddock, S. H. D. Bioluminescence in an Undescribed Species of Carnivorous Sponge (Cladorhizidae) From the Deep Sea. Front. Mar. Sci. 7, 576476 (2020).

Reporting on sex

N/A. The organisms sequenced in this study are hermaphrodites, or the biology of the species' sex is unknown.

Field-collected samples

A community culture was founded with 20 Bolinopsis microptera individuals in pseudokreisel tanks and diffusion tubes in 12°C seawater at the Monterey Bay Aquarium in Monterey, California. The culture was reared according to the published protocol (1) for three generations, and an F3 adult, called Bmic1, was selected and flash-frozen in liquid nitrogen on November 18th, 2019 for DNA sequencing for genome assembly and annotation. Four other F3 adults were placed into a spawning tank and spawned according to the published protocol(1). Fertilized eggs were collected 18 hours post-spawning for RNA sequencing.

(1) Patry, W. L., Bubel, M., Hansen, C. & Knowles, T. Diffusion tubes: a method for the mass culture of ctenophores and other pelagic marine invertebrates. PeerJ 8, e8938 (2020).

Ethics oversight

No vertebrates or cephalopods were used in the study, and the organisms included in this study are unrestricted other than the collecting permits listed above.

Note that full information on the approval of the study protocol must also be provided in the manuscript.
