# Supplementary figures and images for: Ancient gene linkages support ctenophores as sister to other animals
Source: Nature. 2023 May 17;618(7963):110–7. doi: 10.1038/s41586-023-05936-6 (PMC10232365; doi:10.1038/s41586-023-05936-6)

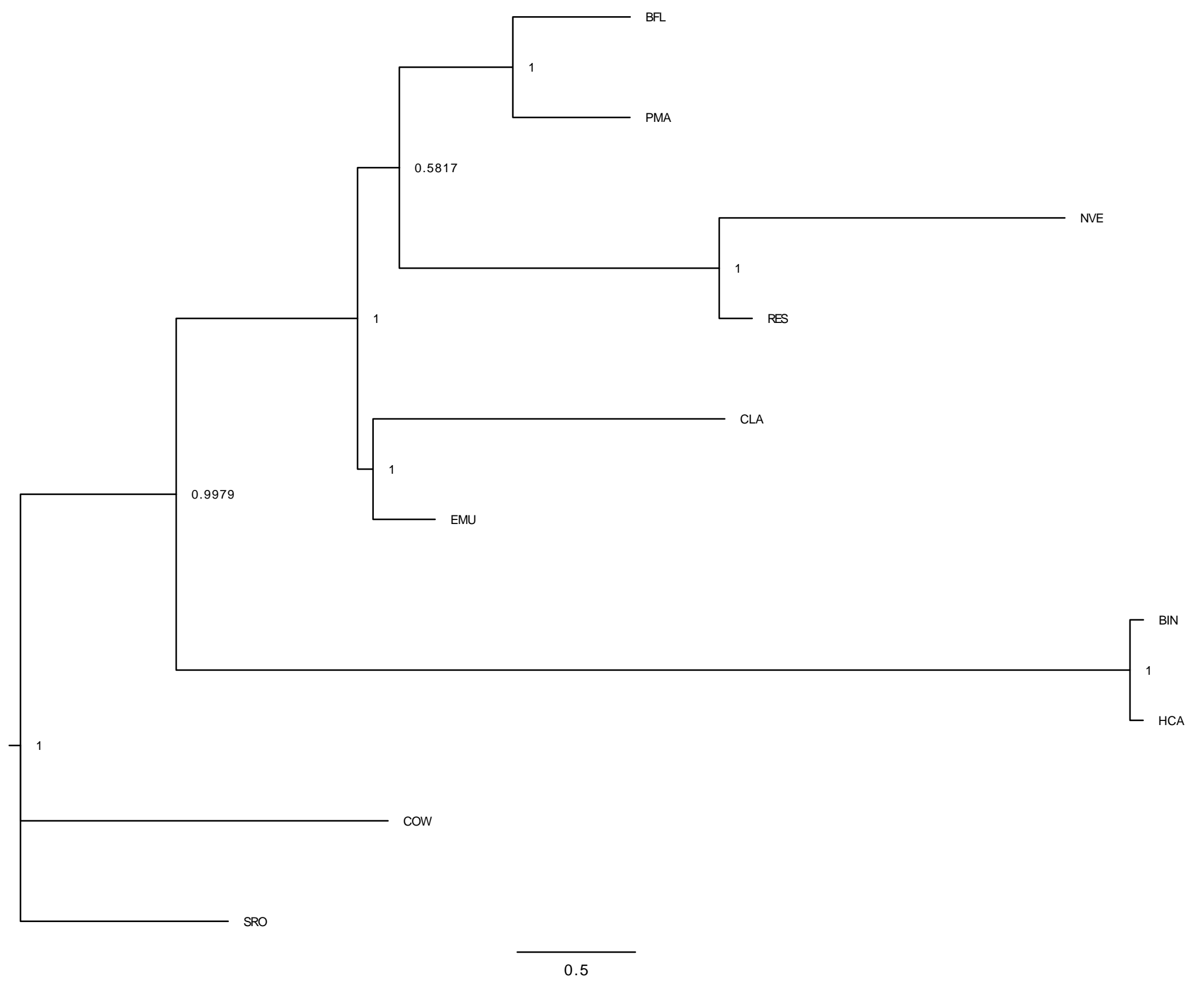

Supplement: Supplementary file 8 — Bayesian analysis files. [file 41586_2023_5936_MOESM8_ESM.zip › Schultzetal2022_Supplementary_Data_6/threeStateTrees/three_state_clade_Allconstrained.tree.pdf]

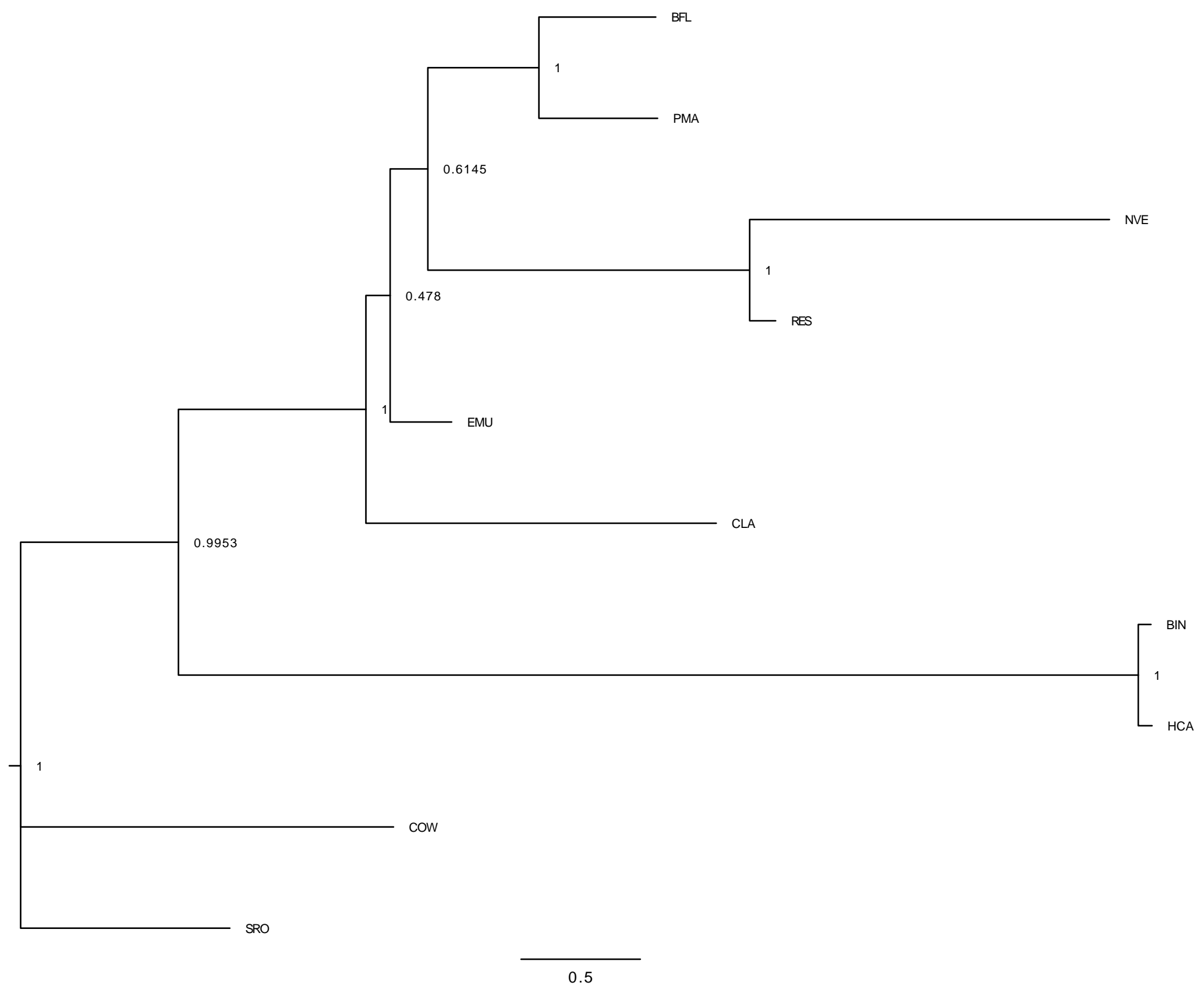

Supplement: Supplementary file 8 — Bayesian analysis files. [file 41586_2023_5936_MOESM8_ESM.zip › Schultzetal2022_Supplementary_Data_6/threeStateTrees/three_state_unconstrained.tree.pdf]

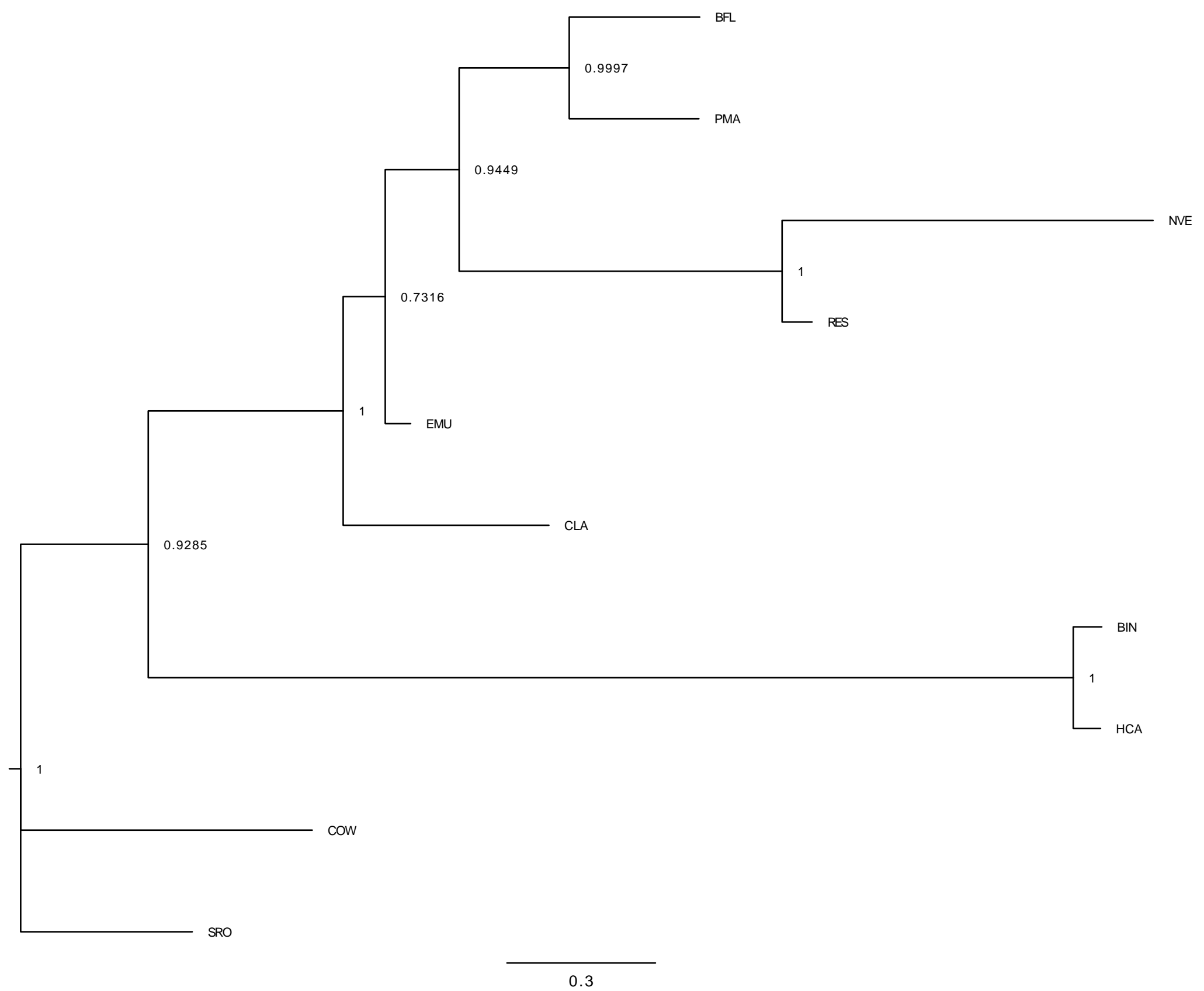

Supplement: Supplementary file 8 — Bayesian analysis files. [file 41586_2023_5936_MOESM8_ESM.zip › Schultzetal2022_Supplementary_Data_6/twoStateTrees/consensus_binary.map.unconstrained.tree.pdf]

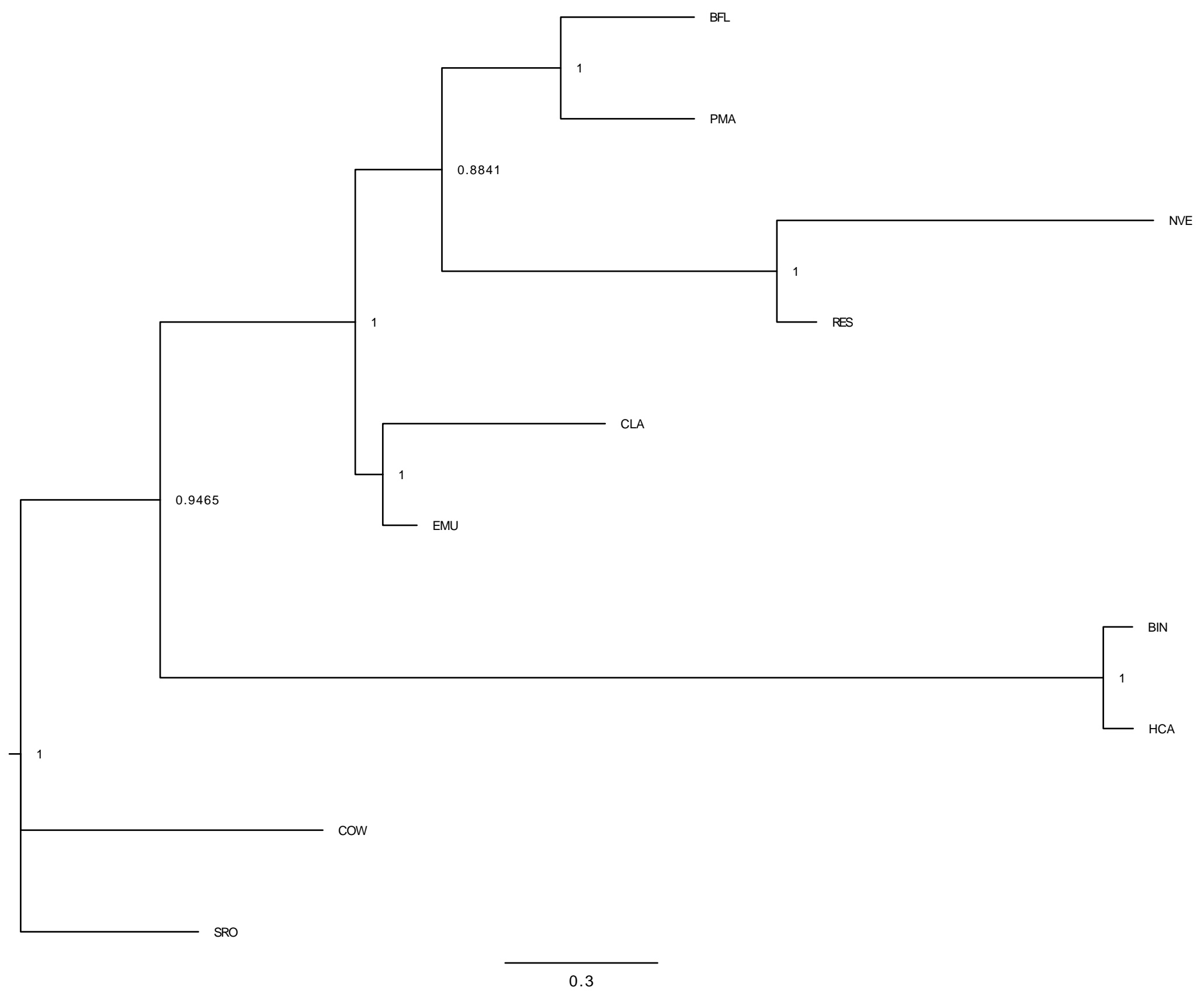

Supplement: Supplementary file 8 — Bayesian analysis files. [file 41586_2023_5936_MOESM8_ESM.zip › Schultzetal2022_Supplementary_Data_6/twoStateTrees/consensus_binary.map.allFixed.tree.pdf]
